# Advances in Strategies for Colloidal Self-Assembly

**DOI:** 10.1021/acs.chemrev.5c00692

**Published:** 2026-03-31

**Authors:** Chang Li, Dan Guo, Huanqing Cui, Ruotong Zhang, Huifeng Du, Kaixuan Li, Tiantian Yang, Tianrui Zhai, Yanlin Song, Ho Cheung Shum

**Affiliations:** † Advanced Biomedical Instrumentation Center, 26680 Hong Kong Science Park, Shatin, New Territories, Hong Kong (SAR), China; ⊥ Department of Mechanical Engineering, Faculty of Engineering, The University of Hong Kong, Hong Kong (SAR), China; ‡ School of Physics and Optoelectronic Engineering, 12496 Beijing University of Technology, Beijing 100124, P. R. China; § College of Materials Science and Engineering, 47890 Shenzhen University, Shenzhen 518060, China; ∥ Key Laboratory of Green Printing, Institute of Chemistry, Chinese Academy of Sciences, Beijing 100190, P. R. China; $ Department of Mechanical Engineering, Faculty of Engineering, 53030 The Hong Kong Polytechnic University, Hong Kong (SAR), China; & Department of Biomedical Engineering & Department of Chemistry, City University of Hong Kong, Hong Kong (SAR), China

## Abstract

The colloidal self-assembly organizes micro/nano-sized building blocks into large objects with periodic, quasi-periodic, or disordered structures. The obtained entities are functionalizable with smart building blocks and matrices, as well as the nanoscale symmetry, orientation, phase, and dimension of the structures. Various functionalizations of colloidal self-assemblies promise their applications in many areas, such as energy, microrobots, biomedicine, and communications. Given the importance, the methodology underlying colloidal self-assembly is highly required to be systematically analyzed. This review aims to comprehensively summarize advances in the techniques of colloidal self-assembly. For each strategy, we discuss the resultant colloidal structures, advantages, and limitations. The benefits of the new assemblies over the existing ones are also highlighted. Diverse driving forces and general characterization approaches are briefly introduced to help readers understand the self-assembly techniques. The review concludes with a summary of the challenges in colloidal self-assembly and a perspective on further advances in this field.

## Graphical abstract

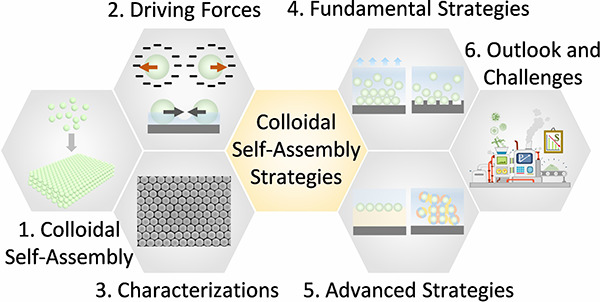

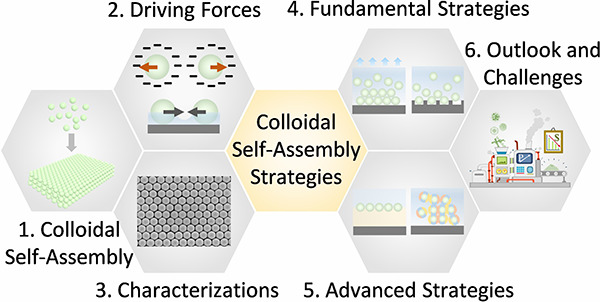

## 1 Introduction

Colloidal self-assembly is a process in which tiny building blocks organize into periodic, quasi-periodic, or nonperiodic structures, spontaneously increasing entropy.^1,2^ Although it appears counterintuitive, self-assembly prevails in nature since the majority of biostructures in organisms arise from self-assembly.^3^ For instance, the colors of peacock feathers^4^ and chameleons^5^ derive from self-organized periodic structures in their body. Similar examples include opal stones, which exhibit iridescent color due to the inside periodically stacked nanoparticles.^6^ Besides iridescence, self-assembled biostructures endow natural organisms with talents such as high mechanical strength,^7–9^ the ability to manipulate body temperature,^10^ and the potential to transport water directionally.^11^ Additionally, cells also offer countless examples of functional assemblies that create inspiring designs of active matter^12,13^ These examples illustrate the promising applications of self-assemblies, facilitating the development of the construction of artificial macron-/micron-scaled secondary structures.^14–16^

Colloidal self-assembly is intriguing as one of the bottom-up methods to construct artificial macron-/micron-scaled structures for the following reasons: First, self-assembly theoretically can induce crystallization at all scales.^17^ Various building blocks with sizes ranging from nanometers to micrometers can be used, and thus, the strategy is not restricted in terms of its resolution. Additionally, self-assembly is an attractive alternative for fabricating nanostructures due to its easy accessibility and low cost.

Second, self-assembled entities can be functionalized by smart building blocks or matrices in colloidal systems. Colloidal building blocks are nano- or microsized particles with specific shapes, polydispersity, and capping ligands that can well-disperse in a solvent. Generally, the colloidal building blocks are spherical nanoparticles with homogeneous surface properties. Various assembly strategies can organize the isotropic nanoparticles into different forms with the periodic appearance of the dielectric unit. Because of the periodicity, colloidal self-assemblies feature photonic bandgaps that can manipulate light propagation and thus are known as photonic crystals.^18^ The ability to manipulate light propagation allows colloidal crystals to be employed in structural colors,^19,20^ smart windows,^21,22^ visual sensors,^23–25^ Besides, self-assembled structures can be replicated into optoelectronic devices to improve performance as they can enhance the interaction between light and photoactive materials.^26,27^ In addition, by designing the wettability and packing state of the colloidal building blocks, self-assembled nanostructures can modify the interfacial^28,29^ and mechanical properties^30,31^ of materials. Incorporating biocompatible materials, such as hydrogels, enables the self-assembled structures to serve as a scaffold for cell culture^32,33^ or drug delivery^34,35^ due to their interconnectivity and porousness.^36^ Beyond isotropic spherical nanoparticles, anisotropic building blocks also facilitate the fabrication of complex colloidal self-assemblies, enriching structural and functional diversities of artificial superstructures.^37,38^

Third, the underlying principles of the colloidal self-assembly process may inspire the exploration of many fundamental subjects, such as cell organization and biostructure formation. In a self-assembly process, order emerges from disorder. The colloidal structure is generally seen because of a balance of interactions among the building blocks. However, how energy dissipation leads to the formation of ordered structures remains poorly understood. This process of energy evolution is essential in nature, where macromolecules, granules, or cells naturally and selectively gather to form organelles and organs. Studying colloidal self-assembly could offer insights into how living systems create microstructures and cell clusters. The colloidal self-assembly process is also related to many fundamental phenomena, including phase transition, mass transfer, and interaction among different materials. Thus, exploring the dynamic process of various colloidal self-assembly strategies might deepen the understanding of fundamental physics and material sciences.

The reasons outlined above highlight the importance of investigating colloidal self-assembly, as summarized previously.^38–53^ However, most of them emphasize one aspect of this topic, such as the driving forces,^39,41,50^ functional materials used for self-assembly,^38,39,48^ the functions of the resulting structures,^24,25,41^ and dynamic self-assembly (Table 1).^46,47,49,53^ This review provides a structured framework that categorizes techniques according to their scalability considerations and operational principles, and emphasizes the interplay of process parameters, particle characteristics, and substrate properties across techniques to guide practical implementation. This review primarily focuses on colloidal systems adsorbed on solid or liquid interfaces and elaborates on fundamental and advanced colloidal self-assembly techniques. Fundamental techniques that enable the assembly of isotropic colloidal building blocks spontaneously offer limited structural programmability, which are categorized by the dimensionality of their products: (1) Self-assembly of colloidal crystal dots; (2) self-assembly of colloidal crystal lines; (3) self-assembly of colloidal crystal films. Advanced colloidal self-assembly techniques involve a high degree of freedom to regulate the resulting colloidal structures in macro- and microsize. They include: (1) Three-dimensional (3D) printing colloidal self-assembly; (2) interfacial colloidal self-assembly; (3) external field-directed colloidal assembly; (4) nanofabrication-assisted colloidal self-assembly; (5) self-assembly of anisotropic colloidal particles; and (6) nature-mimetic self-assembly. For each strategy, we discuss the resultant structures, advantages, and limitations, highlighting improvements over existing approaches. To contextualize these techniques, we briefly introduce fundamental driving forces and common characterization methods. Finally, current challenges and perspectives toward improving colloidal self-assembly are discussed (Figure 1).

**Figure 1:**
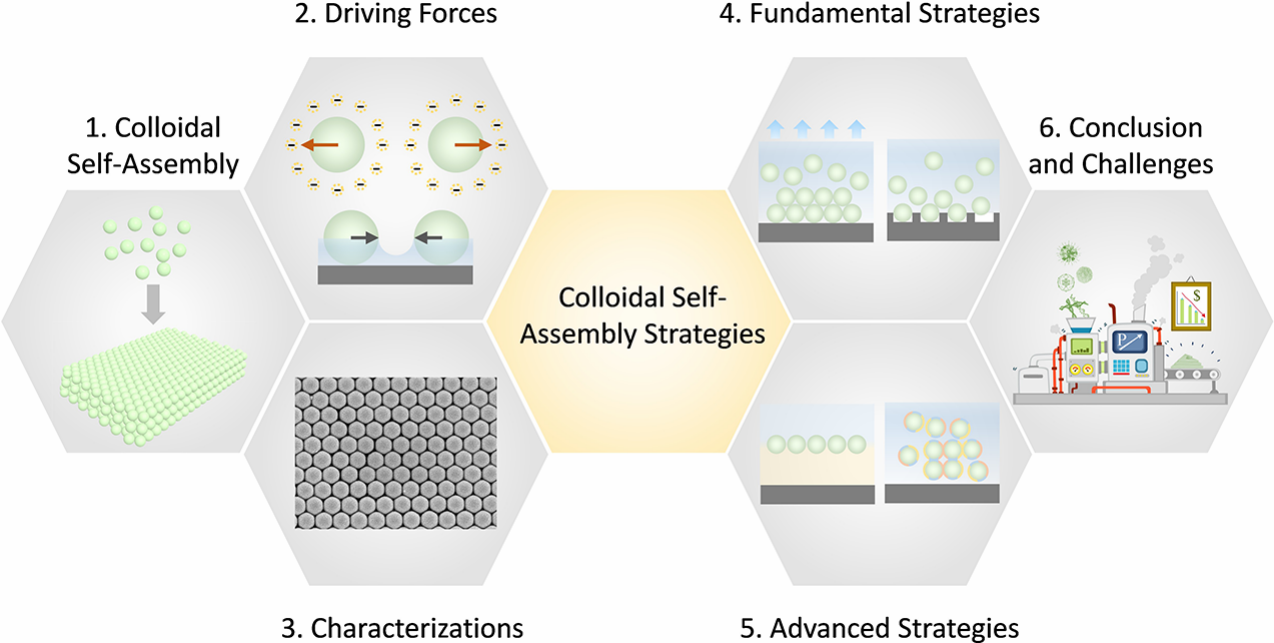
**Illustration of the review structure.** The review focuses on colloidal self-assembly strategies, which include an introduction (Section 1), and briefly introduces the driving forces (Section 2) as well as characterization approaches (Section 3). The review introduces colloidal self-assembly strategies, which are classified into Fundamental Strategies (Section 4) and Advanced Strategies (Section 5), depending on the programmability of strategies to the obtained assemblies. Finally, current challenges and perspectives toward improving colloidal self-assembly are discussed (Section 6).

**Table 1:** Main Content of Recent Reviews on Colloidal Self-Assemblies

Title of reviews	Topics of the review in left column
Nanoparticle Assembly and Oriented Attachment: Correlating Controlling Factors to the Resulting Structures^50^	1. Driving forces for oriented attachment processes.^50^
	2. Control factors of oriented attachment.^50^
	3. Structures induced by oriented attachment-induced 1D wires, 2D sheets, 3D branched structures, multiply twinned structures, necking, and defects.^50^
	4. Interparticle energies of the oriented attachment.^50^
Colloidal Self-Assembly Approaches to Smart Nanostructured Materials^41^	1. Engineering of the smartness of colloidal self-assemblies by external stimuli.^41^
	2. Regulation of the order of secondary structures by external stimuli.^41^
	3. Control of assembly orientation by external stimuli.^41^
Bioinspired Colloidal Photonic Composites: Fabrications and Emerging Applications^15^	1. Particles and polymeric matrix for colloidal photonic composites.^15^
	2. Infusion and coassembly of colloidal photonic composites.^15^
	3. Applications: Colorful coatings, visual sensors, anticounterfeiting, and photoluminescence.^15^
Stimulus-Responsive Photonic Crystals for Advanced Security^25^	1. Infiltration and responsive building blocks for stimulus-responsive nanostructures.^25^
	2. Optical encryption and information security based on mechano-responsive, solvent-controlled, pH-controlled, vapor-dependent anticounterfeiting, thermos-responsive, magnetic-/electric-field responsive colloidal crystals.^25^
Bottom-Up Assembled Photonic Crystals for Structure-Enabled Label-Free Sensing^24^	Sensors based on refractive index variation, lattice spacing variation, fluorescence, SERS, and configuration transition.^24^
Nanocrystal Assemblies: Current Advances and Open Problems^38^	1. Nanocrystal shapes and habits.^38^
	2. Nanocrystal assembly by controlling nanocrystal kinetics, solvent conditions, interfaces, electrophoretic deposition, 3D printing, and bioinspired protocols.^38^
	3. Models for the prediction of nanocrystal-based superstructures.^38^
	4. Dynamics, functions, and applications of superstructure.^38^
Stimuli-responsive self-assembly of nanoparticles^46^	1. Synthetic system under various chemical stimuli, including solvent, pH, gases, metal ions, and biomolecules.^46^
	2. Synthetic system under various physical stimuli, including temperature, magnetic, electric, and light.^46^
From dynamic self-assembly to networked chemical systems^47^	1. Equilibrium self-assembly.^47^
	2. Dynamic self-assembly induced by an external field.^47^
	3. Functions of dynamic self-assembly.^47^
Templated Colloidal Self-Assembly for Lattice Plasmon Engineering^45^	1. Self-assembly of meta-surface confined by templates.^45^
	2. Optical properties of plasmonic superlattices.^45^
	3. Applications of plasmonic superlattices, including SERS, perovskite nanocrystals.^45^
Advances in Colloidal Assembly: The Design of Structure and Hierarchy in Two and Three Dimensions^42^	1. Assembly of 2D nanostructures, including close-packed and nonclose-packed monolayers, binary monolayers, and patterned monolayers.^42^
	2. Assembly of 3D nanostructures, including 3D structures on planar interfaces, patterned 3D structures, colloidal epitaxy, and nonclose-packed 3D colloidal structures.^42^
Colloidal Self-Assembly: From Passive to Active Systems^54^	1. Equilibrium self-assembly of colloidal molecules, chains, and lattices.^54^
	2. Out-of-equilibrium self-assembly, including external field-induced colloidal self-assembly, hard template-confined self-assembly, anisotropic colloidal self-assembly, and ligand-dominated colloidal self-assembly.^54^
A Review on Self-Assembly of Colloidal Nanoparticles into Clusters, Patterns, and Films: Emerging Synthesis Techniques and Applications^55^	1. Strategies for constructing colloidal clusters, patterns, including emulsion directed self-assembly, magnetic field directed self-assembly, DNA origami, lithography-assisted self-assembly, and light-induced assembly.^55^
	2. Strategies for constructing colloidal films, including interfacial self-assembly, evaporative self-assembly, and functionalized substrates.^55^
	3. Applications of self-assembled structures, including photothermal effect, catalysis, photovoltaics, sensing, drug delivery, micro/nano reactors, and nanomachines.^55^
Co-assembly of nanometer- and submicrometer-sized colloidal particles into multicomponent ordered superstructures^52^	1. Development of building blocks, including uniformity, stability, and interparticle forces.^52^
	2. Kinetics, defects, and characterization of ordered structures.^52^
	3. Co-assembly of multicomponent structures, including coassembly via solvent evaporation, interface, electrostatic, DNA, external force, and emulsion.^52^

## 2 Driving Forces for Colloidal Self-Assembly

Self-assembly is a thermodynamic process that organizes building blocks into secondary structures. The resultant structures achieve equilibrium by minimizing the system’s free energies.^1^ A balance among three forces maintains the equilibrium: Internal attractive forces and repulsive forces between the colloidal nanoparticles as well as the external directional forces.^41,50,51,56^ The balance is critical for the assembling process as it determines the interparticle connectivity of the resultant structures. Any disruption in the balance affects the packing of obtained self-assemblies, such as generating defects or cracks in the final objects. In a self-assembling system, various forces synergistically dominate the packing of colloidal building blocks. Integration of these driving forces is critical for preparing various self-assemblies. The design of colloidal strategies is significant for synergistically using these driving forces. In this section, we briefly introduce some dominant forces used to direct colloidal self-assembly to help readers understand the colloidal self-assembly strategies in Sections 4 and 5.

### 2.1 Internal Forces

Internal forces refer to the short-range forces between building blocks, including attractive and repulsive forces. Attractive forces facilitate the aggregation of nanoparticles and maintain the structures. Repulsive forces prevent nanoparticles from forming random clumps. One significant attractive force is the van der Waals force, a short-range (<10 nm) force originating from the electromagnetic fluctuations due to the incessant movements of positive and negative charges within all types of atoms, molecules, and bulk materials. The van der Waals force potential between two spherical nanoparticles can be computed according to the following formula:^41^

$$V_{vdW}=-\frac{A}{6}\left(\frac{2}{R^{2}-4}+\frac{2}{R^{2}}+\ln\frac{R^{2}-4}{R^{2}}\right)$$

where *A* is the Hamaker constant between particles,^57^ and *R* is the particle center-to-center distance *r* normalized by the particle radius *a*. The strength of the van der Waals force between colloids can be estimated by summing all the van der Waals forces between the colloidal building blocks and their attached capping ligands. The van der Waals interaction weakens with increasing interparticle distance and particle size; thus, it can be modulated by varying the length of capping ligands, which in turn regulates the final assembled structure. Driven by this force, colloidal building blocks can form closely packed assemblies.^58,59^ During the packing process, ordering is often achieved by gradually increasing the NP volume fraction (e.g., by solvent evaporation) until reaching a solubility threshold. In addition, the van der Waals force is related to the shape of colloidal particles; thus, it has been used to induce oriented colloidal self-assembly of anisotropic particles.^60,61^

Depletion force is another attractive force between colloidal building blocks, which can be generated when mixing a suspension of large colloids with a dilute solution of small solutes.^62–64^ The small solutes act as depletants, such as polymers, small nanoparticles, micelles, salts, and others. Here, we use an example of a colloidal system containing two nanoparticles of different sizes with a specific volume overlap. The depletion force and potential in such a colloidal system can be estimated by the following equations:^65^

$$\begin{array}{l} F=-\pi n_{b}k_{B}TR_{d}^{2}\left[1-{\left(\frac{r}{2R_{d}}\right)}^{2}\right]\\ V=-\frac{4}{3}\pi n_{b}k_{B}TR_{d}^{3}\left[1-\frac{3r}{4R_{d}}+\frac{1}{16}{\left(\frac{r}{R_{d}}\right)}^{3}\right] \end{array}$$

where *r* is the center-to-center distance of the large colloids, *R*_*d*_ is the sum of the large colloid radius (*R*) and the excluded volume thickness ( σ2 ). The force and potential decay with the interparticle separation (*r*) in the range of [2*R*,2*R*_*d*_] and vanish when the separation is larger than 2*R*_*d*_.^41^ From the perspective of entropy, the aggregation of large colloids can increase accessible free space and translational freedom for depletants, increasing the system entropy. The depletion interaction intensifies with both the size and concentration of the depletants. The interaction becomes stronger when spherical particles are in solutions containing highly asymmetric solutes, or when they interact with planar or concave surfaces. Spherical particles adsorb strongly into concave wells patterned on two-dimensional substrates in the presence of smaller polymer solutes. The depletion interactions mediating this templated self-assembly are an order of magnitude larger than those between the spherical particles themselves.^66^

Besides, interactions between the capping ligands on the colloidal building blocks are also significant for colloidal self-assembly. The capping ligands expand the range of particle–solvent and interparticle interactions. In addition to van der Waals forces, capping ligands can bond with each other. One example is the hydrogen bond that forms between a hydrogen atom and a highly electronegative atom.^67,68^ One way to introduce hydrogen bonding is to modify colloidal building blocks with functional groups such as −OH, −COOH, or −NH_2_. The formation and strength of hydrogen bonds are highly dependent on solvent conditions. Generally, the free energy of hydrogen bonds is significantly weak in protic solvents. The strength of hydrogen bonds can also be regulated by the pH value of the system, further programming the self-assembly of colloids.^69^ Another example is the DNA base-pair interaction between the four nucleobases: Adenine (A), guanine (G), cytosine (C), and thymine (T). These interactions are specific in a temperature-dependent manner due to the corresponding base pairing (A-T and G-C). The attractive force can be precisely tuned by varying the temperature around the DNA melting point.^70,71^ Additionally, parameters, such as ionic strength and strands length offer further control, making DNA-mediated interactions highly versatile for assembling nanoscale components.

In addition to the bonding, electrostatic forces are ubiquitous for particles capped with ligands, as many functional groups hydrolyze in water, creating charges on the building blocks. The electrostatic interaction involves attraction and repulsion between oppositely charged and like-charged building blocks, respectively. The electrostatic potential energy can be described as the following equation:^72–74^

$$V_{elec}=2\pi \varepsilon_{r}\varepsilon_{0}a\psi_{0}^{2}\left(T\right)\ln\left(1+\exp\left(-ak\left(R-2\right)\right)\right)$$

where *a* and ψ_0_ are the radius of the particle and the surface potential of the particle, respectively, and *R* is the particle center-to-center distance *r* normalized by the particle radius *a*.^41^ The strength and range of electrostatic interactions can be controlled by adjusting the solvent (e.g., dielectric constant) and the properties of counterions (e.g., size and valence). Generally, like-charged objects always repel each other. However, recent research highlights the solvent’s critical role in modulating interactions between charged particles in solution. Remarkably, solvents can disrupt the charge-reversal symmetry:^75^ Negatively charged particles attract other negatively charged particles over long distances, while positively charged particles repel in an aqueous solution. Conversely, in alcohols, positively charged particles may attract each other, whereas negatively charged ones may repel. This phenomenon offers a powerful and promising route to program interparticle interactions and direct colloidal self-assembly.

### 2.2 External Forces

External forces are necessary to facilitate colloidal self-assembly when internal forces are too weak to pack colloidal building blocks over long distances. Gravity can aggregate colloidal building blocks, especially when it exceeds the buoyancy force of the colloids.^76,77^ However, gravity-dominated self-assembly is time-consuming and inefficient. Therefore, other forces are introduced. For example, the capillary force, resulting from differences in adhesive forces among liquid molecules and the forces at the liquid–solid and liquid–gas interfaces, plays a crucial role in manipulating interfacial colloidal self-assembly.^78–80^ In addition, centrifugal force,^81,82^ shear force,^83^ electric force,^84^ and magnetic force^85^ are usually applied to dominate colloidal self-assembly. When the external forces between nanoparticles balance with the internal forces, nanoparticles form well-assembled structures. The resulting structures are controllable through the strength and uniformity of the applied forces.

## 3 Characterization of Colloidal Self-Assembly

Driven by the internal and external forces, colloidal building blocks are assembled into secondary structures. Characteristics of colloidal self-assemblies, including morphology, packing lattice, composition distribution, and optical properties, are important to confirm their structure and function. Understanding these features is key to gaining insights into the dynamic self-assembly process, inferring the interparticle interactions, and designing applications for colloidal self-assemblies. In this section, we will review several common characterization methods to help readers understand the colloidal self-assembly techniques discussed in Sections 4 and 5.

### 3.1 Morphology

During self-assembly, colloidal building blocks spontaneously pack into specific morphologies and structures governed by particle interactions, shapes, and driving forces. These resultant structural features directly dictate the optical, mechanical, and surface properties of the assembled entities. Therefore, understanding the formation of morphologies is crucial, as it provides insight into both the dynamic assembly process and the final properties and applications of the materials. Microscopes, including optical, electronic, and scanning probe microscopes, are commonly used to directly observe the morphology of colloidal self-assembled entities because they can magnify small objects to visualize details. In this way, the details of the self-assemblies, including dimensions, shapes, and periodicity, can be revealed.^86–89^ Resolution is significant for a microscope since it determines the smallest structure that we can observe. Generally, the resolution of an optical microscope depends on both the quality of the lens and the wavelength of the incident light. To improve resolution, confocal microscopy is developed, where fluorescent samples are illuminated by a focused point of light from a pinhole. The emitted fluorescent signals from the in-focus point can pass through the pinhole to reach the detector, while signals from out-of-focus points are blocked by the pinhole. This method enhances the resolution. With the help of these optical microscopes, the morphology and dynamic processes of a self-assembly system can be revealed.^90–92^ A graphene-like colloidal assembly is monitored in situ,^93^ as shown in Figure 2a-i. Most particles (∼2 μm in size) organize into hexagonal rings, with a lattice spacing of several micrometers. The assembly dynamics are directly visualized using real-time optical microscopy. However, due to resolution limits, the optical microscope is suitable only for samples on the micron scale.

**Figure 2:**
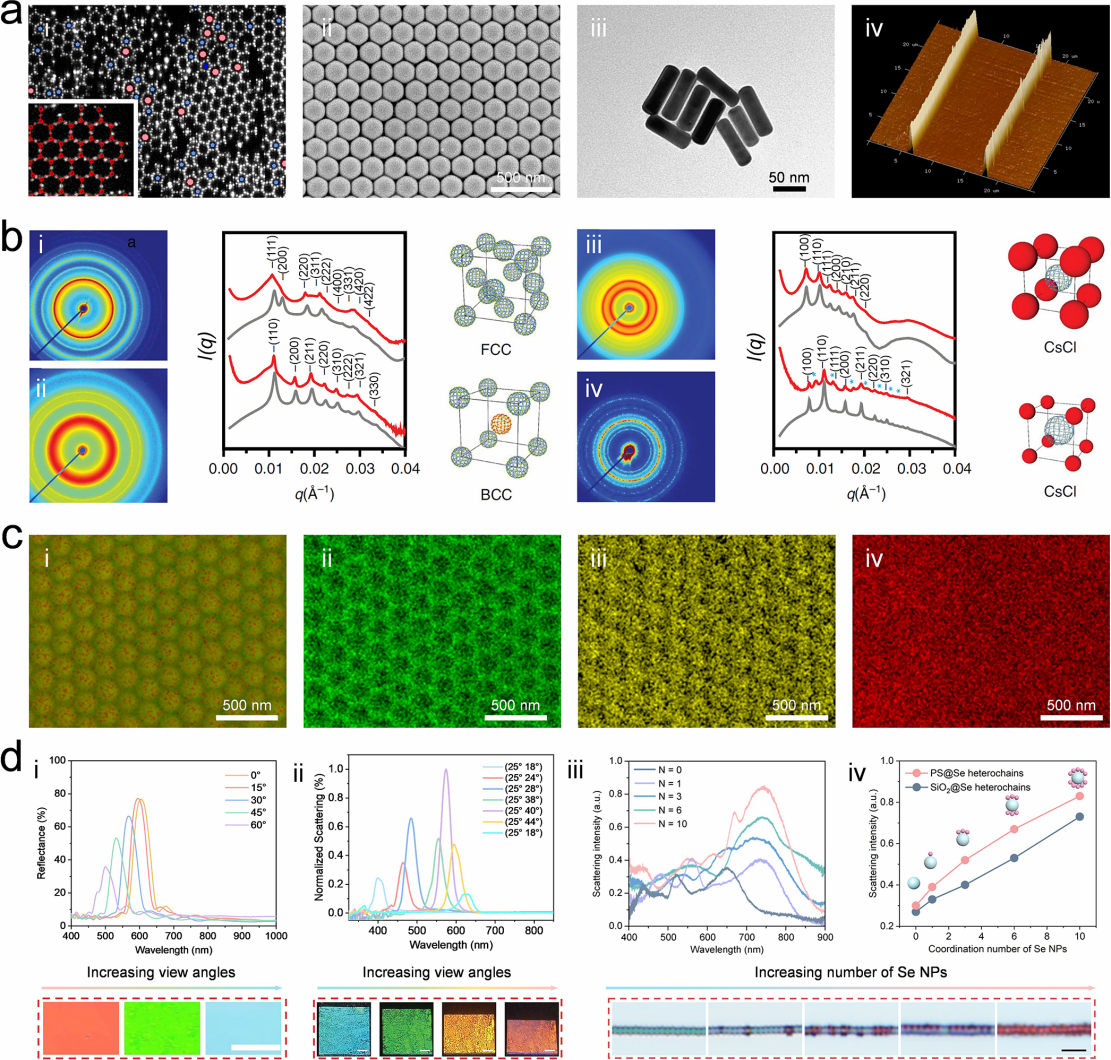
**Characterization of colloidal self-assembly.** a) Characterization approaches of colloidal self-assembly or building blocks, including confocal microscope (honeycomb lattice of pseudotrivalent particles, (i), scanning electron microscope (SEM, 180 nm silica nanoparticles packing into hexagon structure, (ii), transmission electron microscope (TEM, Au nanorods, (iii), and atomic force microscope (AFM, nanowires on a silicon wafer, (iv). Image i: Reproduced with permission from ref 93. Copyright 2023 Springer Nature under CC BY 4.0 (http://creativecommons.org/licenses/by/4.0/). Image ii-iv: Reproduced with permission from authors. Copyright authors. b) Packing lattice characterization of colloidal self-assembly. Images i-iv show 2D SAXS patterns for superlattices formed from 37 nm UiO-66 NPs in a face-centered cubic (FCC) arrangement, binary 37 nm UiO-66 NPs in a bcc arrangement, CsCl lattices formed from 37 nm UiO-66 NPs and 20 nm Au NPs (1:1 ratio), and 37 nm UiO-66 NPs and 40 nm AuNPs (1:1 ratio). Image i-iv: Reproduced with permission from ref 112. Copyright 2020 Springer Nature under CC BY 4.0 (http://creativecommons.org/licenses/by/4.0/). c) Composition characterization strategy of colloidal self-assembly. Images i-iv show element mapping by energy-dispersive X-ray spectroscopy of a colloidal composite made of silica NPs and PEG. Image i-iv: Reproduced with permission form authors. Copyright authors. d) Optical characterization of various colloidal self-assemblies. i: Reflectance spectrum for 3D colloidal crystals made of 330 nm silica nanoparticles and PEGDA; ii: Diffractive spectrum for monolayer nanosphere array made of 600 nm nanoparticles; iii-iv: Scattering spectrum for nanochains made of PS@Se and SiO_2_@Se. Reproduced with permission from authors and refs 121, 123, 125 . Copyright 2021 American Chemical Society. Copyright 2018 WILEY-VCH. Copyright 2024 American Chemical Society.

Electron microscopes, including scanning electron microscopes (SEM) and transmission electron microscopes (TEM), are designed to provide detailed insights into the structure of colloidal self-assemblies. Instead of using light, these microscopes utilize high-speed electrons energized in a vacuum to illuminate samples, thus, pretreatment of the conduction of samples is necessary. The interaction between the electron beam and the sample produces various signals, offering diverse ex-situ information. Typical SEM and TEM images are shown in Figures 2a-ii and iii, respectively. SEM images reveal coassembly of SiO_2_ nanoparticles (∼180 nm diameter) and PEG arranged into hexagonal accumulation on the (111) plane (e.g., assembly on a PDMS substrate, Figure 2a-ii), enabling calculation of lattice constant (∼165 nm) and photonic bandgaps (∼480 nm at vertical incidence). In contrast, TEM produces a two-dimensional projection of a sample, making it particularly suitable for revealing morphological features of core–shell particles,^94,95^ nanorods (e.g., Au nanorod on carbon, the aspect ratio is around 5:1. Figure 2a-iii), and particles with complex shapes.^96^ TEM generally offers higher spatial resolution than SEM and is therefore widely used for imaging small and thin specimens. Scanning probe microscopy is another effective ex-situ tool for examining three-dimensional topographic, physical, and chemical properties of colloidal self-assemblies. A common example is the atomic force microscope (AFM), which detects surface morphology by monitoring the deflection of the cantilever with a tip as it scans the sample.^97^ Unlike SEM, AFM does not require pretreatment of samples to make them conductive. It directly reveals the 3D surface morphology of colloidal assemblies, including periodicity, height, and feature shape (e.g., nanowires assembled on a wafer, Figure 2a-iv), from which the roughness can further be estimated. AFM is also suitable for characterizing soft materials, even in air or liquid environments. However, AFM can only scan a limited area slowly, with risks of damaging soft samples.

Besides the morphological information that can be directly observed from the SEM/TEM/AFM images, advanced information can be processed based on the basic morphological information. One typical example is fractal dimension (*d*_*f*_), which is used to estimate fractal aggregates.^98–104^ A large *d*_*f*_ indicates a dense packing of a fractal structure. The fractal structure endows the structure with some special optical properties, such as the polarized static scattering, which is influenced by the long-range fractal correlations.^105^ The scattering intensity follows a decay with the wave vector, *I*(*q*) ∼ *q* ^–*d*_*f*_^, serving as a direct signature of the fractal dimension *d*_*f*_. This relationship makes static light scattering a primary tool for characterizing fractal structure. By analyzing the decay of scattering intensity with wave vector, one can measure *d*_*f*_ in situ. Dynamic light scattering is used to study the kinetic aggregation.^103^ Analyzing the decay of the intensity autocorrelation function provides the aggregation rate and the hydrodynamic radius. For large aggregates, rotational diffusion must be considered. Direct imaging techniques, particularly confocal microscopy, provide real-space visualization.^104^ Subsequent image analysis (e.g., box-counting) validates *d*_*f*_ and directly reveals the evolution from clusters to a percolating network. Similar advanced information also involves packing lattice, periodicity, and optical properties, which will be introduced in the following sections.

### 3.2 Packing Lattice

In self-assembling, colloidal building blocks arrange into a specific packing lattice. The packing state of these building blocks results from the dynamic interaction of colloids and directly affects the properties of obtained entities, such as optical properties^106^ and mechanical properties.^30^ Thus, revealing the packing state of colloidal building blocks within a self-assembled entity is significant for understanding crystal epitaxy, solid physics, and applications. The packing lattice can be estimated by the optical microscope, SEM, and TEM images, referring to the arrangement of colloidal building blocks.^107^ X-ray diffraction (XRD) is widely used to analyze the crystal lattice by examining the interference pattern of X-rays scattered by the electrons within the crystal.^108,109^ However, due to the short wavelength of X-rays, XRD is not suitable for characterizing lattice of colloidal self-assemblies, whose constituent building blocks are orders of magnitude larger than atomic scales. To address this issue, a small-angle X-ray scattering system (SAXS) has been developed.^109–111^ SAXS employs scattering angles from 0.1° to 10°, enabling it to analyze materials with large motifs. The orientation of anisotropic nanoscale structures can be elucidated by 2D SAXS patterns. Depending on the degree of alignment, the SAXS pattern shows concentric circles or off-center spots corresponding to Bragg reflections from lattice planes (Figure 2b).^112^ Beyond structural orientation, SAXS is also suitable for shape and size analysis of the building blocks, and following the dynamic assembly process, with a substantially better statistical significance than TEM. However, SAXS is suitable for samples containing particles or structural features within the size range of tens of nanometers. For samples with larger particle sizes, ultrasmall-angle X-ray scattering (USAXS) is required, which uses smaller scattering angles than SAXS. USAXS can analyze samples made of colloidal building blocks with hundreds of nanometers.

### 3.3 Composition Distribution

In addition to the information on morphology and the packing state of building blocks, understanding the spatial distribution of elements in self-assembled structures is also essential for studying the assembly process and exploring applications in materials science. Energy-dispersive X-ray spectroscopy (EDS) is an effective method for mapping element distribution in colloidal self-assemblies.^86,113^ Similar to electron microscopes, EDS reveals the element distribution by scanning an electron beam across the sample surface. Figure 2c displays element distribution maps of silicon (ii), carbon (iii), and oxygen (iv) mapped by EDS, illustrating the distribution of different elements and compositions.

### 3.4 Optical Properties

Features of the colloidal self-assemblies can be derived from the materials used in the self-assembly system, such as the magnetic and catalytic properties.^46,55^ Except for the material-endowed properties, colloidal self-assemblies usually display optical characteristics because of their periodic arrangement of building blocks at a subwavelength scale.^114–116^ Optical spectra (reflectance spectrum,^117^ scattering spectrum,^118^ transmittance spectrum,^119^ and absorbance spectrum^120^) are crucial for characterizing the optical properties of colloidal entities, as they provide information about color, photonic bandgap, and structural periodicity. Figure 2d-i shows an angle-dependent Bragg’s reflectance of a 3D colloidal crystal made by 330 nm silica and PEGDA.^121^ With increasing viewing angles, the photonic bandgap blue-shifts gradually, following Bragg’s law:

$$\lambda =2\times D\times \sqrt{\Sigma {\left(n_{i}\times V_{i}\right)}^{2}-\sin^{2}\theta }$$

where λ is the diffraction wavelength, D is the lattice constant of the periodical structure, θ is the incident angle of light, *n*_*i*_ and *V*_*i*_ are the refractive indices and the volume fractions of each component of the ordered structure, respectively. The blue-shifted reflectance peaks with increasing incident angle indicate that colloidal nanoparticles pack into a multilayer periodic structure. The periodicity can be calculated according to Bragg’s law. In addition, the intensity and full width at half-maximum of the reflectance peak allow us to assess the orderliness and quality of the colloidal structure: A strong reflection intensity and a narrow full width at half-maximum indicate a high orderliness of self-assemblies.

The scattering spectrum constitutes a significant method for optical characterization. Unlike the reflectance spectrum, scattering spectra are obtained from structures such as gratings and two-dimensional colloidal crystals (monolayer nanosphere array).^122–124^ When a beam of light incident on these structures, diffraction occurs, which can be described by the equation:

$$\sin\theta_{in}+\sin\theta_{di}=\frac{2\lambda }{\sqrt{3}n_{c}D}$$

where λ is the wavelength of the diffracted light, *θ*_in_ is the incident angle, *θ*_di_ is the diffraction angle, and *n*_*c*_ is the system refractive index. The *D* represents the closest neighbor particle spacing within the plane. Diffraction peaks red-shift with increasing angle of incidence, distinguishing them from Bragg’s reflectance (e.g., monolayer colloidal array made of 600 nm PS nanoparticles, Figure 2d-ii).^123^ The feature provides clear evidence for the formation of grating structures or two-dimensional colloidal crystals. Another structure suitable for scattering characterization is nanochains. The scattering peak position and intensity of nanochains are sensitive to their local environments, including bound biomacromolecules or the coordination number of the nanoparticles around the nanochains (e.g., measured scattering spectra of PS@Se heterochains with different number of Se NPs (Figure 2d-iii), scattering intensity of PS@Se heterochains and SiO_2_@Se heterochains at their resonance wavelengths of 727 and 679 nm (Figure 2iv)).^125^ Corresponding shifts in peak location and intensity produce visible color changes, which directly indicate environmental alterations. This makes nanochains effective as visual sensors for applications, such as virus detection.^126^

### 3.5 Summary

The characterization of colloidal self-assemblies is of paramount importance, as it offers valuable insights into the nature of the self-assembled entities through both qualitative and quantitative methodologies. Such information not only supports the practical application of these entities but also advances our comprehension of the underlying scientific principles. A variety of tools have been developed to elucidate this information. Here, we introduce some measurements for colloidal self-assemblies. Other tools also exist, such as Zeta potential measurements to confirm the charges on the building blocks,^95^ scanning transmission electron microscopy for confirming the packing lattice,^127^ dynamic light-scattering for measuring the sizes of the building blocks and the stability of the self-assembled structure,^112,128^ Fourier transform infrared spectrometry for identifying the chemical bonds and functional groups in colloidal self-assembly,^129^ and circular dichroism spectroscopy for measuring the absorption of left and right circularly polarized light in a chiral structure.^130^ Besides, we focus on the primary applications of each characterization method mentioned in this section. For example, AFM can confirm surface tension and mechanical properties of ultrathin films.^131^ Notwithstanding significant advancements, new challenges emerge, encompassing the improvement of morphological characterization techniques, elucidation of interparticle interactions during self-assembly, and implementation of real-time monitoring of the dynamic behaviors of colloidal building blocks. A summary of general characterization approaches is provided in Table 2.

**Table 2:** Summary of General Characterization Approaches of Colloidal Self-Assemblies

Characterization strategies	Samples	Obtained information
Optical microscopy	Colloidal building blocks or assemblies in micron size or larger.	Dynamic assembling process or morphology of colloidal self-assembly.
Confocal microscope	Fluorescent colloidal building blocks or assemblies in micron size or larger.	Dynamic assembling process or morphology of colloidal self-assembly.
Scanning electron microscope	Colloidal building blocks, powders, and films of colloidal assemblies.	Morphology and packing lattices of colloidal building blocks and assemblies, element distribution.
Transmission electron microscope	Colloidal building blocks or thin films of colloidal assemblies.	Morphology and packing lattice of colloidal building blocks and assemblies, element distribution.
Atomic force microscope	Films of colloidal assemblies, colloidal suspensions.	Morphology of colloidal self-assemblies, interaction between colloidal building blocks, and Young modulus.
Energy-dispersive X-ray spectroscopy	Colloidal building blocks, powders, and films of colloidal assemblies.	Mapping of the element distribution in a colloidal self-assembly.
(Ultra) Small-angle X-ray scattering	Powders and films of colloidal self-assemblies.	Packing lattice and periodicity of colloidal assemblies, as well as shape and size analysis of the building blocks, and following the dynamic assembly process.
Dynamic light scattering	Colloidal suspension.	Size and zeta potential of colloidal building blocks, stability of colloidal self-assemblies, and dynamic assembly process.
UV–visible spectrometer	Films of colloidal self-assembly and colloidal suspension.	Optical properties, such as the absorbance and scattering of colloidal assemblies and suspensions.
Fourier transform infrared spectrometer	Powders and films of colloidal self-assemblies.	Chemical bond and functional groups in colloidal self-assemblies.
Angle-resolved spectrometer	Films of colloidal self-assemblies.	Angle-resolved optical properties of colloidal self-assemblies, including reflectance, scattering, and transmittance.
Thermal gravimetric analyzer	Powders or films of colloidal self-assemblies.	Thermal stability and composition of colloidal self-assemblies.
Circular dichroism	Films or powders with chiral structures.	Absorption of left and right circularly polarized light in a chiral structure.

## 4 Fundamental Colloidal Self-Assembly Strategies

Colloidal self-assemblies show promising potential in many applications, including displays,^113,132–134^ soft actuators,^118,135,136^ anticounterfeiting.^122,137^ Various colloidal self-assemblies are required to cater to specific application scenarios. For example, pixelated colloidal assemblies are required for display-related applications, whereas a colloidal film is necessary for biosensors or wearable devices. Efficient self-assembly of diverse structures is significant for these application scenarios. Thus, this review provides a comprehensive discussion on colloidal self-assembly strategies. In this section, we summarize the strategies for assembling colloidal crystal dots, lines, and films (Table 3). These strategies are classified as fundamental strategies as they depend on the evaporation of colloidal suspensions and lack programmability of the resulting structures.

**Table 3:** Fundamental Self-Assembly Strategies for Colloidal Crystal Dots, Lines, and Films

	Design Principles	Dominated driving forces	Obtained structures	Advantages	Disadvantages	References
Self-assembly of colloidal crystal dots	Droplet inner flows	Capillary and Marangoni flows	Homogeneous or heterogeneous colloidal crystal dots	Facile, high-resolution, customizable patterns	Require the diminishment of the coffee-ring effect	^148–151^
	Droplet three-phase contact line			Shape-programmable and patternable colloidal crystals	Require predesigned substrate or droplet component	^154–161^
	Evaporation-triggered phase separation			Facile crystal structure	Need control of the chosen component	^106^,^162–169^
	Microfluidics	Space confinement and van der Waals force	Homogeneous or heterogeneous colloidal microbeads	High-throughput, programmable bead size	Prepare spherical or hemispherical structures	^172–194^
	Surface-modified substrate	Interactions between the particles and the substrate	Dot array	Programmable plane orientations or hierarchical structures	Depend on the template made by the top-down method	^138^,^195–202^
Self-assembly of colloidal crystal lines	Colloidal droplet coalescence	Surface tension	Colloidal crystal lines	Customizable patterns	Need control of the footprints of the coalescent droplets	^140^,^141^,^202–210^
	Direct write printing	Shear force	Colloidal crystal lines	Customizable patterns	Need diminished Rayleigh instability	^211–218^
	Hard templates	Space confinement	Colloidal crystal lines and patterns, and colloidal crystals with special packing lattices	Programmable plane orientations or hierarchical structures	Depend on the template made by the top-down method	^219–227^
	Soft templates		Colloidal crystal lines and patterns, and colloidal crystals with special packing lattices		Require assistance from predesigned templates	^228–238^
Self-assembly of colloidal crystal films	Meniscus capillary force	Capillary force	Homogeneous or heterogeneous colloidal Film	High-quality, facile operation	Require a long time	^79^,^253–262^
	Shear force	Shear force	Homogeneous colloidal film	Scalable, efficient process	Cannot prepare patterns	^83^,^263^
	Other strategies	Electrostatic force, centrifugal force	Homogeneous colloidal films	Scalable, robust process	Depend on the equipment	^80^,^264–276^

### 4.1 Self-Assembly of Colloidal Crystal Dots

Colloidal crystal dots refer to the colloidal assemblies with characteristic dimensions below 1 cm, encompassing structures such as clusters, domes, and microbeads. Interest in colloidal crystal dots arises not only from their direct use in applications such as display pixels and anticounterfeiting but also from their role as building blocks for fabricating lines, films, and patterned architectures. A common fabrication method is the evaporation of colloidal droplets, as it offers considerable freedom to program the self-assembly process, such as internal droplet flows, contact-line behavior, droplet composition, and drying conditions. Alternatively, strategies employing surface-modified substrates or microfluidics are also effective. This section discusses the assembly strategies for colloidal crystal dots.

#### 4.1.1 Droplet Inner Flows

Droplet evaporation-induced colloidal self-assembly is commonly employed to prepare colloidal dots.^138–141^ Two inner flows occur in a drying sessile droplet:^142^ The first one is an outward capillary flow caused by a gradient decrease in evaporation flux from the droplet rim to its center, while the second is a Marangoni flow driven by a surface tension gradient along the droplet surface (Figure 3a).^143,144^ The two flows synergistically manipulate the distribution and assembly of the colloidal building blocks in a drying sessile droplet.^145^ When the outward capillary flow predominates the colloidal movement, colloidal nanoparticles migrate to the rim of the sessile droplet to compensate for the mass loss at the droplet edge due to the unshrinkable three-phase contact line (TCL).^146^ Consequently, a coffee-ring-like deposition with a thick rim and a thin center is produced (Figure 3b).^106^ However, a uniformly assembled colloidal crystal dot is often preferred over a coffee-ring stain. To mitigate the coffee-ring effect, Marangoni flow can be created in a sessile droplet to counteract the capillary flow (Figure 3c).^147,148^ Heating the substrate induces a thermo-driven Marangoni flow as the heated substrate creates a negative temperature gradient from the rim to the center of the droplet, along with a corresponding surface tension gradient. This results in an inward flow within the drying sessile droplet. The inward Marangoni flow opposes the outward capillary flow, leaving a uniform coating of the colloidal crystal. Another strategy involves adjusting the evaporation flux at the droplet surface to diminish the coffee-ring effect.^149^ In a drying colloidal droplet, a skin layer of nanoparticles forms when the evaporation is sufficiently fast, which is regulable with the drying temperature and humidity.^150^ The emergence of the skin layer governs the evaporation flux along the droplet surface, thereby influencing the flow direction within the droplet and morphologies of produced assemblies. (Figure 3d).^151^

**Figure 3:**
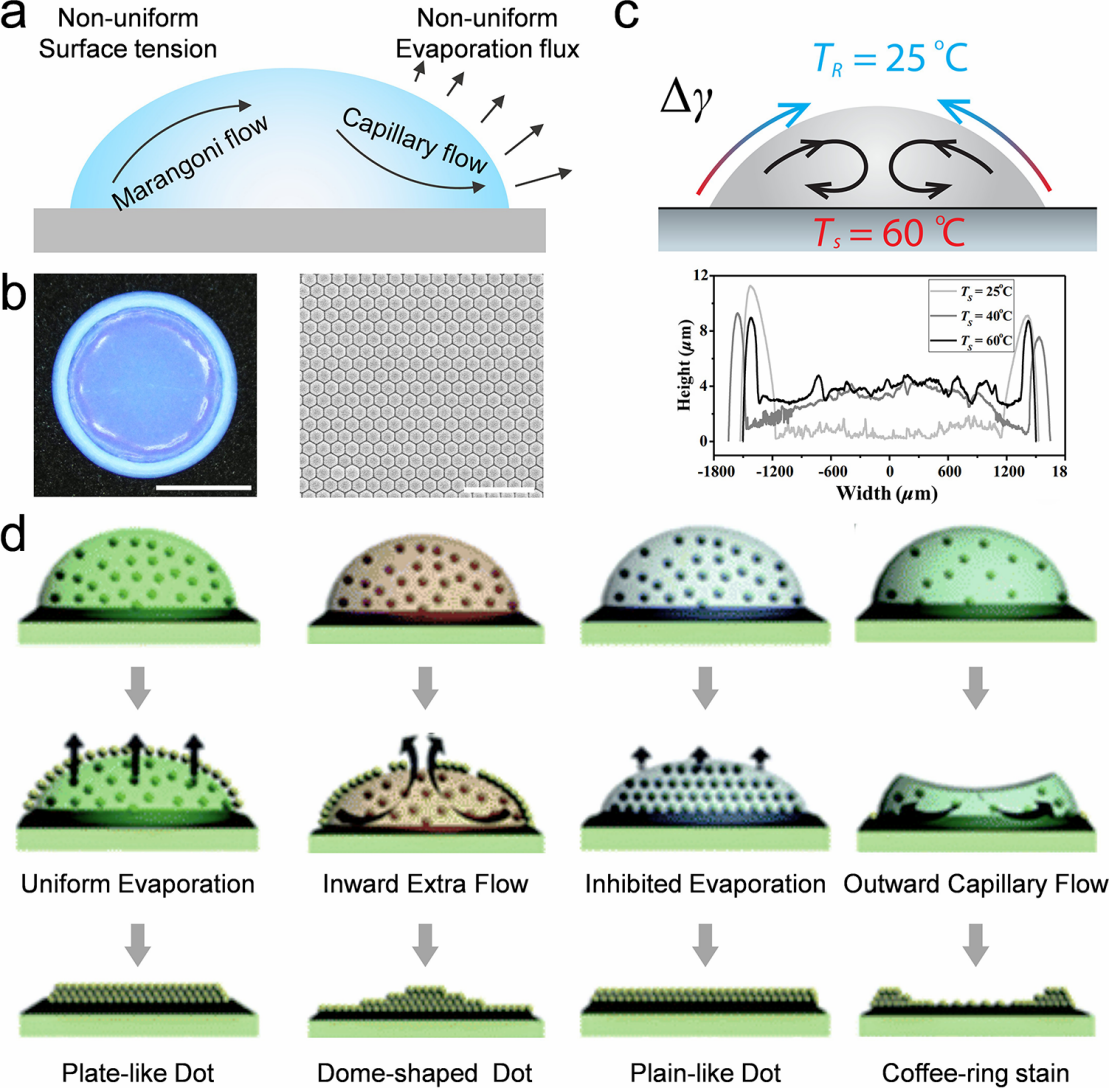
**Self-assembly of colloidal crystal dots by regulating droplet inner flows.** a) Schematic diagram of the flows in a drying sessile droplet. An outward capillary inner flow and an inward Marangoni inner flow occur due to the nonuniform evaporation rate and surface tension along the droplet surface. b) A coffee-ring-like deposition driven by the outward droplet inner flow in a drying sessile droplet. Reproduced with permission from ref 106. Copyright 2023 American Chemical Society. c) Uniform assembly of colloidal nanoparticles with the assistance of Marangoni flow. The thermo-induced Marangoni flow created by a heated substrate reverses the coffee-ring effect. Reproduced with permission from ref 148. Copyright 2018 American Physical Society. d) Colloidal assembly regulated by the formation of colloidal skin at the droplet surface. The evaporation flux along with the droplet surface is related to the density of the colloidal skin, thus influencing the direction and intensity of the droplet inner flow. Reproduced with permission from ref 149. Copyright 2019 Royal Society of Chemistry.

#### 4.1.2 Droplet Three-Phase Contact Line

Droplet TCL constitutes another critical parameter for controlling the dried deposition. The wettability of the substrate influences the TCL behavior. On a hydrophilic substrate, the droplet TCL gets pinned, resulting in a coffee-ring stain due to the outward flow in the droplet.^146^ However, when evaporating a colloidal droplet on a hydrophobic substrate where the droplet TCL cannot get pinned, the continuously sliding TCL suppresses the outward radial capillary flow, leading to a colloidal crystal dome or donut.^152,153^ The wettability-dependent TCL behavior allows for programmable shrinkage of TCL by setting pinning points on a substrate, such as hydrophilic patterns on a hydrophobic surface (Figure 4a).^154^ The droplet TCL adheres to the hydrophilic points. The resulting asymmetric dewetting on the hydrophobic region influences the morphology of the colloidal assembled deposition. Substrate rheology is another critical parameter to regulate the TCL behavior (Figure 4b). When a colloidal droplet is suspended on a liquid poly­(dimethylsiloxane) (PDMS) substrate, the droplet sinks into the liquid PDMS, forming a water-in-oil (W/O) structure, leaving a bump-shaped colloidal dot. When a colloidal droplet is dispensed on a viscoelastic PDMS substrate, the droplet deforms the surface. The local deformation at the droplet TCL reduces the coffee-ring effect, resulting in a plate-shaped colloidal dot. On a solid PDMS substrate, the TCL of the droplet remains pinned due to the adhesive substrate, forming a coffee-ring colloidal pattern dot.^155^ In addition to the substrate, the droplet component is also significant in tuning the droplet TCL behaviors. The solvent within a droplet influences TCL behaviors by affecting the surface tension, the droplet viscosity, and the impact dynamics of the droplet on substrates.^156,157^ For example, controlling the proportion of xylene and cyclohexane within a colloidal droplet enables the pinning or depinning of its TCL, resulting in the deposition of various shapes (Figure 4c).^158^ Colloidal concentration also programs the droplet TCL behavior, as the aggregation of nanoparticles near the droplet TCL causes the self-pinning of the TCL.^159^ A droplet with proper colloidal concentration retracts its TCL in a stick–slip mode, where the TCL alternatively gets pinned on the substrate and shrinks due to the competitive result of the pinning and depinning force acting on the TCL (Figure 4d).^160^ A ring is obtained when the TCL gets pinned. As the droplet volume decreases and the contact angle declines due to evaporation, an inward depinning force drags the TCL to shrink when the contact angle is smaller than a critical value. The shrinkage continues until the TCL gets pinned again. The stick–slip motion of the TCL leads to a multiring structure.^161^

**Figure 4:**
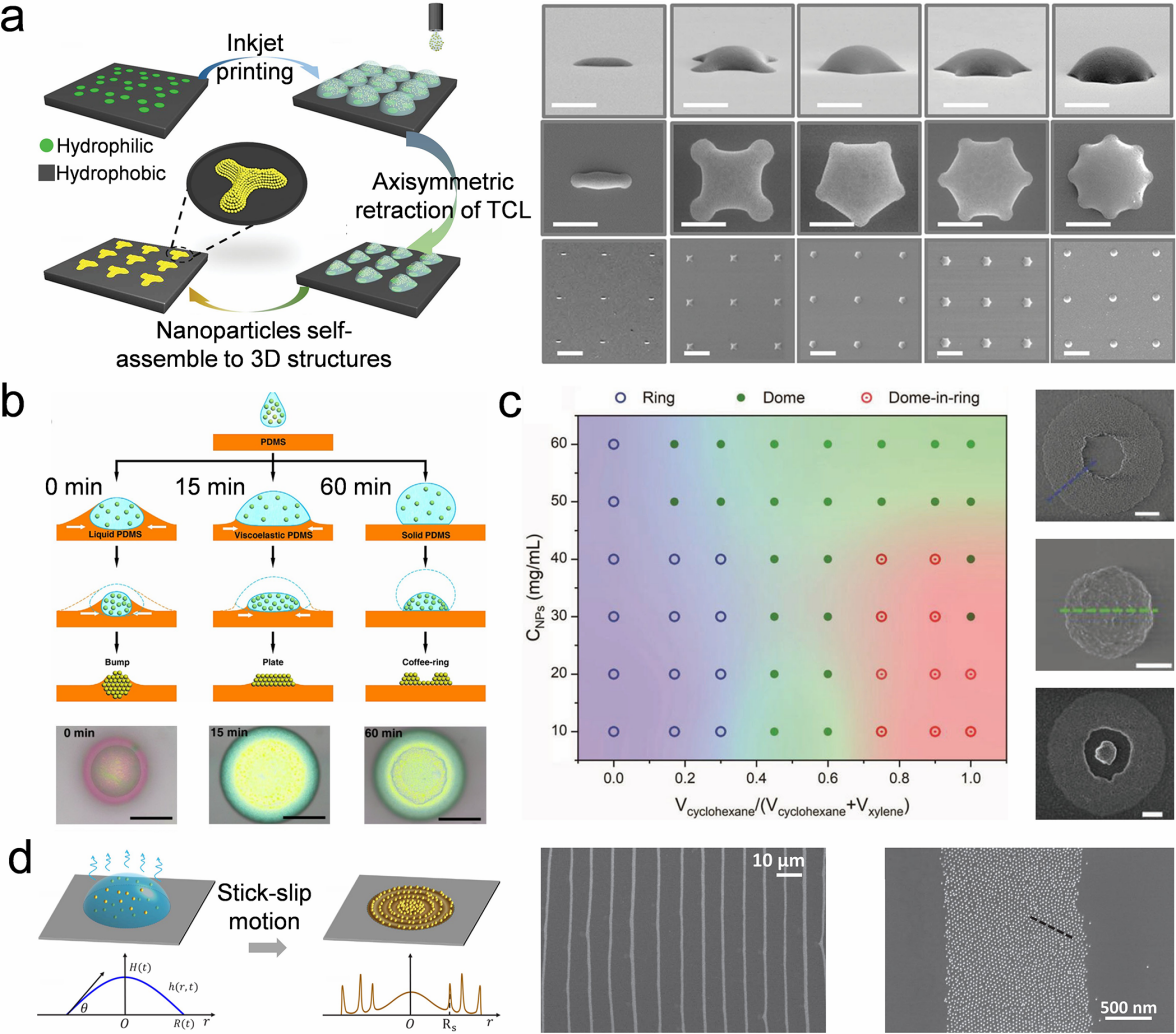
**Self-assembly of colloidal crystal dots by regulating droplet TCL.** a) Manipulating the 3D morphology of colloidal assembly by asymmetric dewetting of drying colloidal droplets. Hydrophilic points are constructed on a hydrophobic substrate. The droplet TCL gets pinned by the hydrophilic points, and shrinks at the hydrophobic zone, leading to colloidal assemblies with various 3D morphologies. Reproduced with permission from ref 154. Copyright 2015 Wiley-VCH. b) Various-shaped colloidal assemblies manipulated by substrate rheology. Reproduced with permission from ref 155. Copyright 2016 Wiley-VCH. c) Solvent composition-dependent colloidal self-assembly with different morphologies. The composition of the solvent affects the surface tension, viscosity, and bouncing behavior of the droplet, thus influencing the self-assembly of colloidal nanoparticles. Reproduced with permission from ref 158. Copyright 2023 Wiley-VCH. d) Multiring colloidal deposition due to the stick–slip motion of the droplet TCL over the drying process. Reproduced with permission from ref 160, 161 . Copyright 2018 American Chemical Society. Copyright 2020 Wiley-VCH.

#### 4.1.3 Evaporation-Triggered Phase Separation

Nonvolatile components, such as polymers, have been used to program the colloidal assembly in a drying droplet. On the one hand, polymers affect the surface tension and viscosity of the droplet, the droplet drying behaviors, and the dispersion of nanoparticles in the dried colloid–polymer deposition.^162^ On the other hand, phase separation of colloids and polymer might occur in a drying colloid–polymer droplet. When nanoparticles can strongly interact with polymers, gradual densification of nanoparticles occurs during evaporation (Figure 5a-i). However, when the nanoparticles have a poor affinity for the polymer, phase separation occurs during the drying process, randomly aggregating particles in the polymer matrix (Figure 5a-ii).^163^ The phase separation can also trigger the stratification of polymers and colloids, depending on the wettability of the polymer on the colloids.^164^ As the solvent evaporates, a dense polymer-rich skin phase forms at the air–liquid surface when the polymer concentration is high enough or the evaporation rate is fast enough. When the nanoparticles are strongly wettable by the polymer or the skin phase forms slowly enough, a nanoparticle-rich skin phase can be formed. Otherwise, nanoparticles mainly distribute in the bulk of the polymer film (Figures 5b-c).^165–168^

**Figure 5:**
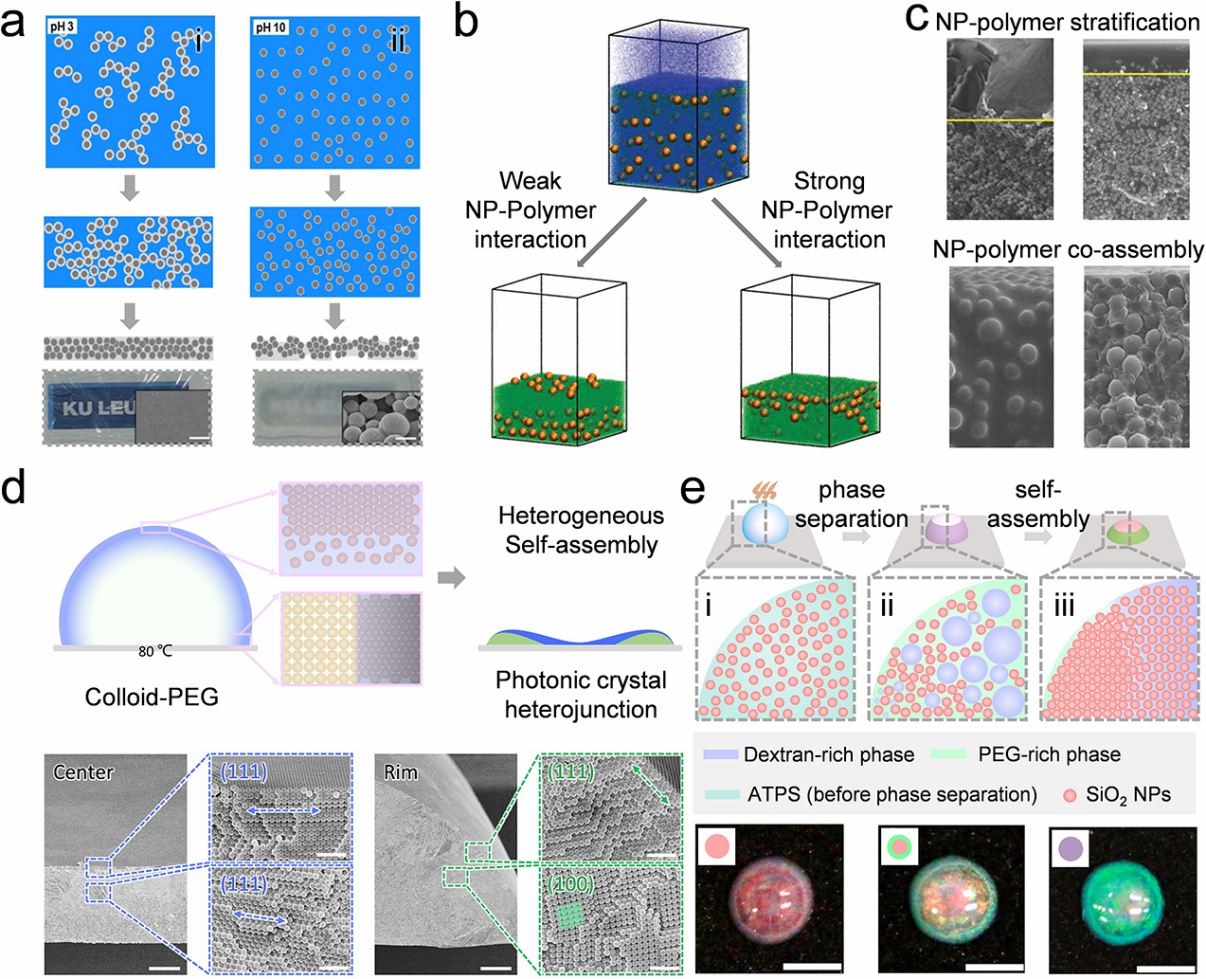
**Self-assembly of colloidal crystal dots by evaporation-triggered phase separation.** a) Schematic diagram of the structural evolution of silica nanoparticles during the drying of silica/PVA films at different pH values. Random colloidal aggregates and coassembled colloid-PVA film are obtained in the dried film due to the pH-dependent polymer–colloid interactions. Reproduced with permission from ref 163. Copyright 2016 American Chemical Society. b) Simulation of the nanoparticle distribution in different polymer solutions as the solvent evaporates. Weak polymer–nanoparticle interaction could induce self-stratification of nanoparticles and polymers in the dried composites. Reproduced with permission from ref 164. Copyright 2016 American Chemical Society. c) SEM images of dried films of polymer–colloid mixtures with stratification (upper line) and coassembly (bottom line) controlled by the formation speed of the skin phase. Reproduced with permission from ref 168. Copyright 2020 Royal Society of Chemistry. d) Heterogeneous self-assembly of monosized silica nanoparticles in a drying colloid-PEG droplet. Nanoparticles pack into the (111) plane orientation of an FCC lattice in the fast-descending skin phase. The (100) plane is formed at the droplet rim adjacent to the skin phase. Reproduced with permission from ref 106. Copyright 2023 American Chemical Society. e) Nonuniform self-assembly of the monosized silica nanoparticles with the assistance of the phase separation of a PEG-DEX-based aqueous two-phase system. Reproduced with permission from ref 169. Copyright 2022 American Chemical Society.

Besides, phase separation in a drying colloid–polymer droplet can induce heterogeneous self-assembly of a single type of nanoparticle. In a fast-drying colloid-poly­(ethylene glycol) (PEG) droplet, a skin phase forms across the droplet surface during the drying process. When the descending rate of the droplet surface is 3 times larger than the diffusion rate of the nanoparticles near the droplet surface, nanoparticles are trapped by the fast-descending skin phase and pack into the (111) plane orientation of a face-centered cubic (FCC) lattice because of the surface capture effect. The (100) plane is formed at the droplet rim adjacent to the skin phase due to the space-confinement effect (Figure 5d).^106^ The heterogeneous colloidal self-assembly is closely related to the polymer concentration, as it affects the droplet viscosity significantly, further determining the intensity of the droplet inner flow and the skin formation. The heterogeneous self-assembly in colloid-PEG droplets is universal for different colloidal particles with various droplet sizes, shapes, and diverse substrates. The phase separation can also be induced by two incompatible polymers, such as PEG and Dextran, and heterogeneously assemble colloids in the PEG-rich and DEX-rich phases. This is because the silica nanoparticles show different partition affinities to the two phases (Figure 5e).^169^ The drying temperature and the colloidal concentration can tune the obtained structure. Adding polymers provides an extra degree of freedom to program the self-assembly of colloidal nanoparticles in a drying droplet.

#### 4.1.4 Microfluidics

Besides drying in air, colloidal droplets can be dried in another immiscible liquid phase to generate colloidal microbeads. An example is illustrated by microfluidics, which integrates various fluids into one system through microchannels, chamber structures, pump valves, and other geometric structures.^170,171^ In a microfluidic system, colloidal droplets are injected into a continuous liquid phase that is immiscible with the colloidal suspension. Nanoparticles accumulate to form a homogeneously close-packed colloidal crystal bead with evaporating solvent (Figure 6a).^172,173^ The diameter of colloidal nanoparticles determines the lattice constant of the resulting microbeads, generating different structural colors (Figure 6b).^173^ Compared with the droplets dried in the air, colloidal self-assembly using microfluidics avoids TCL and droplet inner flow, thus diminishing their influence on the self-assembly process. Microfluidics can efficiently fabricate anisotropic colloidal assemblies,^174–182^ for instance, by mixing various building blocks in one droplet. During the drying process, different building blocks, such as graphene oxide (GO) and silica nanoparticles, separate from each other due to their different densities, forming hemispherical colloidal crystal clusters and oblate GO components (Figure 6c).^183^ In addition, structures with multicomponent can be achieved using microfluidics by mixing various nanoparticles within a double-emulsion droplet. These particles can organize into their own crystalline grains to minimize free energy driven by depletion forces and osmotic pressure. The resulting self-assemblies exhibit highly saturated composite structural colors and multiple reflectance peaks. By adjusting the particle mixing ratio and osmotic pressure, it is possible to produce microbeads with full colors (Figure 6d).^184^

**Figure 6:**
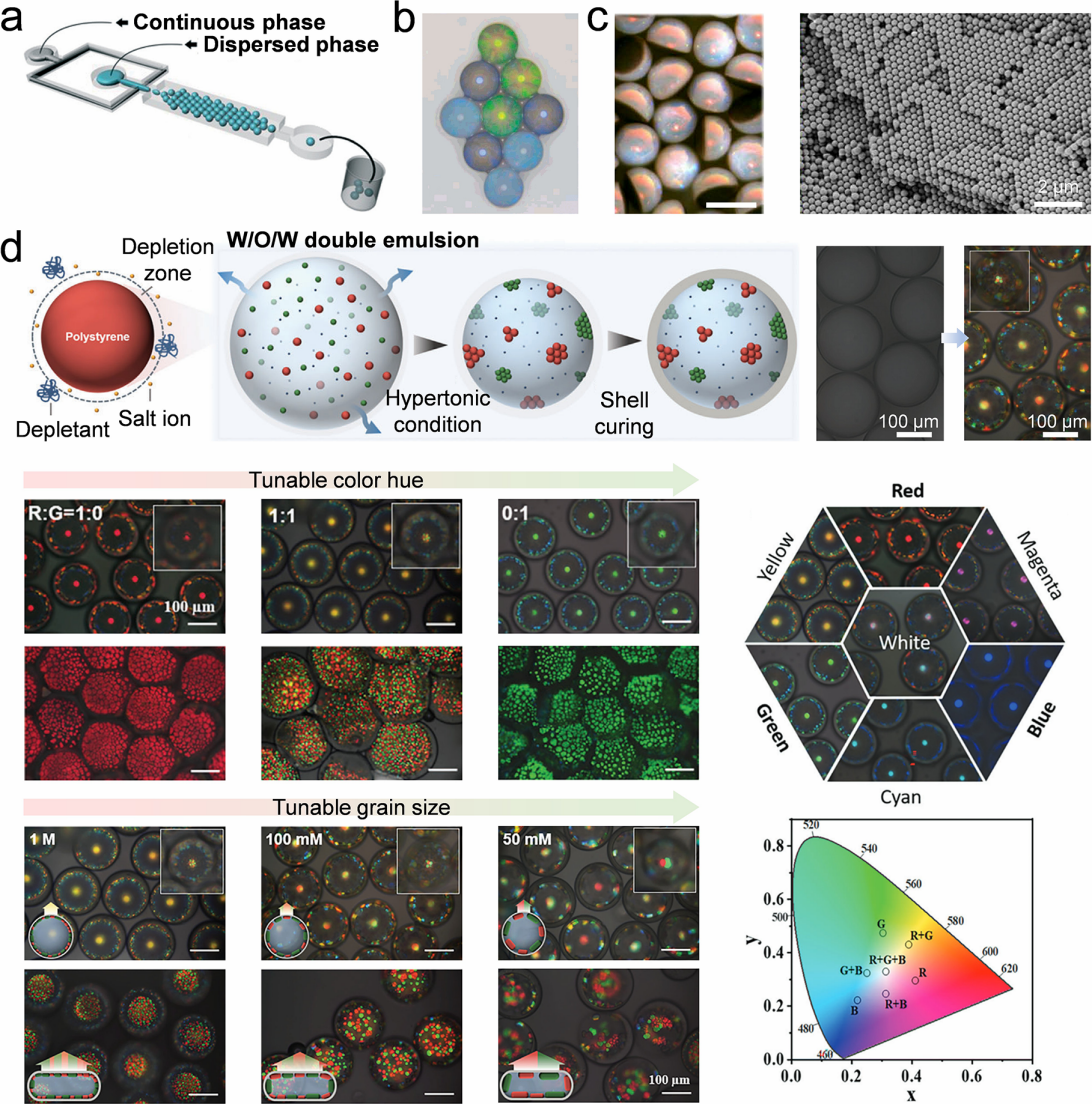
**Self-assembly of colloidal crystal microbeads by microfluidics.** a) Illustration of the fabrication of colloidal crystal microbeads by a microfluidic device. Colloidal droplets are injected into a continuous liquid phase that is immiscible with the colloidal suspension. With the evaporation, nanoparticles assemble into colloidal crystals. Reproduced with permission from ref 172. Copyright 2017 Royal Society of Chemistry. b) Digital photo of closely packed colloidal crystal beads obtained by microfluidics. The beads are made of colloidal nanoparticles with different diameters, showing diverse colors and reflectance peaks. Reproduced with permission from ref 173. Copyright 2008 American Chemical Society. c) Janus colloidal crystal beads made by a mixture of graphene oxide (GO) and silica nanoparticles. Because of the different densities, the GO and silica nanoparticles separate from each other to form Janus hemispherical beads. Reproduced with permission from ref 183. Copyright 2020 The American Association for the Advancement of Science. d) Different-sized colloidal nanoparticles can self-assemble into their own crystalline grains in a double emulsion droplet driven by the depletion force and osmotic pressure, generating microcapsules with binary or ternary colloidal mixtures. Reproduced with permission from ref 184. Copyright 2023 Wiley-VCH.

Even with adjustable morphology and particle distribution, colloidal nanoparticles can only form densely packed lattices when dispersed in water. Nonvolatile materials, such as curable resins or hydrogels,^185^ are mixed with nanoparticles to prepare diverse structures.^186^ By varying the ratio of the particle and curable polymers, nonclosely packed structures can be obtained (Figure 7a).^186^ Introducing nonvolatile materials enhances the variety of derivative structures of the composite beads. For instance, it becomes possible to create heterogeneous colloidal crystal beads comprising an inverse opal shell and an opal core by partially etching colloidal nanoparticles (Figure 7b). The heterogeneous colloidal crystal microbeads display two reflectance peaks and two colors (Figure 7c).^187,188^ Besides, inverse opals, polymer scaffolds can also be prepared in this way.^188–190^ Moreover, colloidal microbeads with various components have been designed using microfluidic devices (Figure 7d).^191,192^ The multiple components of the microbeads are achieved by incorporating four channels to inject various dispersed phases into a single device. The volume ratio of each component in the microbeads can be precisely controlled by adjusting the flow rates of the channels. Typically, the droplet size produced by microfluidics ranges from tens to hundreds of micrometers, depending on the flow rate of the dispersed phase. To enhance the programmability of the droplets, electrospray has been integrated into the microfluidic system. With the help of microfluidic electrospray, microcapsules with the desired colloidal crystal cores have been fabricated (Figure 7e).^178^ Such design broadens the diversity of artificial microstructures and endows such structures with unique properties such as multiple bandgaps, and multicolors.^193,194^

**Figure 7:**
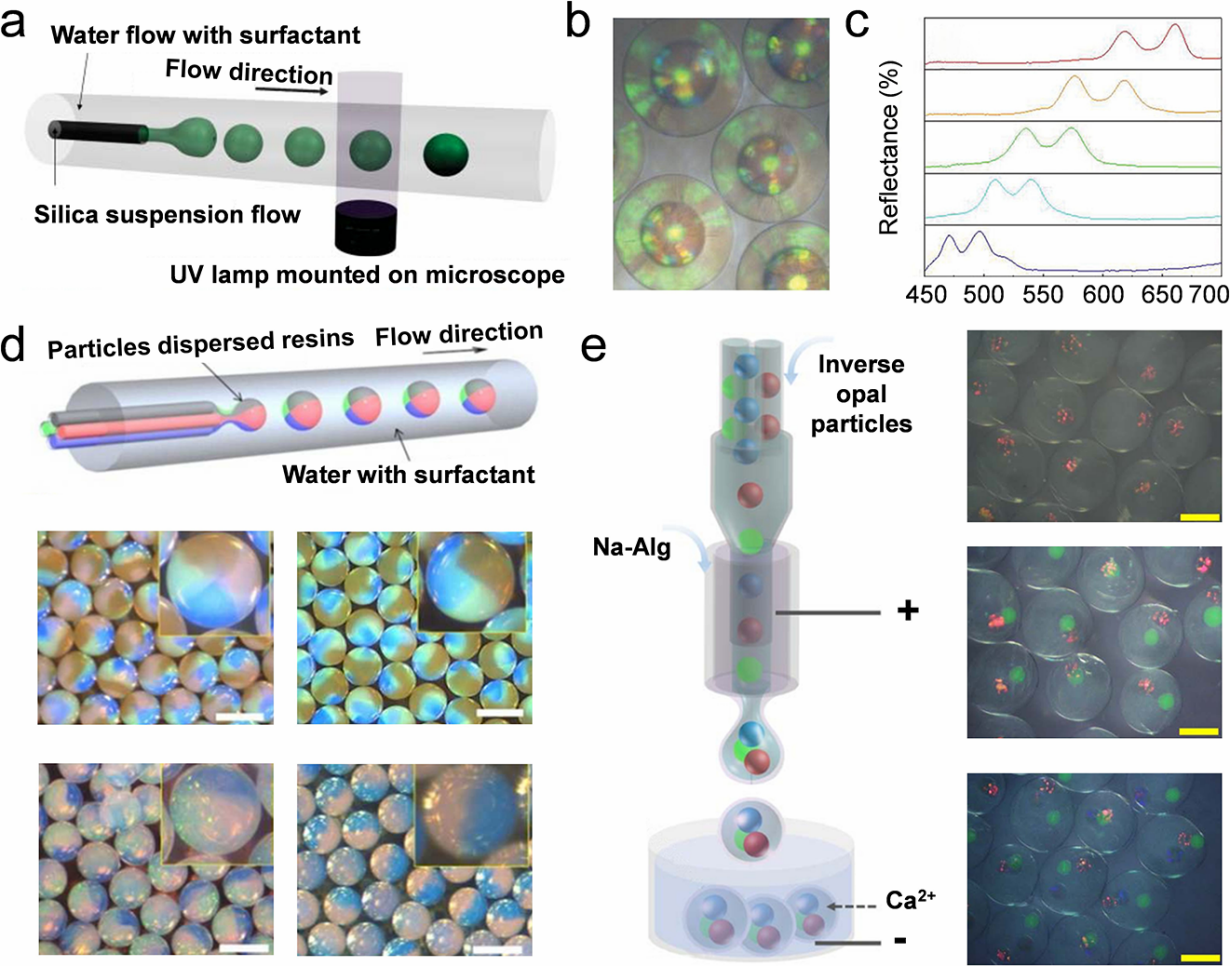
**Self-assembly of colloidal crystal microbeads by microfluidics.** a) Schematic illustration of the preparation of nonclosely packed colloidal crystal beads by microfluidics. The lattice constant depends on the ratio of the nanoparticles and UV-curable polymers. Reproduced with permission from ref 186. Copyright 2014 Wiley-VCH. b) Heterogeneous colloidal crystal beads with an inverse opal-shell and opal-core by partially etching nanoparticles from a colloidal crystal bead. Reproduced with permission from ref 186. Copyright 2014 Wiley-VCH. c) Reflectance spectra of the heterogeneous colloidal crystal beads made of different-sized nanoparticles. Reproduced with permission from ref 187. Copyright 2014 Wiley-VCH. d) Colloidal crystal beads with multiple components made by microfluidics with a multichannel. Reproduced with permission from ref 192. Copyright 2012 American Chemical Society. e) Microscope photographs of colloidal crystal bubbles with different cores made by electrospray microfluidics. Reproduced with permission from ref 193. Copyright 2018 The American Association for the Advancement of Science.

#### 4.1.5 Surface-Modified Substrate

Surface modification of substrates is an effective method for creating colloidal self-assembly dots and arrays, especially for metasurfaces composed of a 2D array of metamaterials.^195–197^ In general, the substrate employed in a colloidal self-assembly process features a uniform interface, thereby enabling homogeneous deposition of colloidal building blocks. A substrate with heterogeneous surface properties results in regioselective deposition of colloidal particles, allowing for control over the morphology of the resulting self-assembly.^198–200^ For example, a substrate with a patterned wettability enhances the substrate’s ability to harness colloidal building blocks, forming a colloidal crystal array.^138^ Besides the wettability, charge patterns on the substrate also facilitate regioselective colloidal assembly. For example, negatively charged Au nanoparticles can precisely anchor on a substrate where an array of positive charges is preconstructed. The Au nanoparticles work as the binding site for the anchoring of other nanoparticles (Figure 8a).^201^ However, such patterns do not provide selectivity regarding the assembled colloidal building blocks. DNA can resolve this issue by serving as anchors to accurately specify the location and type of colloidal building blocks that may be positioned (Figure 8b).^202^ The DNA can be connected to the hydrophilic zones pretreated with electron beam lithography (EBL). The Au nanoparticles are capped with DNA strands that are complementary to the sequences of the DNA extensions at the anchor points. Due to the selective bonding of DNA strands, Au nanoparticle monomers, dimers, and trimers can be precisely deposited on the anchors to form an array that functions as a metasurface. The metasurface can respond to polarized light and generate a plasmonic resonance effect.

**Figure 8:**
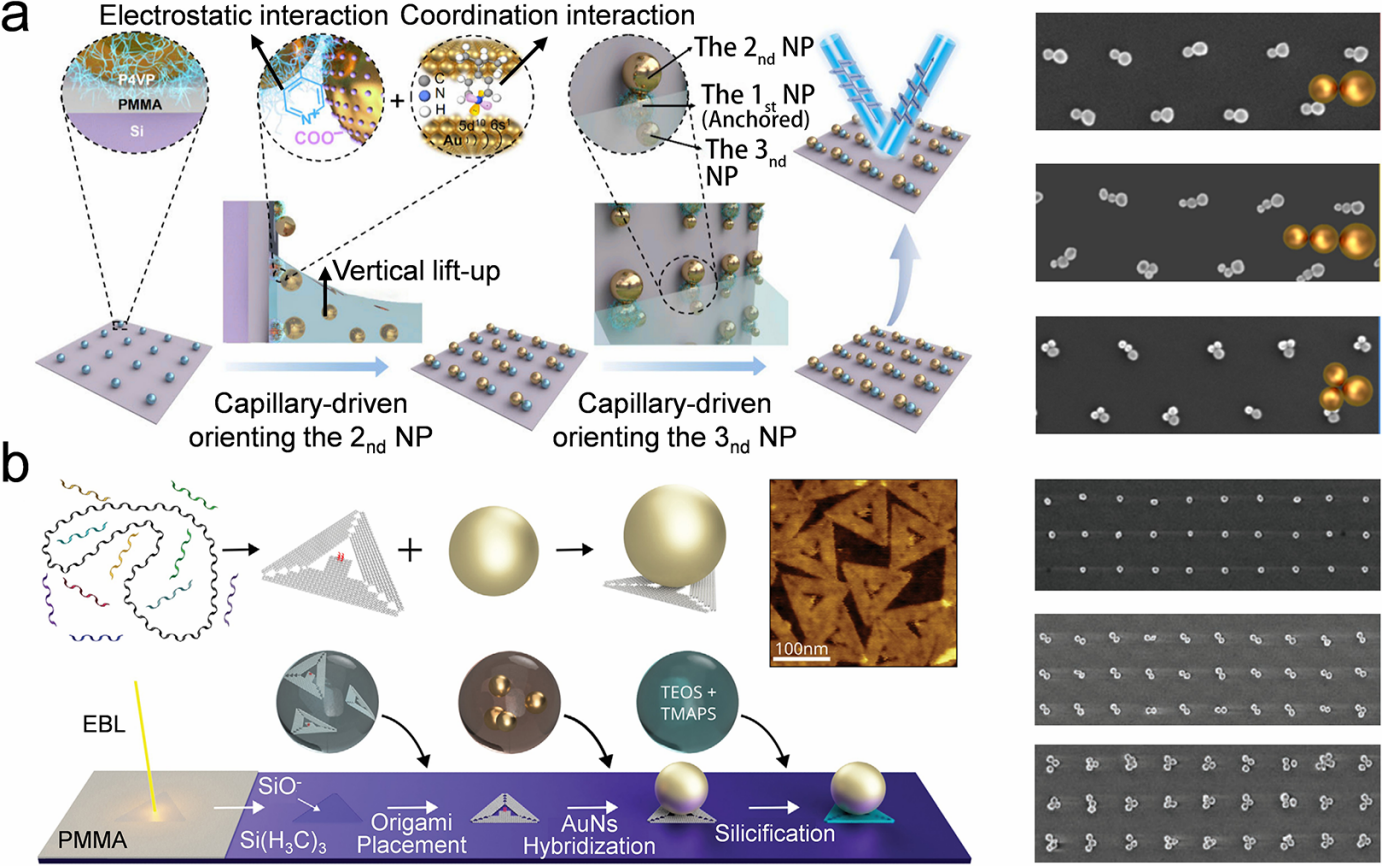
**Self-assembly of colloidal crystal dot array by surface-modified substrate.** a) Schematic of the trimer array fabrication process via site-specific sequential colloidal assembly. Reproduced with permission from ref 201. Copyright 2025 American Chemical Society. b) DNA origami-induced assembly of Au-based metasurface. DNA origami is connected to the hydrophilic zones that are pretreated by electron beam lithography (EBL). The Au nanoparticles are capped with DNA strands that are complementary to the sequence of the DNA extensions of the anchor point. Reproduced with permission from ref 202. Copyright 2024 Wiley-VCH.

#### 4.1.6 Summary

Dots of colloidal assemblies show high application potential because colloidal lines and patterns can be further developed using these dots.^140,141,203^ Colloidal self-assembly driven by droplet evaporation is a popular approach for fabricating colloidal dots. Numerous parameters within a drying droplet, including droplet internal flow, TCL, components, and drying conditions, can be systematically manipulated to tailor the resulting assemblies, thereby enhancing both their structural control and diversity. The patterned substrates present an effective method for fabricating individual colloidal dots or arrays. Table 4 provides an overview of colloidal self-assembly strategies to prepare colloidal crystal dots.

**Table 4:** Summary of Self-Assembly of Colloidal Crystal Dots

Self-assembly strategies	Key parameters	Outcomes	Remarks
Droplet inner flow	Substrate wettability, drying temperature, droplet compositions, and environmental humidity.	Coffee-ring stains, lines, plate-like dots, plain-like dots, and monolayer dots.	Several seconds to tens of minutes, outcome size from several μm^2^ to hundreds of μm^2^.
Droplet three-phase contact line	Substrate wettability, droplet composition, and substrate state.	Multiring depositions, colloidal crystal dots with programmable morphologies.	Several seconds to tens of minutes, outcome size from several μm^2^ to thousands of μm^2^.
Evaporation-triggered phase separation	Interaction between the nonvolatile components and colloidal building blocks, evaporation rate, and polymer concentration.	Self-assemblies with homogeneous and heterogeneous structures.	Several seconds to tens of minutes, outcome size from several μm^2^ to thousands of μm^2^.
Microfluidics	Colloidal concentration, suspension viscosity, substrate wettability, flow rate in channel, and temperature.	Colloidal microbeads with isotropic, anisotropic, and composite structures.	Several hours to days, outcome volume from several μm^3^ to thousands of μm^3^.
Surface-modified substrate	Wettability of the substrate and colloidal nanoparticles, colloidal concentration, and temperature.	Metasurface made of a 2D array of metamaterials, arrays of colloidal self-assemblies.	Tens of minutes to hours, outcome volume from several μm^3^ to thousands of μm^3^.

### 4.2 Self-Assembly of Colloidal Crystal Lines

In this review, colloidal crystal lines are elongated colloidal assemblies (e.g., fibers, linear arrays, chains) where one dimension significantly exceeds the other two. The primary significance of fabricating these lines lies in their role as programmable building blocks for designing complex patterns and 3D architectures. Various strategies have been developed for their construction, including droplet coalescence and direct-write printing. Additionally, assembly can be driven by using preconstructed structural templates on substrates. In this section, we will introduce strategies for assembling colloidal crystal lines.

#### 4.2.1 Colloidal Droplet Coalescence

As mentioned in the last section, colloidal crystal lines can be formed from dots of colloidal assemblies. Uniform droplets can be easily produced using an inkjet printer. However, it remains challenging to merge colloidal droplets into a continuous and smooth colloidal line because of the uncontrollable evolution of the footprints of the merging droplets. Many parameters influence the droplet coalescence, such as the droplet distance (Figure 9a).^140^ When printing colloidal droplets onto a viscoelastic PDMS substrate, the droplets may coalesce to form a well-organized line when the separation distance is approximately 20 μm. The separation distance depends on the droplet size and the rheological properties of the substrate. Additionally, parameters, including droplet space,^202,204^ drying temperature,^205,206^ and substrate wettability,^207^ also affect the coalescence of droplets. Furthermore, the intrinsic properties of the droplets, such as their viscosity and surface tension, also influence the coalescence of two droplets.^208,209^ The disparity in surface tension among the coalescent droplets is also of significant importance (Figure 9b).^141^ When the range of surface tension difference is between 0.77 and 1.50 mN/m, two droplets coalesce into a straight line. Otherwise, spherical caps and dumbbells are obtained. However, the resolution of the line via droplet coalescence is limited by the droplet size and the substrate wettability. To improve the resolution, the coffee-ring effect in a drying sessile droplet has been used since it can accumulate colloidal nanoparticles near the TCL (Figure 9c).^210^ By regulating the colloidal concentration and the wettability of the substrate, nanoparticles can effectively self-assemble into linear arrangements on a hydrophilic substrate with line widths of 5–10 μm, thereby enhancing the printing process resolution.

**Figure 9:**
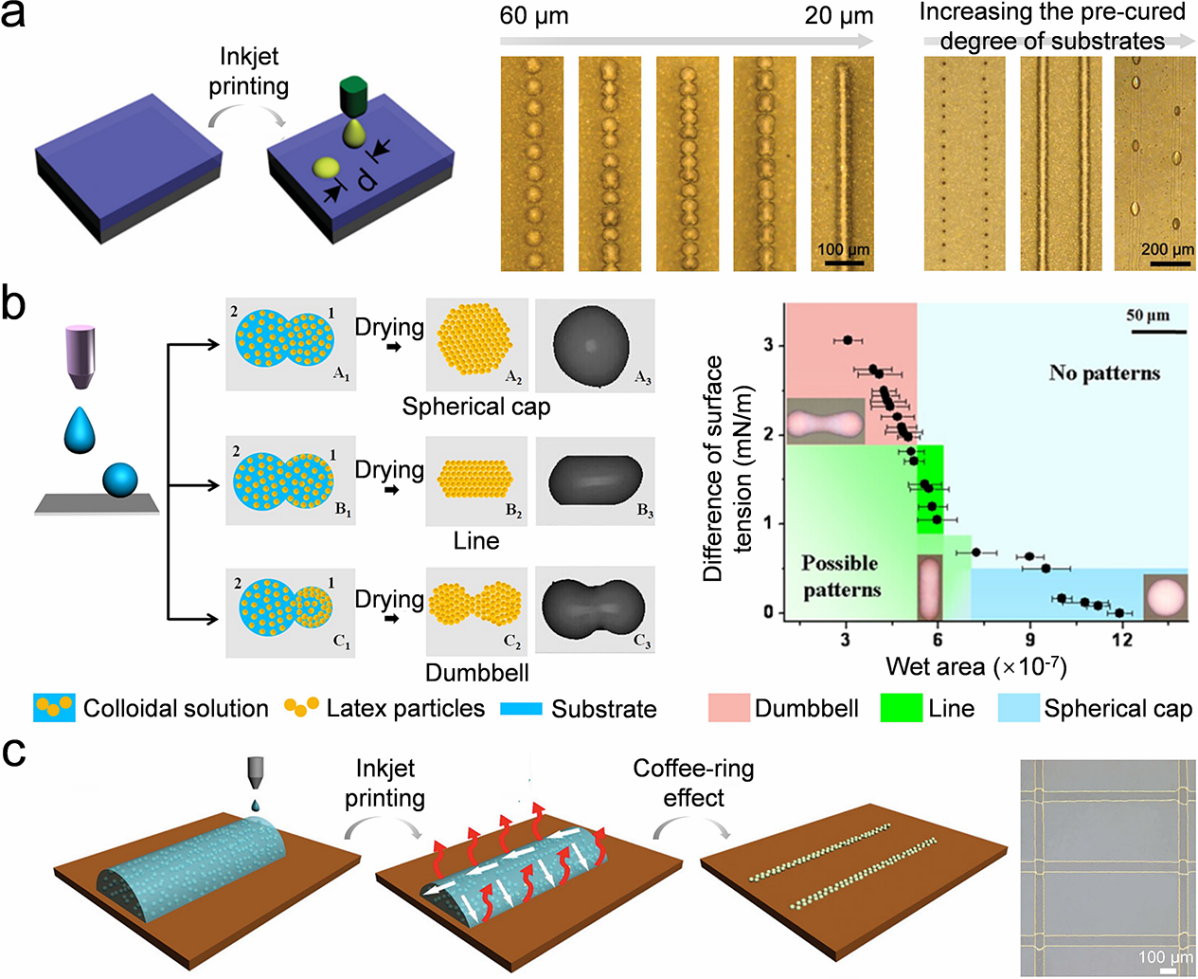
**Self-assembly of colloidal crystal lines by droplet coalescence.** a) Droplet coalescence behaviors on a viscoelastic surface. By decreasing droplet distance, droplets gradually coalesce into a straight line. The critical distance is related to the substrate rheology. Reproduced with permission from ref 140. Copyright 2015 Wiley-VCH. b) Coalescence behavior of droplets depending on the surface tension gap between two droplets. With different surface tension gaps, two droplets coalesce into spherical caps, straight lines, and dumbbells. Reproduced with permission from ref 141. Copyright 2014 American Chemical Society. c) Conductive lines assembled by silver nanoparticles using the coffee-ring effect. The outward capillary flow in the drying colloidal solution causes Ag nanoparticles to migrate to the edge of the liquid line. Reproduced with permission from ref 210. Copyright 2013 Wiley-VCH.

#### 4.2.2 Direct Write Printing

Colloidal crystal lines can also be fabricated through the direct extrusion of a colloidal suspension onto a substrate (Figure 10a).^211,212^ During extrusion, a nonuniform velocity profile forms within the extruded ink, leading to a unidirectional flow and shear force. This flow and shear force align nanoparticles into colloidal crystals. Viscosity of the colloidal ink is important, as low viscosity can cause pattern blurring and lower resolution, while high-viscosity inks may block the nozzle. Another challenge associated with direct writing printing is the Rayleigh–Taylor instability, which causes the continuous liquid lines to disintegrate into droplets. The instability originates from the interfacial tension of the liquid, which seeks to minimize the liquid volume to reduce its energy. In addition to increasing the viscosity of the ink, reducing the interfacial tension can also serve to mitigate the Rayleigh–Taylor instability. For instance, the air–liquid interface in standard printing scenarios may be substituted by an all-aqueous liquid–liquid interface to minimize interfacial tension. The reduced interfacial tension facilitates the formation of elongated liquid jets with customizable shapes. For example, a light-responsive reconfigurable colloidal assembly has been developed at the all-aqueous liquid–liquid interface. The capping ligands on the colloidal particles affect the light-responsive reconfiguration. Negatively charged silica nanoparticles and β-cyclodextrin-functionalized gold nanoparticles (βCD-AuNPs) can interact with positively charged azobenzene-based ionic liquids and assemble at the aqueous interface through both specific (photosensitive host–guest) and nonspecific (electrostatic) interactions. UV irradiation and acid treatment trigger the assembly and disassembly of the particles, controlling the interfacial permeability and tuning the structural integrity (Figure 10b).^213^ The low interfacial tension of liquid–liquid interfaces facilitates the reduction of Rayleigh–Taylor instability and further supports the design and fabrication of functional hierarchical structures.^214–218^

**Figure 10:**
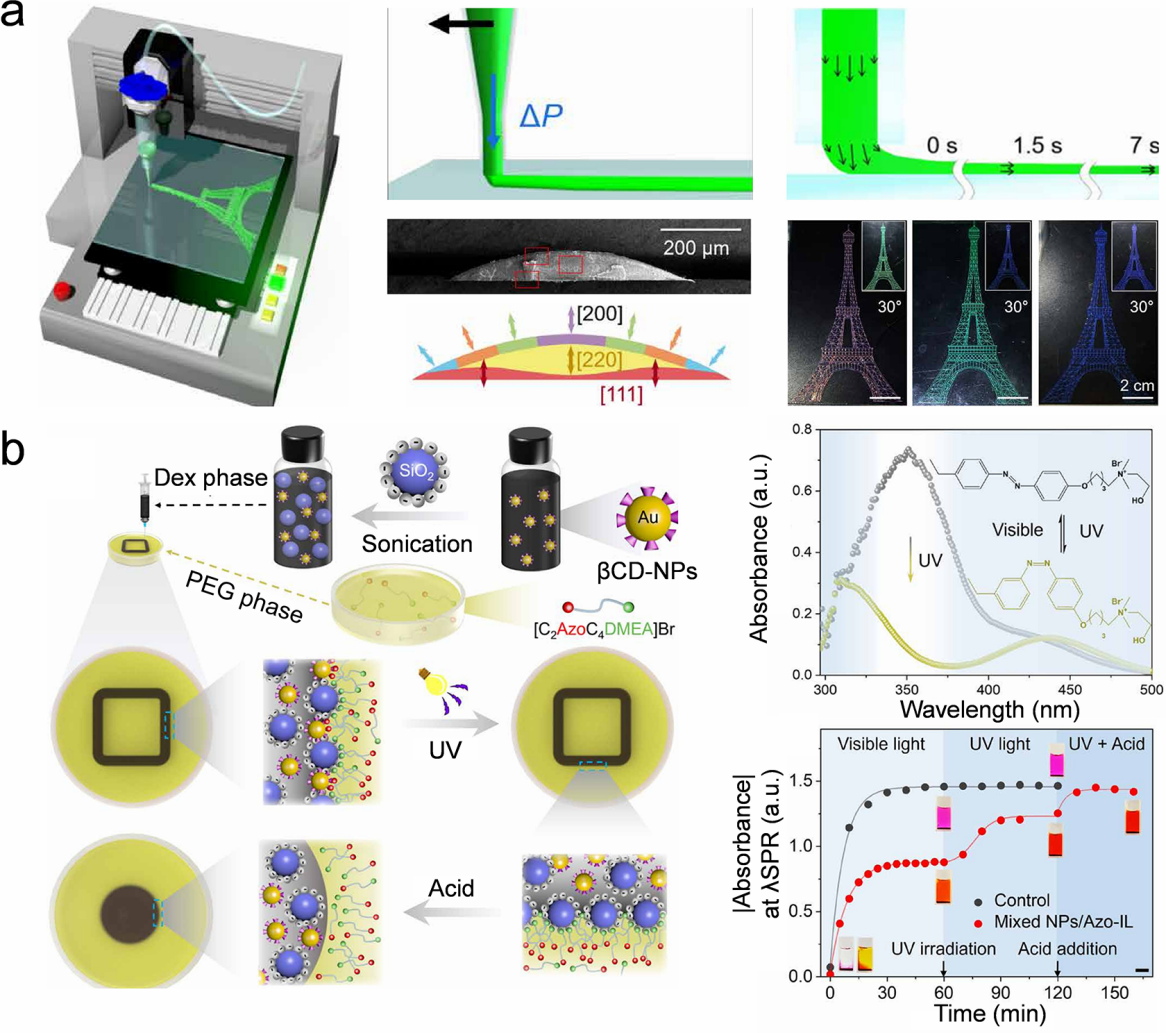
**Self-assembly of colloidal crystal lines by direct writing printing.** a) Direct writing of patternable colloidal assemblies with a commercial dispenser. The velocity profile results in a shear force and self-assembly of colloidal nanoparticles. Reproduced with permission from ref 212. Copyright 2021 The American Association for the Advancement of Science. b) Schematic representation of the aqueous printed structure with separate control over structural integrity and permeability, capitalizing on the different interactions between βCD-AuNPs/SiO_2_ NPs and azobenzene-based ionic liquids. Reproduced with permission from ref 213. Copyright 2024 The American Association for the Advancement of Science.

#### 4.2.3 Hard Templates

Another effective method for creating colloidal crystal lines involves using substrates with microstructures to guide the self-assembly process. These microstructures enable regioselective sedimentation of colloidal nanoparticles, directing both nucleation and the growth of the colloidal assembly. For instance, substrates featuring predesigned grooves can cause nanoparticles to organize into dimer chains, provided the groove size and periodicity align with the particle size (Figure 11a).^219^ The sedimentation process is affected by the template orientation, environmental temperature, and sedimentation rate.^220^ The acquired gold dimer chains are arranged into multilayers with varying tilting angles, imparting the structure with a circular dichroism of up to 11°. Owing to the elastic template, the chiral meta-surfaces are mechanically tunable, thereby enhancing the capabilities of colloidal self-assembly. The regioselective sedimentation of colloidal nanoparticles generates patterned self-assemblies. For instance, nanoparticles can hardly assemble on nanoneedle arrays owing to minimal particle-to-needle overlap of excluded volumes and weak adhesive forces, thereby inhibiting the nucleation of colloidal assemblies in such regions. Conversely, planar surfaces facilitate the nucleation and growth of colloidal crystals due to significant particle-to-plane volume overlap and strong adhesive forces. As a result, photonic micropatterns can be readily fabricated on substrates featuring micropatterns with planar surfaces and nanoneedle arrays (Figure 11b).^221^ In addition to the crystal lines and patterns, microstructural substrates can influence the arrangement of the colloidal crystal lattice. During the process of self-assembly, nanoparticles typically organize into a FCC lattice with their (111) planes aligned parallel to the substrate.^222,223^ Consequently, the majority of optical measurements on colloidal assembly have been confined to the (111) orientation of the FCC lattice or the L-point of their reciprocal lattice. Substrates featuring preconstructed microstructures have been employed to guide the colloidal self-assembly, thereby expanding the diversity of self-assembled plane orientations.^224^ For example, utilizing substrates equipped with predesigned hole arrays facilitates the formation of colloidal assemblies with a (100) plane orientation or a heterogeneous packing structure because of colloidal processes epitaxy.^225–227^

**Figure 11:**
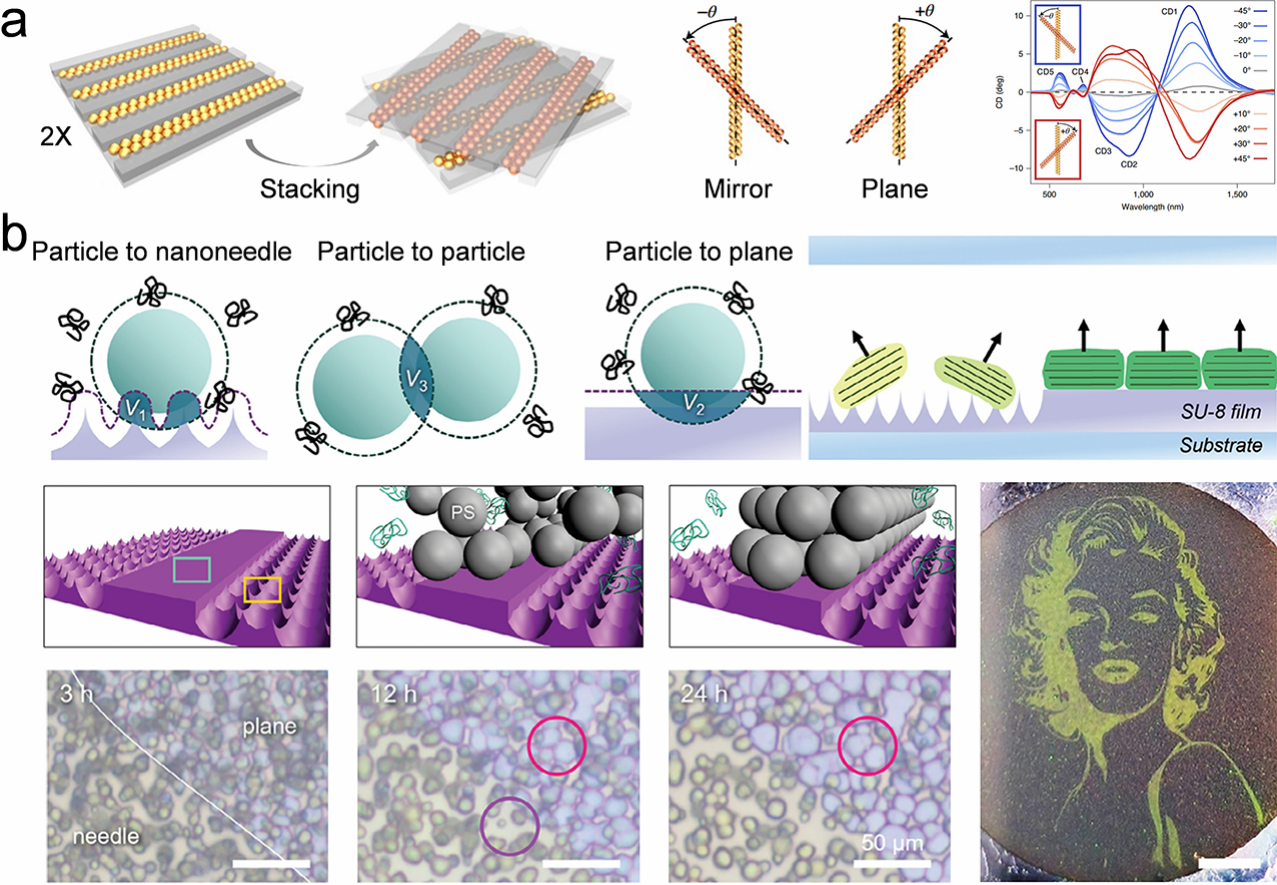
**Self-assembly of colloidal crystal lines by hard templates.** a) Fabrication of chiral meta-surfaces with the assistance of elastic nanochannels made by e-beam free nanofabrication scheme. The Au dimer chains are stacked into multilayers with different tilting angles, endowing the structure with a circular dichroism of up to 11°. Reproduced with permission from ref 219. Copyright 2022 Springer Nature. b) Photonic micropatterns induced by substrates with patterned microstructures. Particles can hardly assemble on nanoneedle arrays because of the minimal particle-to-needle overlap of excluded volumes and weak adhesion force. Reproduced with permission from ref 221. Copyright 2023 Wiley-VCH.

#### 4.2.4 Soft Templates

In addition to solid templates, fluid templates are a practical alternative for guiding colloidal self-assembly. A common example is bubbles, which have been extensively used to directly assemble molecules, hollow spheres, chiral structures, tubular forms, and mesh structures.^228–233^ One of the challenges associated with utilizing bubbles as templates is the regulation of their shape and morphology, given that the spontaneous evolution of 2D foams adheres to the Oswald ripening process (where larger bubbles consistently engulf smaller ones). The phenomenon of Oswald ripening can be managed through a microstructural template.^234^ By altering the configuration of pillars on the substrate, 2D foams with customizable sizes, shapes, patterns, and arrangements (Figure 12a). These patternable foams serve as templates that facilitate the assembly of nanoparticles into specifically desired two-dimensional networks. The bubble film progressively narrows due to evaporation, thereby providing a gradually confined space for the nanoparticles. Subsequently, the nanoparticles adhered to the film may be organized periodically in conjunction with the patterned bubbles. Additionally, bubble templates have been employed to assemble Janus particles with modified wettability, facilitating the arrangement of Janus particles into heteraxial or coaxial nanowires with the assistance of the bubbles.^235^

**Figure 12:**
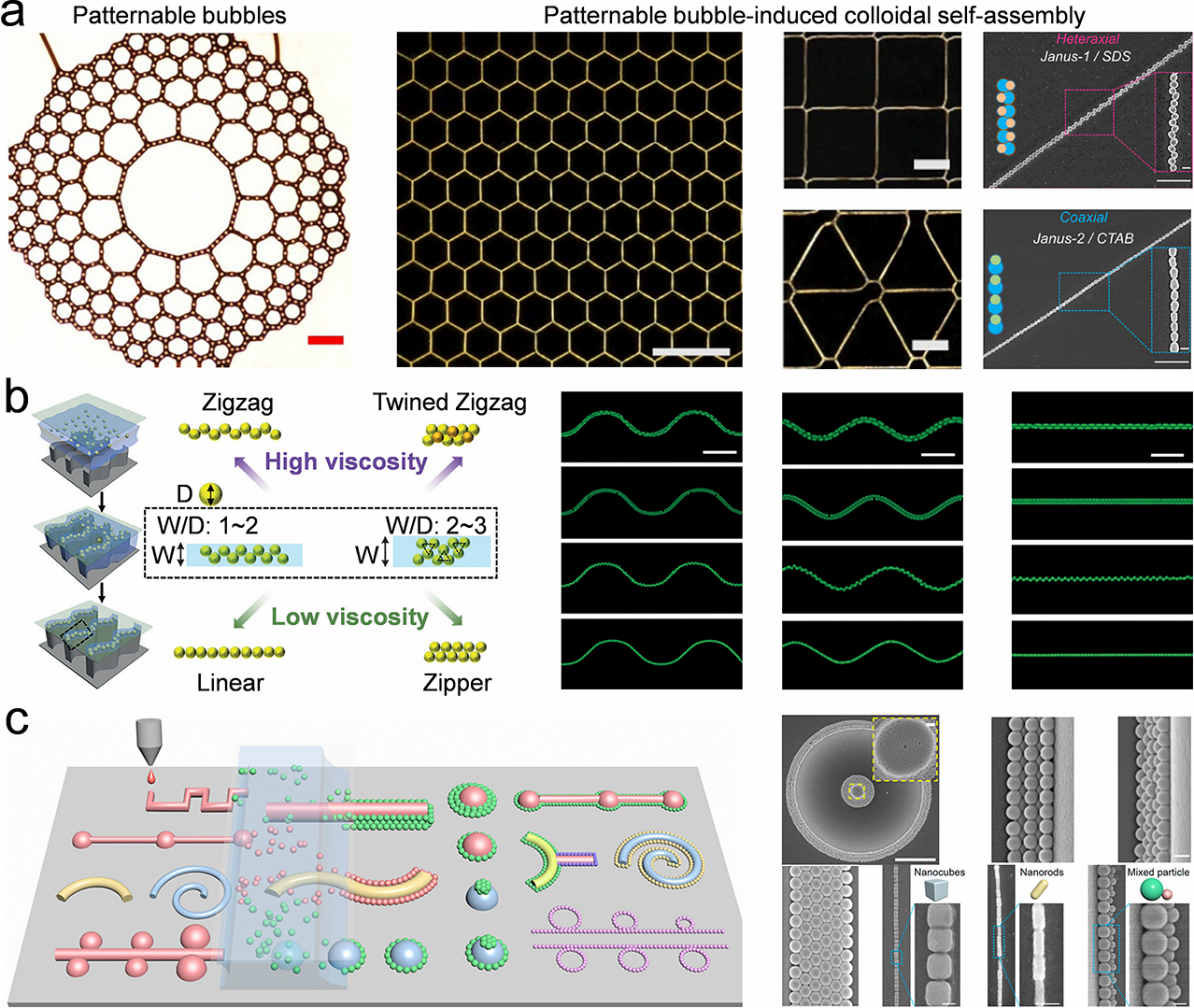
**Self-assembly of colloidal crystal lines by soft templates.** a) Patternable foam template and resultant self-assemblies of various particles. The microstructures on the substrate overcome the Oswald ripening, guiding the formation of 2D foams with programmed sizes, shapes, patterns, and positions. Reproduced with permission from refs 234, 235 . Copyright 2017 Springer Nature under CC BY 4.0 (http://creativecommons.org/licenses/by/4.0/). Copyright 2023 American Chemical Society. b) Control of zigzag or linear assembly from isotropic particles induced by micropillars on silicon substrates and fluorescent images of the resultant structures. Reproduced with permission from ref 236. Copyright 2017 Wiley-VCH. c) Schematic illustration of the optical heterostructure patterning by spatially allocating nanoparticles on a printed template and SEM images of the resultant structures. Reproduced with permission from ref 238. Copyright 2022 American Chemical Society.

Soft templates also involve colloidal suspensions sandwiched between a superhydrophobic substrate and a superhydrophilic glass slide (Figure 12b).^236^ Micropillars or microwalls are constructed on the substrate. During the drying process of the colloidal suspension, a liquid-confined system is established between the apex of the micropillar and the cover slide. Facilitated by evaporation-induced capillary flow, particles assemble within the confined liquid space concurrently with the solvent’s evaporation. The resulting assemblies are highly dependent on the liquid’s viscosity, leading to the formation of zigzag, twined zigzag, and linear or zipper-like chains. Based on this liquid-confined assembly process, the programmed coassembly of 1D binary hierarchical structures and multicomponent assembly patterns can be realized with the aid of various substrate templates and building blocks.^78,237^

Microstructural templates are essential for programming bubble films or systems confined by liquids. Creating these templates involves complex micro- or nanofabrication techniques. Inkjet printing has been used to produce polymer templates for the simple fabrication of the template (Figure 12c).^238^ Using an inkjetting polymer template, diverse building blocks such as nanoparticles, nanocubes, nanorods, and mixed particles can be precisely positioned on the printed matrix through liquid-based confined assembly. This process enables the creation of 1D, 2D, or 3D heterogeneous photonic structures. This templating method expands fabrication options beyond traditional multistep nanofabrication. Unlike conventional templates, the printed template can gain advanced functionalities, such as photoelectric properties and responsiveness, by replacing polymer ink with functional materials. This allows the integrated development of multiple functions within the resulting nanostructures.

#### 4.2.5 Summary

Droplet coalescence is a widely used approach for creating colloidal crystal lines; however, it requires precise control over droplet size, droplet spacing, substrate properties, and surface tension of the droplets. Additionally, direct writing of colloidal suspensions onto substrates presents a promising strategy, although it is essential to mitigate Rayleigh instability. Templates provide an effective approach for fabricating colloidal assemblies, including dots, arrays, clusters, lines, and patterns. It also holds promise for constructing distinctive superlattices with specific plane orientations or hierarchical arrangements, thereby expanding the variety of self-assembled structures. Nevertheless, the production of the necessary templates entails an expensive and complex top-down fabrication process, which restricts their broad applicability. Consequently, some other templates, like a substrate with patterned modification of chemicals,^239,240^ ice crystals,^241^ inverse opals,^242^ capillary tubes,^243,244^ self-assembled block copolymers,^245^ and Emil Fisher’s lock-and-key binding,^246–250^ have been applied to replace the traditional lithographical templates. The functionalization of the template is an exciting area of research because it can improve the functionalities of the resulting structures. Notably, several additional strategies, including field-induced colloidal self-assembly,^251^ anisotropic colloidal self-assembly,^252^ and 3D printing,^128^ can also be employed to fabricate colloidal crystal lines. These approaches are detailed in Section 5. A detailed overview of template-confined colloidal self-assembly strategies is provided in Table 5.

**Table 5:** Summary of Self-Assembly of Colloidal Crystal Lines

Self-assembly strategies	Key parameters	Outcomes	Remarks
Droplet coalescence	Droplet size, droplet distance, droplet space, droplet viscosity, droplet surface tension, and surface tension difference between the coalescent droplets.	Colloidal crystal lines, spherical cap of colloidal crystals, dumbbell-shaped colloidal crystals.	Several minutes to hours, outcome length from tens μm to tens of cm.
Direct write printing	Viscosity of the colloidal suspension, interface tension of the extruding colloidal suspensions.	Colloidal crystal lines and fibers.	Several minutes to hours, outcome length from tens μm to hundreds of cm.
Hard templates	Template orientation, environmental temperature, sedimentation rate, and how to match the colloidal size and template size.	Films and patterns of colloidal assemblies with special packing lattices.	Several minutes to hours, outcome length from tens μm to several cm.
Bubbles	Colloidal concentration, colloidal wettability, a substrate with preconstructed microstructures, the viscosity of the colloidal suspension, and bubbles.	Patterns of colloidal self-assemblies, colloidal chains.	Several minutes to hours, outcome length from tens μm to several cm.
Liquid bridge	Wettability of the substrate and cover glass, colloidal concentration, and viscosity of the colloidal suspension.	Colloidal chains, dots.	Several minutes to hours, outcome length from tens μm to several cm.
Substrate with printed structures	Wettability of the substrate and cover glass, colloidal concentration, and viscosity of the colloidal suspension.	Colloidal chains, dots.	Several minutes to hours, outcome length from tens μm to several cm.

### 4.3 Self-Assembly of Colloidal Crystal Films

Colloidal crystal films show great potential applications in packaging, decoration, and anticounterfeiting. Particularly, colloidal crystal films with high quality are of significance in laboratory research. Diverse strategies are developed to fabricate colloidal crystal films, including vertical deposition, bar coating, and spin coating. In this section, we will introduce these strategies according to their driving forces.

#### 4.3.1 Meniscus Capillary Force

A meniscus forms near the droplet TCL when the substrate is highly wettable by the liquid. For example, in vertical deposition self-assembly, a superhydrophilic substrate is submerged vertically in a hydrophilic colloidal suspension within a controlled environment of constant temperature and humidity (Figure 13a). The hydrophilic nature of the substrate leads to the formation of a liquid meniscus. As the solvent evaporates, hydrophilic nanoparticles migrate toward the meniscus due to convective flow caused by capillary forces. Near the meniscus, colloidal nanoparticles assemble into tightly packed structures, producing vivid structural colors because of their periodic arrangement (Figure 13b). However, cracks develop in the colloidal film, caused by the sudden release of in-plane tensile stress when the solvent evaporates.^253^ Cracks develop along a specific plane when the in-plane tensile stress exceeds the fracture resistance in that plane.^254^ Defects in colloidal crystals facilitate the generation of cracks. Defects could arise due to colloidal polydispersity, particle shape variability, and impurities,^255^ as well as the disturbances from surrounding environments (vibrations, airflow, and temperature fluctuation) during the self-assembly process. The defects serve as tensile relief points for crack formation and can be artificially introduced into colloidal self-assemblies to control crack development (Figure 13c).^254^ For instance, micronotches of various configurations have been employed to facilitate crack formation in a colloidal film: Diamond-shaped micronotches are used to induce highly uniform cracks along the (110) plane; handle-like micronotches on the substrate generate cracks in multiple directions. Consequently, colloidal crystal blocks of predetermined sizes and dimensions are produced.^254^ Nevertheless, the presence of cracks fails the resulting materials or devices. Numerous efforts have been undertaken to prevent crack formation in colloidal self-assemblies.^51^ A strategy is to use “glue” (pregel) to connect the assembling nanoparticles to improve the fracture resistance along the crystal plane that inhibits the formation of cracks.^256^ Utilizing the interconnected gel precursor, cracks can be effectively prevented in a film comprising up to 18–20 particle layers. Consequently, crack-free inverse opal films constructed from silica, titania, alumina, and zirconia, with dimensions reaching up to centimeters, can be prepared.^257,258^ Using the vertical deposition technique, homogeneous and heterogeneous colloidal films can be fabricated on a patterned substrate^259^ or by multistep deposition.^260^ Nanoparticles can also aggregate into ordered films by dip-coating, in which a superhydrophilic substrate is gradually pulled out vertically from a colloidal suspension (Figure 13d).^79^ In the pulling process, a liquid meniscus forms near the substrate, where hydrophilic nanoparticles aggregate closely due to the capillary force induced by the evaporating meniscus. The dip-coating strategy is widely used in fabricating 2D or 3D colloidal crystals, but the slow withdrawal rate limits its throughput.^261^ A similar approach is to assemble colloids into films by extracting the colloidal suspension instead of pulling out the substrate (Figure 13e).^262^ In the demonstration, two colloidal dispersions are simultaneously infused and extracted at different rates, leading to a slowly falling interface and a thin film with gradually changing composition. This strategy can construct heterogeneous structures assembled by various colloidal nanoparticles.

**Figure 13:**
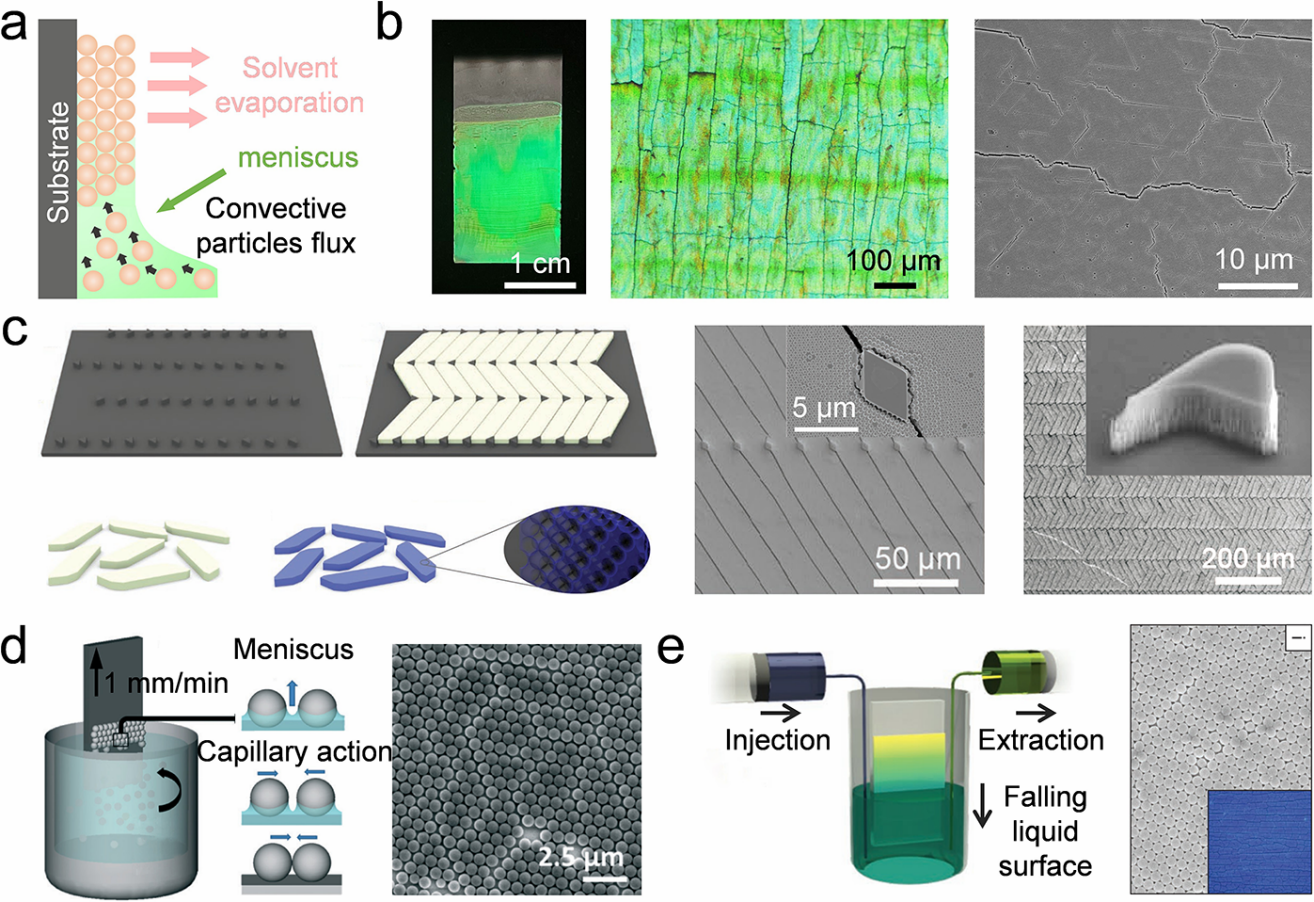
**Meniscus capillary force-induced self-assembly of colloidal crystal films.** a) Schematic diagram of the vertical deposition process. Nanoparticles assemble at the meniscus of the air–liquid-substrate contact line due to the capillary force-induced convective flux. b) Optical and SEM images of a colloidal crystal film made by the vertical deposition process. Cracks occur in the well-assembled entity. Reproduced with permission from authors. Copyright authors. c) A schematic diagram shows the process of programming the formation of cracks. Micronotches with different shapes are constructed on a substrate as stress relief points to engineer the cracks and fabricate photonic bricks with designed sizes and geometries. Reproduced with permission from ref 254. Copyright 2019 Wiley-VCH. d) Dip-coating method to assemble colloidal nanoparticles and the SEM image of the resultant well-organized crystal. Reproduced with permission from ref 79. Copyright 2014 Royal Society of Chemistry. e) Infusion withdrawal coating technique to fabricate a gradient colloidal assembly. By infusing and extracting two colloidal solutions simultaneously at different rates, orderly packed nanoparticles could be obtained due to the gradually decreasing liquid surface. Reproduced with permission from ref 262. Copyright 2021 Wiley-VCH under CC BY 4.0 (https://creativecommons.org/licenses/by/4.0/).

#### 4.3.2 Shear Force

The vertical deposition process for colloidal assembly is easy to perform but time-consuming, making it difficult to produce large-scale films spanning tens of centimeters or meters. As an alternative, the bar coating technique has been developed, which involves spreading colloidal suspension on a preheated substrate using a moving razor blade to assemble colloids into crystal films (Figure 14a).^83^ The motion of the blade directionally aligns nanoparticles along with the induced transverse shear force, thereby facilitating the formation of a self-assembled colloidal film with a precisely controlled thickness determined by the distance between the blade and the substrate. A roll-to-roll technique has been developed to enhance the scalability of these self-assembled colloidal films, enabling the formation of flexible films through ordered aggregation (Figure 14b).^263^ The flexibility arises from the sandwich configuration, wherein two polymer sheets enclose the colloidal film. Bending the laminate around cylinders induces a shear force parallel to the polymer surface within the colloidal layer, thereby compelling nanoparticles to assemble into an ordered colloidal structure. Due to the protective encapsulation by the polymer sheets, the resulting colloidal films exhibit high resistance to environmental variations, including mechanical alterations, pH fluctuations, and humidity changes. Nonetheless, the bar coating or roll-to-roll strategies are limited to assembling colloids into films owing to the geometric constraints of blades and rolls.

**Figure 14:**
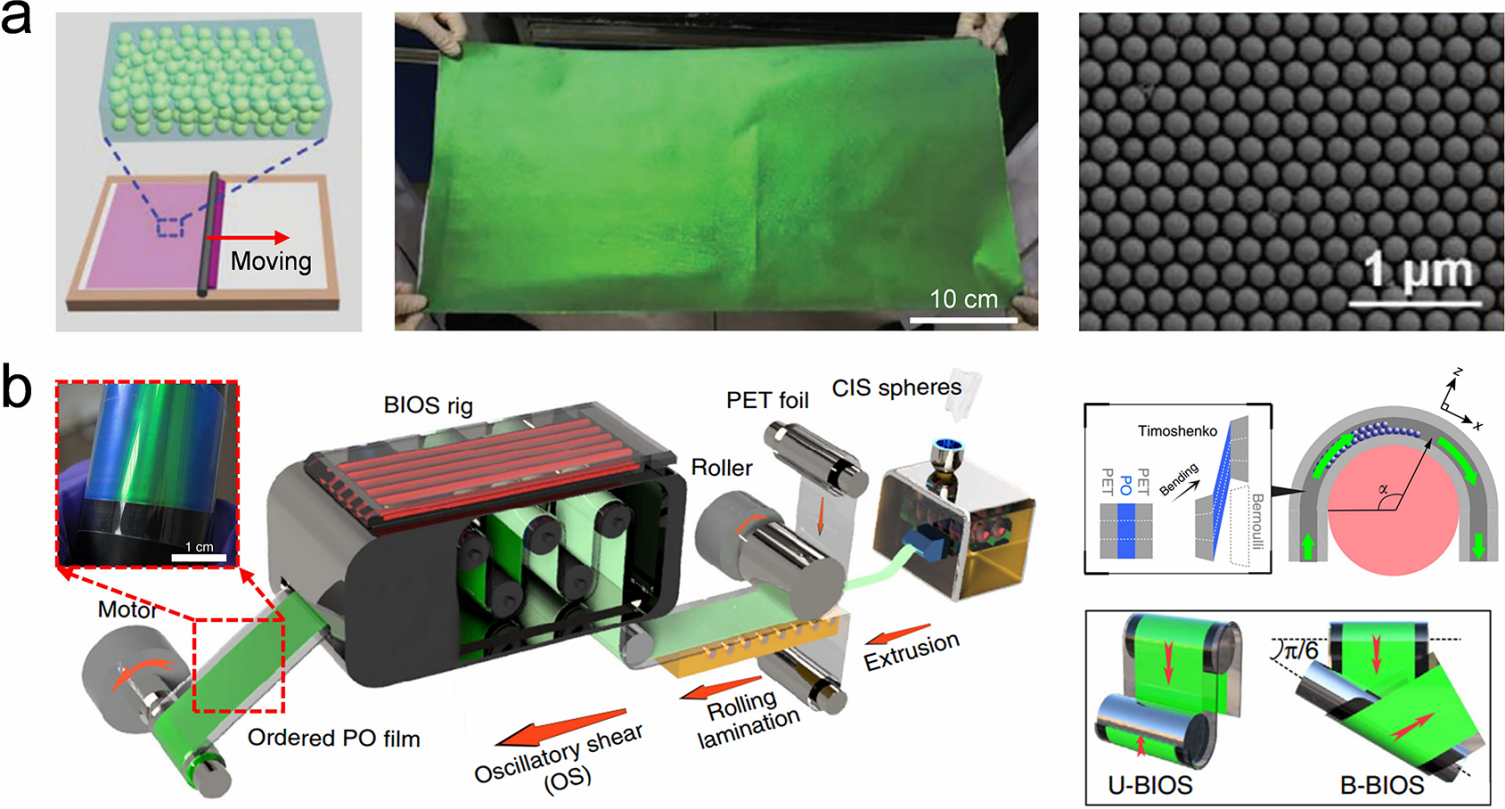
**Shear force-induced self-assembly of colloidal crystal films.** a) Schematic illustration of the bar coating technique to assemble colloidal nanoparticles and the optical and SEM images of the resultant colloidal self-assembly. The motion of the bar generates a shear force that aligns nanoparticles periodically. Reproduced with permission from ref 83. Copyright 2021 Wiley-VCH. b) Production line of a roll-to-roll strategy to scale up the production of colloidal films. The bending of a laminate colloidal suspension creates a shear force. Inset is the bent colloidal crystal between PET foils made by the roll-to-roll method. Reproduced with permission from ref 263. Copyright 2016 Springer Nature under CC BY 4.0 (https://creativecommons.org/licenses/by/4.0/).

#### 4.3.3 Other Strategies

The spin coating technique has been employed to produce films of two-dimensional or three-dimensional colloidal crystals, given its scalability, simplicity, and efficiency.^264–266^ In a demonstration, a colloidal suspension with appropriate concentration is dispensed onto a hydrophilic substrate, which is placed on a spin coater (Figure 15a).^267^ Using the spinning substrate, colloidal nanoparticles assemble into an ordered structure, driven by centrifugal force. The thickness of the structure is dependent on the spinning speed and colloidal concentration.^264^

**Figure 15:**
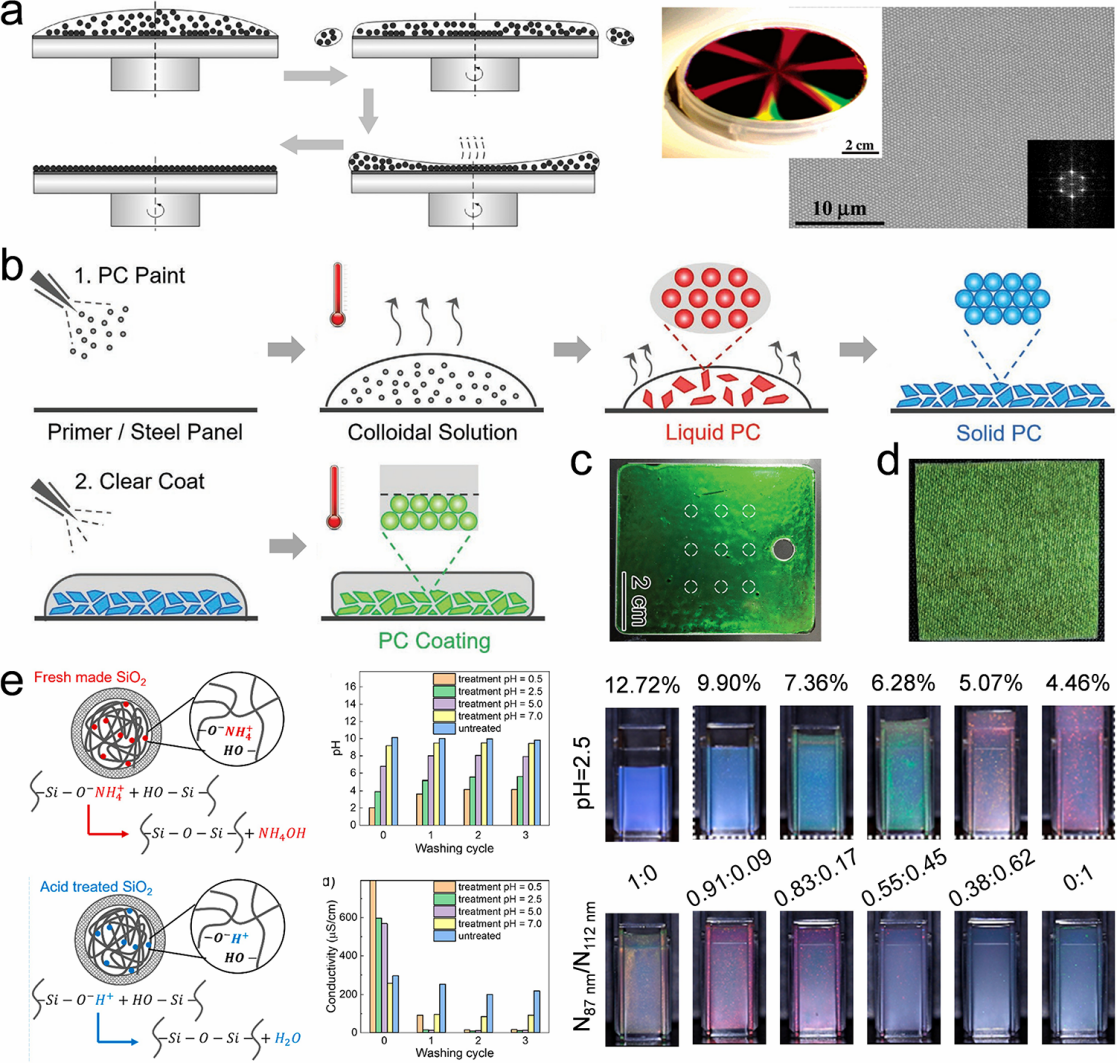
**Other strategies for self-assembly of colloidal crystal films.** a) Schematic illustration of the spin-coating technique to prepare colloidal crystals. With the spinning substrate, colloidal nanoparticles assemble into an ordered structure driven by the centrifugal force. Reproduced with permission from refs 264, 267 . Copyright 2013 Published by Elsevier. Copyright 2004 American Chemical Society. b–d) Procedures for self-assembly of colloidal crystals by the two-step spray coating method. During the drying process, capillary force drives nanoparticles to assemble periodically. Reproduced with permission from refs 80, 274 . Copyright 2020 Wiley-VCH. Copyright 2022 Wiley-VCH. e) The NH_4_^+^ ions trapped in the silica nanoparticles are removed by acid treatment, preventing their release to the solution while maintaining a highly charged surface. The resultant silica nanoparticles achieve self-assembly in a large concentration range and of a colloidal system with two distinct sizes. Reproduced with permission from ref 276. Copyright 2023. American Chemical Society.

Spray coating can assemble colloidal building blocks in a patternable manner on various substrates.^268^ In a conventional spray coating procedure, colloidal building blocks dispersed in a volatile solvent are applied to a preheated substrate, where they subsequently aggregate following the evaporation of the solvent.^269,270^ Despite the universality of this strategy, the resultant objects are amorphous colloidal glasses.^271–273^ To enhance the crystallization of sprayed colloidal self-assemblies, a two-step spraying methodology has been developed. This procedure involves initially spraying a colloidal ink composed of silica nanoparticles, propylene carbonate, and ethanol onto the substrate. (Figure 15b).^80^ Following the spraying process, the ink viscosity elevates due to the rapid evaporation of ethanol, facilitating the formation of liquid photonic crystals. Subsequently, the substrate is placed in an oven to induce nanoparticle precipitation, resulting in the formation of liquid colloidal crystals. Through the gradual evaporation of propylene carbonate, the nanoparticles are provided with sufficient time to periodically arrange themselves on various substrates, thereby creating a highly ordered structure and producing a vibrant structural color. (Figures 15c and 15d).^274,275^

The strategies previously mentioned generally necessitate the use of highly monodisperse and concentrated colloidal building blocks, specifically exceeding 30 vol %, particularly within silica colloidal systems. During synthesis, NH_4_^+^ ions become entrapped within the silica core, and they can later be released, resulting in an increased ionic strength of the solution. This elevated ionic strength causes a compression of the double electric layer surrounding the silica nanoparticles. Such compression disrupts long-range interparticle interactions and augments the sensitivity of colloidal self-assembly to the degree of colloidal monodispersity. The NH_4_^+^ can be replaced by H^+^ by an acid treatment, thus preventing it from releasing into the solution. The acid treatment diminishes the ionic strength of the solution while preserving a consistent surface charge on the silica nanoparticles (Figure 15e). Consequently, silica nanoparticles can self-assemble at relatively low concentrations and show pH-responsive self-assembly behavior within a specific concentration range. Furthermore, the reduced ionic strength lessens the constraints on the uniformity of the assembly process, thereby enabling silica nanoparticles to be assembled even within a mixture containing two distinct types of silica nanoparticle sizes.^276^

#### 4.3.4 Summary

Besides the strategies mentioned above, other strategies, such as centrifugation,^81,82^ can also achieve the self-assembly of colloidal nanoparticles to prepare colloidal crystal films. Various colloidal self-assemblies can be obtained through different strategies. Specifically, the vertical deposition process is optimal for producing high-quality crystal films driven by meniscus capillary forces; however, it is time-consuming and possesses limited scalability. The bar-coating and roll-to-roll techniques are well-suited for effectively scaling up the self-assembly of colloids driven by shear forces, although they are unable to produce patterned structures. The choice should be carefully considered according to different application scenarios (Table 6).

**Table 6:** Summary of Self-Assembly of Colloidal Crystal Films

Self-assembly strategies	Key parameters	Outcomes	Remarks
Vertical deposition	Superhydrophilic substrate, constant temperature and humidity (80 °C-80% or 60 °C-60%), colloidal concentration, size, density, and monodispersity of nanoparticles.	Colloidal films.	Several days, film size around several cm^2^, and meniscus pinning.
Dip coating	Pulling speed, superhydrophilic substrate, colloidal concentration, size, monodispersity, and evaporation rate of the solvent.	Colloidal films.	Several hours, film size from tens to hundreds of cm^2^, suitable for unflattened substrates.
Bar coating	Temperature, superhydrophilic substrate wettability, colloidal concentration, size, and monodispersity, moving speed of the bar, and evaporation rate of the solvent.	Colloidal films.	Tens of minutes to hours, film size from tens to hundreds of cm^2^.
Roll-to-roll	Colloidal concentration, pulling speed, superhydrophilic substrate, and evaporation of the solvent.	Colloidal films.	Tens of minutes to hours, film size from tens of dm^2^ to hundreds of m^2^.
Spin coating	Spin speed and time, colloidal concentration, size, and monodispersity, superhydrophilic substrate, suspension viscosity, and evaporation rate of the solvent.	Colloidal films.	Tens of minutes to hours, film size from tens to hundreds of cm^2^.
Spray coating	Colloidal concentration, superhydrophilic substrate, and evaporation rate of solvent.	Colloidal patterns and films.	Tens of minutes, waste of colloidal solution, film size from tens to hundreds of cm^2^.
Centrifugation	Centrifugal speed, centrifugal force, centrifugal time, colloidal concentration, suspension viscosity, and colloidal surface properties.	Bulk colloidal crystals.	Tens of minutes to hours, film size from tens to hundreds of cm^2^.

## 5 Advanced Strategies for Colloidal Self-Assembly

The colloidal self-assembly strategies discussed above predominantly rely on the evaporation of colloidal suspensions. The resulting self-assemblies are programmable through the intrinsic properties of the colloidal system, such as the evaporation rate, colloidal concentration, surface tension, viscosity of the suspension, and substrate properties. There is an increasing demand for colloidal self-assemblies with intricate structures, including three-dimensional architectural configurations, chiral structures, among others. This demand drives the development of more sophisticated colloidal self-assembly strategies. In this section, several advanced strategies capable of self-assembling colloidal nanoparticles into specialized structures are reviewed (Table 7). These strategies are classified as advanced due to their enhanced degree of programmability in the resulting structures. Their operations, recent advancements, application scenarios, advantages, and limitations of each strategy are also discussed.

**Table 7:** Advanced Colloidal Self-Assembly Strategies

	Design Principles	Dominated driving forces	Obtained structures	Advantages	Disadvantages	References
3D printing-assisted colloidal self-assembly	Direct ink writing	Shear force	Fibers, 3D architecture objects	Programmable macro architecture	Low degree of order, step-effect	^280^,^282^,^289–291^
	Digital light processing	Solvation force and particle interaction	3D architecture objects	Programmable macro architecture	Low degree of order	^292–295^
Interfacial colloidal assembly	Air–liquid interface	Interfacial forces	Colloidal films or pixels	Hierarchical multilayer films or monolayer structures	Defects	^297–314^
	Liquid–liquid interface		Colloidal films	High regulatability	Difficulty in precisely controlling the structure	^216^,^315–317^
			Colloidosome	Facile hierarchical colloidal structure		^318–320^
External field-manipulated colloidal assembly	Electrical field	Electric force	Colloidal crystal dots, lines, and films	Customizable patterns, heterogeneous structures	Requirements of charged colloids, a slow process, and an electrolytic reaction	^84^,^95^,^334–336^
	Magnetic field	Magnetic force	Colloidal crystal dots, lines, films	Customizable patterns, heterogeneous structures, and integration of different properties	Requirements of magnetic material in the building blocks	^323^,^340–344^
	Light field	Light potential, optoelectrical effect, optothermal effect	Colloidal crystal dots, lines, patterns	Regulable in many pathways	Low degree of order	^345–361^
	Acoustic field	Mechanical flow	Arrays, stripes, patterns	High universality, low power input	Low degree of order	^362–385^
Nanofabrication-assisted colloidal self-assembly	Laser etching		Patterns	Designable 2D morphology of colloidal self-assembly	Dependence on the equipment	^392^
	Photopolymerization		3D colloidal entities	Designable 3D morphology of colloidal self-assembly	Dependence on the equipment	^19^
	Nanoimprinting		Composite colloidal self-assembled structures	Functional colloidal structures	Requirements of the stamp	^122^,^130^
	Transfer printing		Composite colloidal self-assembled structures	Hierarchical colloidal structures	Requirements of the stamp	^395^
Self-assembly of anisotropic colloidal particles	Patchy colloidal particles	DNA pairs, chemical interaction	Colloidal superlattices	Programmable colloidal structures with molecule geometry	Lack of experimental demonstration	^90^,^111^,^127^,^397^,^342^
	Polyhedral inorganic colloidal particles	Depletion force, capillary force		Anisotropic molecular crystallinity and shape	Low stability	^107^,^403–424^ ,
	DNA origamis	DNA pairs		Programmable structures with molecular geometry	Unscalability	^70^,^110^,^425–431^
Nature- mimetic self-assembly	Self-assembly of cellulose-based materials	Capillary force	Colloidal crystal films, lines, dots	Chiral structures	Low degree of order	^449–469^
	Self-grown colloidal self-assembly		Microphase separation, molecular interaction	Self-generated colloidal building blocks, regulable colloidal structure formed after self-assembly	Requirements of the special materials	^477^,^478^,^481^
	Self-assembly of biomolecules	Hydrophobic force	0D-3D assemblies	Programmable and functional structures with various geometries	Low degree of order	^483–490^

### 5.1 3D Printing Colloidal Self-Assembly

Beyond microstructure, the macroscale architecture of colloidal self-assemblies offers an additional dimension for engineering functionality, opening promising avenues for future advances in optical, communication, and biophotonic applications.^277^ Colloidal self-assemblies with simple architectures, such as cylinders,^278^ and donuts,^279^ have been fabricated. More complex geometries require 3D printing techniques as they allow creating complex geometries with unparalleled flexibility.^280–282^ Using the 3D printing technique, photonic periodic structures have been constructed by two-photon polymerization lithography for materials that undergo localized photochemical reactions or fused deposition molding for thermoplastic materials.^54,283–288^ In the context of colloidal self-assemblies, direct ink writing (DIW) and digital light processing (DLP) are the most commonly employed 3D printing methods. However, fabricating 3D colloidal structures presents a significant challenge. It requires simultaneously controlling assembly across both the microscopic and macroscopic scales, while balancing the slow self-assembly kinetics of nanoparticles in the inks with the rapid solidification needed after printing. In this section, we will focus on direct ink writing and digital light processing strategies and discuss how the two strategies address the challenge.

#### 5.1.1 Direct Ink Writing

DIW is an automated injection molding technology that fabricates customized structures by extruding highly viscous materials directly onto substrates, which has been widely used to prepare colloidal crystal filaments,^128^ patterns,^212^ and 3D-architected colloidal assemblies.^282^ To maintain the 3D structures, the colloidal ink must quickly undergo a liquid-to-solid, which can be achieved by an aqueous colloidal ink containing a gel-forming copolymer (Figure 16a).^289^ The ink can quickly transition from liquid to solid when heated, preserving the microarchitecture of the printed structure. Nanoparticles in the ink pack into quasi-periodic structures with long-range disorder and short-range order, endowing the printing objects with isotropic structural colors. Another method to achieve rapid liquid-to-solid transitions is to use Bingham inks, which solidify under low shear force but become liquid under high shear force. These inks can be created by designing the interactions between the colloidal nanoparticles and the matrices. For example, the solvation layer of silica nanoparticles in an acrylate resin can be broken by adding ethanol to the colloid-resin mixture, enabling van der Waals attraction to surpass the repulsion. As a result, the ink forms a colloidal network with high density and evenly distributed linkage strength when shear is absent. This network can be broken under shear flow and immediately transitions from liquid to solid after extrusion from the nozzle, allowing the creation of 3D structures (Figure 16b).^290^

**Figure 16:**
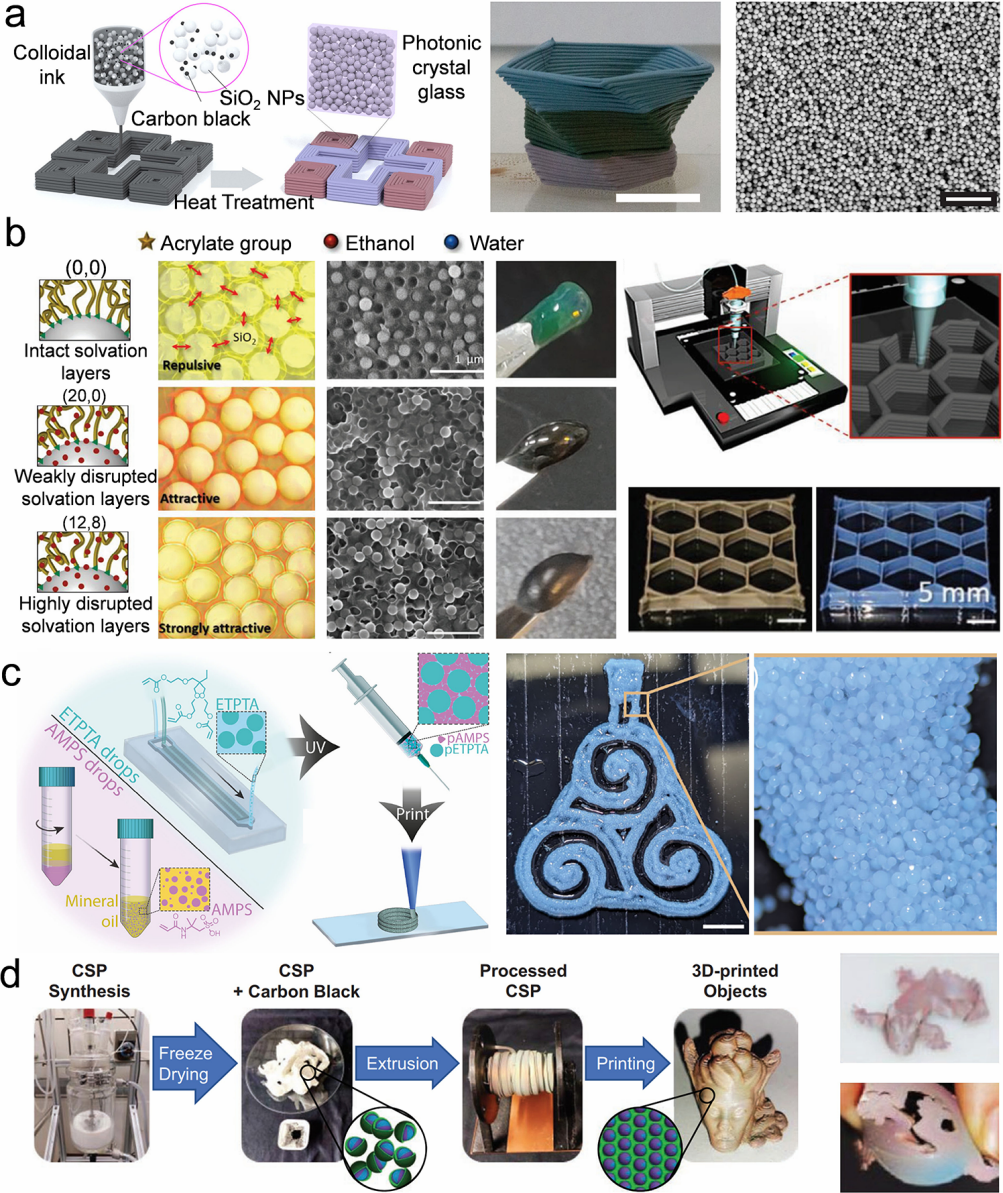
**3D printing of colloidal assembly based on direct ink writing (DIW).** a) Illustration of the DIW printing of colloidal inks and obtained objects with isotropic structural color. The ink swiftly achieves a liquid-to-solid transition by heating after being extruded, maintaining the obtained structure. Reproduced with permission from ref 289. Copyright 2022 Springer Nature under CC BY 4.0 (https://creativecommons.org/licenses/by/4.0/). b) DIW printing of a Bingham ink composed of monodispersed colloidal nanoparticles and acrylate-based resin. The interaction between the colloids and the matrix facilitates the formation of a colloidal network at an ultrahigh fraction, allowing for a swift liquid-to-solid transition of the ink after being extruded from the nozzle. Reproduced with permission from ref 290. Copyright 2023 Wiley-VCH. c) DIW printing of a mixture of rigid nanoparticles and soft microgel. The microgel improves the rheological properties of the ink, making it easy to extrude the ink. The addition of microgel enhances the mechanical properties of the printed object, as the microgel can cross-link into a network. Reproduced with permission from ref 291. Copyright 2025 Wiley-VCH under CC BY 4.0 (https://creativecommons.org/licenses/by/4.0/). d) DIW printing of an ink made of colloidal nanoparticles with a hard polystyrene core and a soft polyalkylacrylate-based shell. The soft shell facilitates the ordered arrangement of nanoparticles and endows the printed objects with mechanochromic sensing ability. Reproduced with permission from ref 280. Copyright 2025 Wiley-VCH.

Current colloidal inks usually contain rigid nanoparticles, such as silica nanoparticles. These particles are too rigid to yield, which prevents their extrusion by DIW, particularly for the ink with a high-volume fraction of silica nanoparticles. To address this issue, one strategy is to mix soft hydrogel microparticles with the rigid particles to modify the rheological properties of the ink, enhancing its shear-thinning and self-curing capabilities (Figure 16c).^291^ The printed colloidal self-assembly exhibits enhanced mechanical properties, as confirmed by the formation of a gel network by the hydrogel in the ink. Another approach involves creating core–shell nanoparticles with a rigid core and a soft shell, such as hard polystyrene cores and soft polyalkylacrylate shells (Figure 16d).^280^ The hard core offers mechanical support, while the soft shell is malleable when subjected to shear force, enabling an orderly assembly of nanoparticles. Upon heating, the printed structures are preserved effectively. The resultant objects exhibit mechanochromic properties owing to their core–shell architecture, rendering them promising candidates for smart coatings and advanced security devices.

#### 5.1.2 Digital Light Processing

Digital light processing (DLP) utilizes projected light to polymerize a precursor layer-by-layer, creating a 3D structure. Compared with the DIW technique, DLP has higher resolution (Figure 17a),^292^ and higher polymerization efficiency, making it suitable for creating more complex structures, such as hollow 3D objects. Typically, DLP inks contain colloidal nanoparticles, photocurable monomers, photoinitiators, photoabsorbers, and radical inhibitors. However, the resulting objects usually display a short-range order and long-range disorder in nanoparticle arrangement. To enhance the periodicity of 3D printed colloidal structures, nanoparticles should be evenly dispersed within the printable matrix during and after printing. This can be accomplished by increasing the surface charges on the colloidal nanoparticles (Figure 17b).^293^ Nanoparticles possessing high charges facilitate the development of a highly organized structure within the pregel solution, attributable to the repulsive interparticle electrostatic force. The force sustains the ordered structure after extrusion and polymerization of the pregel solution through the DLP technique. The resultant printed objects exhibit angle-dependent structural coloration, indicative of well-crystallized colloidal nanoparticles. Despite an ordered nanoparticle packing lattice and programmable morphologies, the objects show a step-like surface (step effect) attributed to the layer-by-layer fabrication process. The continuous liquid film-confined 3D printing strategy diminishes the step effect, achieving the manufacture of architected colloidal self-assemblies with smooth surfaces (Figure 17c).^294,295^ Nanoparticles modified with carboxyl groups (−COOH) disperse stably in a UV-curable matrix due to hydrogen bonds formed between the UV-curable monomer and −COOH. By controlling the confinement of the liquid–solid interface and using continuous printing mode, the liquid film adhering to the cured structure is drawn into the cured layer, while excess resin sticking to the cured structure is scraped away. This process reduces the step effect, resulting in objects with angle-dependent structural colors and smooth, step-free surfaces.

**Figure 17:**
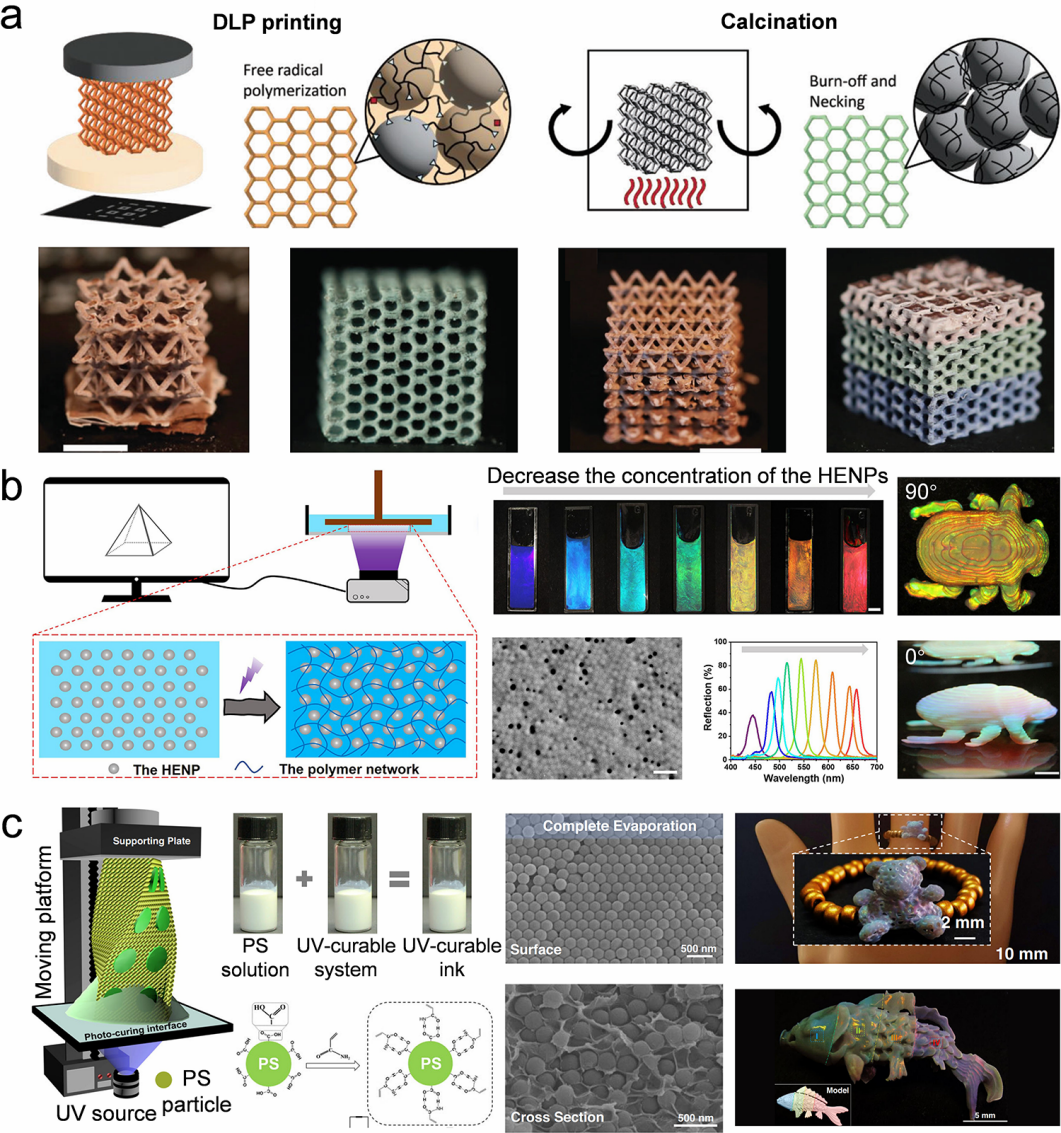
**3D printing of colloidal assembly based on digital-light processing.** a) Schematic showing digital light processing 3D printing and resultant objects with angle-independent structural colors, indicating the formation of short-range-order and long-range-disorder structures. Reproduced with permission from ref 292. Copyright 2023 Wiley-VCH. b) Schematic showing digital light processing 3D printing and resultant objects with angle-dependent structural colors, indicating the formation of ordered structures. The high charges on the nanoparticle surface facilitate the formation of the ordered structure. Reproduced with permission from ref 293. Copyright 2022 Springer Nature. c) Continuous digital light processing 3D printing apparatus for fabricating volumetric colloidal crystals without step-effect. The continuous printing mode diminishes the step effect. Reproduced with permission from ref 295. Copyright 2022 Springer Nature under CC BY 4.0 (https://creativecommons.org/licenses/by/4.0/).

#### 5.1.3 Summary

3D printing of colloidal self-assembly alters the 3D architecture of colloidal structures, adding an extra dimension for designing their functionality and applications. Extrusion-based printing methods are easy to operate but are limited by resolution, surface roughness, and ink options. DLP methods can create 3D colloidal self-assemblies with high resolution, smooth surfaces, and complex designs. Despite progress in this field, many challenges remain, such as defect control, ink design, microstructure quality, and microstructure regulation. Besides, other strategies have also been developed, including fused deposition molding and two-photon polymerication lithography. But they are used for thermoplastic materials and materials that undergo localized photochemical reactions. A summary of current 3D printing techniques is shown in Table 8.

**Table 8:** Summary of 3D Printing Colloidal Self-Assembly

Self-assembly strategies	Key parameters	Products	Remarks
Inkjet printing	Colloidal concentration, substrate wettability, and droplet components.	3D colloidal dots.	Tens of minutes to hours, limited shape variety.
Microfluidics	Colloidal concentration, nanoparticle wettability.	Donuts.	Tens of minutes to hours, limited shape variety.
DIW	Colloidal concentration, the rigidness of colloidal nanoparticles, the rheological properties of inks, nozzle size, printing speed, charges on colloidal surface, and interaction between nanoparticles and solvents.	3D-architected colloidal self-assembly, filaments.	Tens of minutes to hours, low resolution, step-effect surface.
DLP	Light intensity, colloidal concentration, charges on colloidal surface, interparticle interaction, interaction between nanoparticles and solvent, platform wettability, and colloidal wettability.	3D-architected colloidal self-assembly.	Tens of minutes to hours, low resolution.

### 5.2 Interfacial Colloidal Assembly

While evaporative self-assembly of colloidal suspensions and droplets typically yields multilayer (3D) structures. Many applications, such as surface modification,^94^ total reflection,^134^ and optoelectronics^30,296^ require a monolayer (2D). Interfacial self-assembly at air–liquid or liquid–liquid interfaces provides an effective route to such 2D structures, driven by capillary, solvation, and electrostatic forces. In this section, we will introduce several strategies based on the air–liquid and liquid–liquid interface to assemble monolayer colloidal assemblies.

#### 5.2.1 Air–Liquid Interface

The Langmuir–Blodgett (LB) technique is an example of air–liquid interfacial self-assembly, which involves floating amphiphilic molecules on top of an aqueous subphase and compressing the molecules into a dense monolayer and transferring the monolayer to a solid substrate (Figure 18a).^297–299^ With the development of this technique, Langmuir monolayers have been extended to the macrocyclic molecules,^300^ ionic liquids,^301^ carbon-based nanomaterials,^302^ and metal nanoparticles.^299,303^ When assembling metal nanoparticles in a Langmuir trough, nanoparticles spread in the aqueous subphase and then assemble into small crystal domains. The small domains merge into a continuous film using two barriers to compress the domains (Figure 18b).^304^ The LB technique requires hydrophobic or amphiphilic nanoparticles. The obtained film is determined by the interaction between the colloidal nanoparticles, between the nanoparticles and the solvent, and the core–ligand size ratio. Except for the films, the LB transfer process can be used to form patterns from a homogeneous Langmuir monolayer by controlling the dynamic behaviors of the three-phase contact line.^53^ Similar to the LB method, an air–liquid interface assembly technique is created: Hydrophobic PS nanoparticles suspended in an ethanol–water mixture are dripped onto an air–water interface. The ethanol reduces the surface tension, encouraging the hydrophobic PS nanospheres to spread on the air–liquid interface and form a monolayer. This monolayer film can then be transferred to a hydrophilic substrate.^123^ Despite the convenience of preparing various derivative structures, including multilayer colloidal films^305–308^ and colloidal crystal patterns,^309^ this approach is not ideal for assembling hydrophilic particles or particles that are denser than water, since they cannot float at the liquid surface very well. To address this issue, a universal and conformal thin film technique that utilizes wetting-empowered interfacial self-assembly is proposed (Figure 18c).^310^ The technique entails the addition of a perfluoro sulfhydryl ligand [perfluoro decanethiol (PFT)] into a colloidal suspension. Upon stirring, the wettability of the hydrophilic nanoparticles is altered through the bonding of hydrophobic PFT. Subsequently, the colloidal particles migrate to the air–liquid interface from the bulk solution, assembling into monolayer films. The produced monolayer colloidal films can be applied as coatings on various substrates, including plastics and paper. This approach enhances the versatility of air–liquid interfacial self-assembly; however, it is primarily limited to film fabrication.

**Figure 18:**
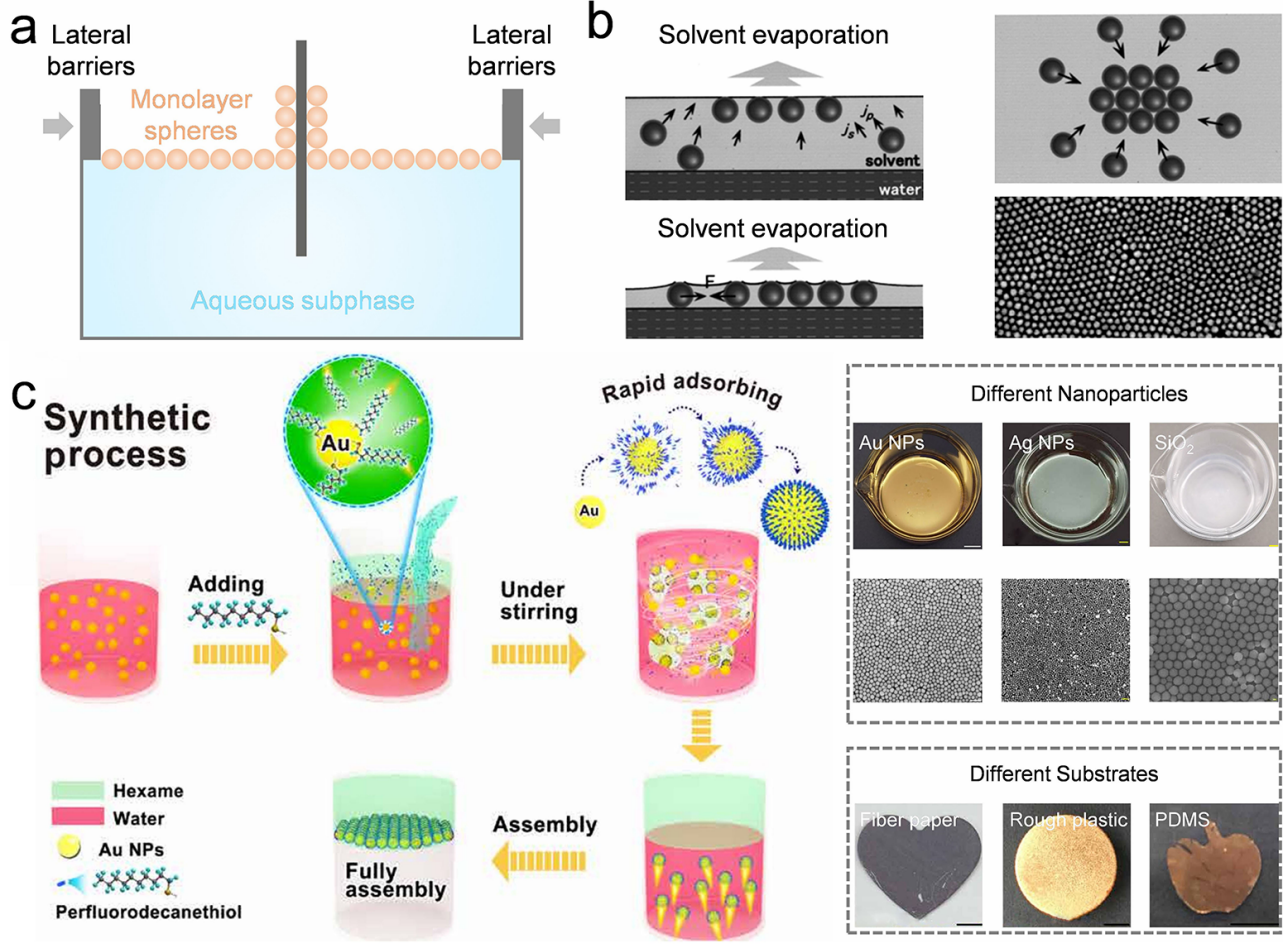
**Air–liquid interfacialself-assembly of monolayer colloidal films.** a) Schematic illustration of Langmuir compression. b) Mechanism of the self-assembly of Langmuir monolayer: The process starts with convective assembly of nanoparticles at the air–liquid interface. With the evaporation of solvent, interparticle attraction increases, leading to the formation of a closely packed monolayer nanoparticle array. Reproduced with permission from ref 304. Copyright 2001 American Vacuum Society. c) Schematic illustration of the rapid fabrication of scale-up and density assembly of nanoparticles at the interface. Reproduced with permission from ref 310. Copyright 2021 The American Association for the Advancement of Science.

To create 2D colloidal crystal pixels, the evaporation of colloidal droplets has been used. Although monolayer nanoparticle arrays have been achieved through directly evaporating colloidal droplets,^311–313^ this approach requires precise control of the particle concentration, solvent components, and suppression of the coffee-ring effect. To simplify the fabrication of 2D monolayer colloidal crystal pixels, several strategies have been developed. For example, well-assembled monolayer nanoparticles can be fabricated by controlling the evaporation kinetics of a colloidal droplet (Figure 19a).^311^ This approach enables the formation of monolayer colloidal pixels and scalable films due to two main factors: Quick droplet evaporation, which separates particles at the air–liquid interface, and an attractive force between particles and the air–liquid interface, drawing nanoparticles to the surface. Additionally, a dual-droplet inkjet printing method has been developed to produce monolayer colloidal pixels. This involves dispensing a supporting droplet with high surface tension onto a substrate, then adding a wetting droplet with low surface tension on top of it. (Figure 19b).^314^ During the drying process of the supporting droplet, the colloidal nanoparticles within the wetting droplet disperse across the surface of the supporting droplet and assemble at the interface as the solvent evaporates, resulting in a uniform and monolayer deposition. The coffee-ring effect is mitigated by the Marangoni flow induced by the surface tension gradient between the two liquids. This methodology offers an approach to fabricate monolayer colloidal assembly pixels for applications that necessitate high-resolution or patterned monolayer colloidal assemblies.

**Figure 19:**
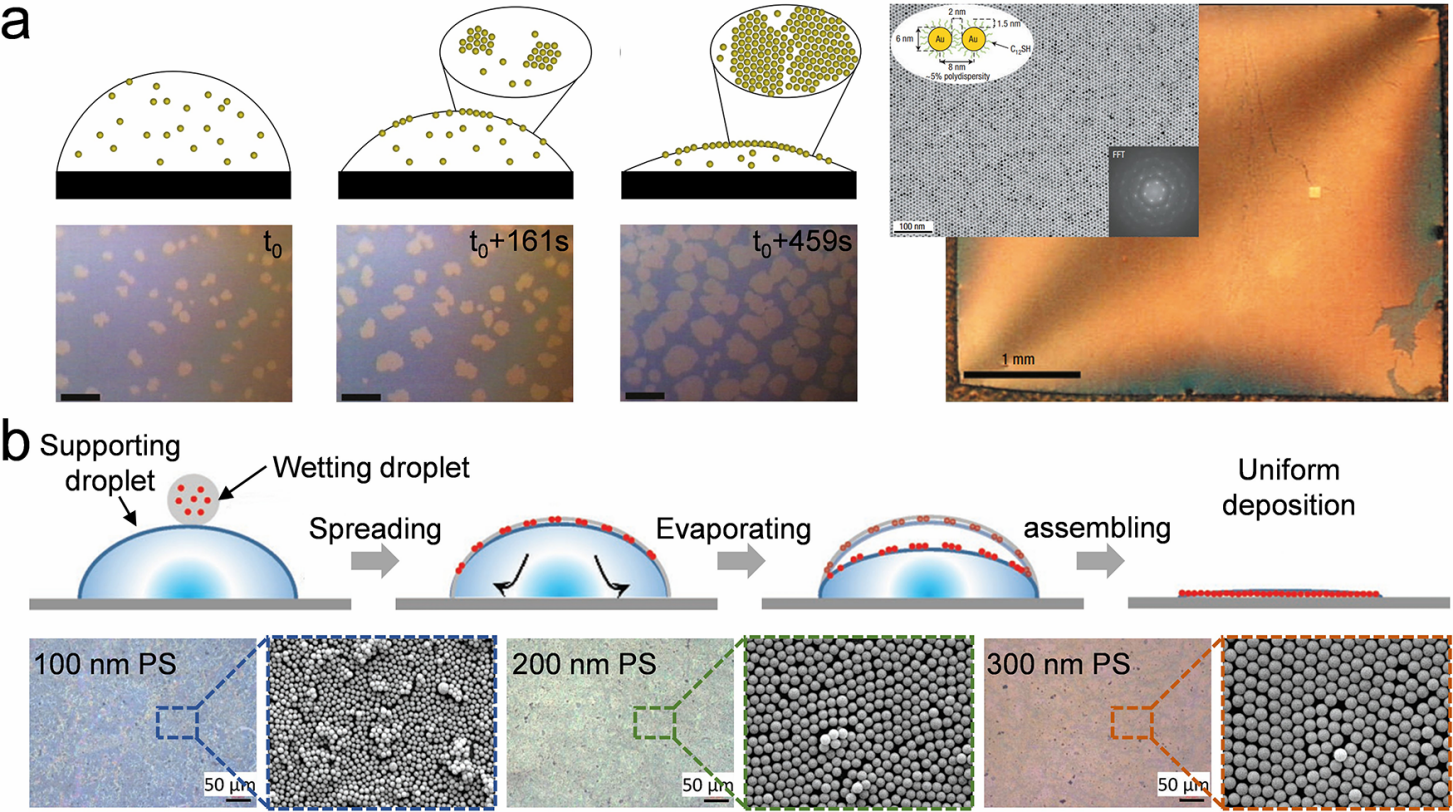
**Air–liquid interfacial self-assembly of monolayer colloidal dots.** a) Schematic illustration of the kinetically driven self-assembly of nanoparticle monolayers. Due to the fast evaporation of the solution and attractive interaction between the nanoparticle and the air–liquid interface, Au nanoparticles well-assemble into an ordered monolayer structure. Reproduced with permission from ref 311. Copyright 2006 Nature Publishing. b) Schematic illustration of dual-droplet printing to assemble colloidal nanoparticles at the droplet surface and SEM images of PS nanoparticle films assembled by dual-droplet printing. Reproduced with permission from ref 314. Copyright 2018 Wiley-VCH.

#### 5.2.2 Liquid–Liquid Interface

When two immiscible liquids are mixed, the liquid–liquid interface can accumulate and assemble nanoparticles due to the interfacial forces formed (Figure 20a).^216^ For instance, Au nanoparticles assemble into a liquid mirror with high reflectance and electrical conductivity at the [heptane+DCE]/water interface (Figure 20b).^315^ The assembling process is influenced by the wettability^316^ and the surface charge density of the colloids.^317^ In addition to the 2D films, nanoparticles can organize into 3D structures, such as colloidosomes, at the interface of two immiscible liquids, which cannot be synthesized via an air–liquid interface. Colloidosomes are microcapsules characterized by an elastic shell composed of densely packed particles that are stabilized through electrostatic forces or van der Waals forces.^318^ Pickering emulsion is a widely used model for creating colloidosomes, because nanoparticles can adsorb at the liquid–liquid interface to stabilize the emulsion (Figure 20c).^318^ The adsorption process pertains to the surface characteristics of colloidal nanoparticles and the interfacial tension of the double emulsion. Generally, a high interfacial tension in the double emulsion promotes the assembly of colloidosomes. In addition to water/oil emulsions, all aqueous phase separation systems have been utilized to assemble nanoparticles and to engineer the resulting structures. All aqueous phase separation systems are composed of the phase separation of an aqueous mixture containing two or more incompatible additives, such as incompatible polymers or a combination of polymers and salts. The interfacial tension between the immiscible aqueous phases is lower than that observed in water/oil systems, facilitating the manipulation of their interfacial behaviors. Beyond traditional spherical emulsion droplets, flower-shaped droplets with varying numbers of petals are produced through the self-emulsification of a Na_2_CO_3_–PEG-based aqueous two-phase system, which absorbs nanoparticles at the aqueous interface to form a dense, flower-shaped colloidal structure (Figure 20d).^319^ Furthermore, multilevel hierarchical structures can be developed through multilevel compartmentalization facilitated by interfacial separation within an aqueous two-phase system, resulting in the formation of a three-dimensional sheet of subcolloidosomes encapsulating a mother colloidosome (Figure 20e).^320^ However, the low interfacial tension between the two aqueous phases makes it hard to maintain the obtained assemblies. Thus, oppositely charged polyelectrolytes are used to facilitate the assembly of nanoparticles at the hierarchical aqueous interface to form a coacervate-nanoparticle-composite network. Because the electrostatic force between the oppositely charged nanoparticles can enhance the bonding between the colloids, leaving a tough structure (Figure 20e). The self-assembly of nanoparticles at the liquid–liquid interfaces further enhances the design and fabrication of hierarchical structures.^214–216^

**Figure 20:**
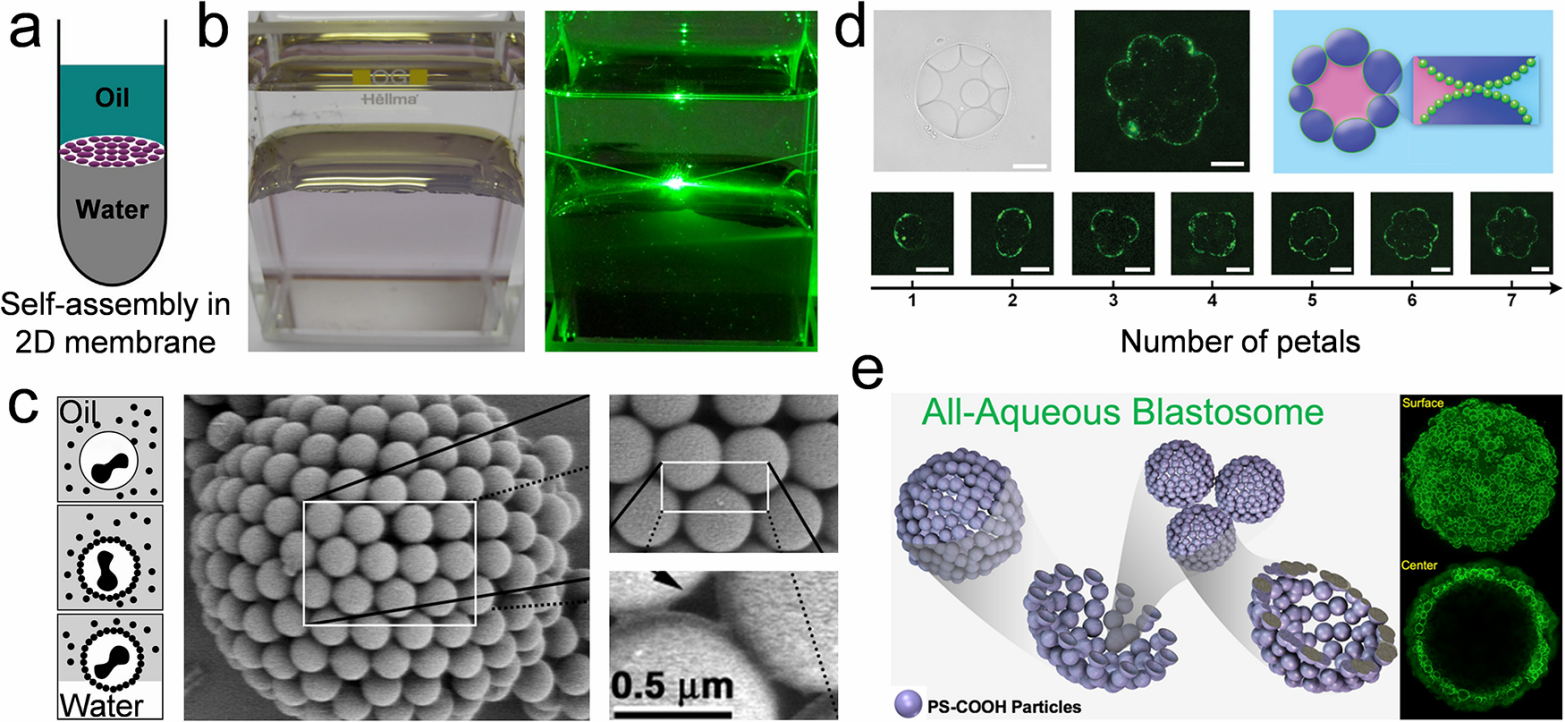
**Liquid–liquid interfacial colloidal self-assembly.** a) Schematic presentation of the self-assembly of nanoparticles into a 2D membrane at a liquid–liquid interface. Due to interfacial forces, colloidal nanoparticles assemble at the interface, which is determined by the surface wettability and charges. Reproduced with permission from ref 216. Copyright 2019 Wiley-VCH under CC BY 4.0 (https://creativecommons.org/licenses/by/4.0/). b) Optical images of an Au nanoparticle film prepared at the [heptane+DCE]/water interface. Reproduced with permission from ref 315. Copyright 2013 American Chemical Society. c) Schematic illustration of the self-assembly process for colloidosomes and SEM images of the corresponding samples. Reproduced with permission from ref 318. Copyright 2002 The American Association for the Advancement of Science. d) Nanoparticles assembled at the surface of flower-shaped droplets. The flower-shaped droplets are formed in a Na_2_CO_3_–PEG-based aqueous two-phase system. Reproduced with permission from ref 319. Copyright 2020 Royal Society of Chemistry. e) Schematic diagram of structures for the bare blastosome and the corresponding fluorescent images of samples. Oppositely charged polyelectrolytes and PS nanoparticles assemble in a PEG-DEX-based ATPS, where the phase separation leads to the multilevel compartmentalization at the interface of the PEG-rich phase and the DEX-rich phase. Reproduced with permission from ref 320. Copyright 2020 American Chemical Society.

#### 5.2.3 Summary

Driven by interfacial forces, various building blocks, such as the nanorods,^321^ nanoparticles,^123^ and nanopolyhydra,^322^ assemble at the air–liquid or liquid–liquid interface to form monolayer colloidal assemblies. The mobility of the building blocks at the interface facilitates a homogeneous interface coverage. However, it increases the risk of generating cracks and makes it difficult to shape the obtained structures. The interfacial self-assembly of colloidal nanoparticles is not suitable for preparing patterns because colloidal building blocks tend to spread across the interface. Engineering of the all-aqueous liquid–liquid interface provides a strategy to prepare 2D or 3D colloidal self-assemblies with various structures. However, maintenance of the obtained structures is a challenge due to the low surface tension between the two immiscible aqueous phases. General interfacial colloidal self-assembly strategies are summarized in Table 9.

**Table 9:** Summary of Interfacial Colloidal Self-Assembly

Self-assembly strategies	Key parameters	Outcomes	Remarks
Langmuir–Blodgett	Amphiphilic or hydrophobic colloidal nanoparticles, monodispersity of nanoparticles, particle interaction, interaction between the colloidal nanoparticles and the dispersed solvent, and the size ratio of core and capping ligands.	Monolayer or multilayer colloidal assemblies.	Tens of minutes to hours, film size from several to tens of cm^2^, experimentally complex.
Dispensing air–liquid interfacial self-assembly	Hydrophobic nanoparticles, smaller density of colloids than that of water, smaller surface tension of colloidal suspension than that of water.	Films or patterns of monolayer colloidal assemblies.	Tens of minutes to hours, film size from several to tens of cm^2^, experimentally complex.
Wetting-empowered interfacial self-assembly	Hydrophilic nanoparticles, the content of hydrophobic PFT, and the concentration of nanoparticles.	Films or patterns of monolayer colloidal assemblies.	Several to tens of minutes, outcome size from several to tens of cm^2^.
Dual-droplet inkjet printing	Higher surface tension of the supporting droplet than that of the wetting droplet, and colloidal concentration.	Pixels of monolayer colloidal assemblies.	Several minutes, outcome size around tens of cm^2^.
Oil–water interfacial self-assembly	Wettability and surface charge density of colloidal nanoparticles, stabilization of oil–water emulsions.	Films of monolayer colloidal assemblies, colloidosomes.	Tens of minutes to hours, outcome size around tens of cm^2^.
All-aqueous interfacial self-assembly	Stabilization of the all-aqueous interface.	Colloidosomes with heterogeneous structure or different morphologies.	Tens of minutes to hours, outcome size around tens of cm^2^.

### 5.3 External Field-Directed Colloidal Assembly

Strategies mentioned above rely primarily on forces intrinsic to the colloidal system, including capillary, shear, and confinement forces. These forces are inherently difficult to control precisely, resulting in limited control over the dynamic assembly process. To achieve higher precision, external stimuli have been introduced to direct the colloidal self-assembly. Under such stimuli, responsive colloidal building blocks can organize into specific structures in a highly controllable manner. This significantly expands the structural and functional diversity of the assemblies, facilitating progress toward commercialization, scalability, and multifunctionality. In this section, we will use field-directed assembly as an example to introduce stimuli-responsive systems for fabricating lines, patterns, and films.^25,46,84,251,323^

Another reason we choose external-field-directed colloidal self-assembly is that it is a typical example of dynamic colloidal self-assembly.^47,54^ Most strategies discussed previously fall under equilibrium self-assembly, where systems evolve toward a stable, ordered structure determined by thermodynamic parameters and persist without continuous energy input. In contrast, dynamic self-assembly refers to systems maintained in a nonequilibrium state through continuous energy input, such as that provided by external fields.^324,325^ Similar systems include chemical potential,^326,327^ microfluidics,^328^ active matter,^329,330^ and biomolecules.^331–333^

#### 5.3.1 Electrical Field

The electric field-directed colloidal assembly has garnered interest owing to the ease of programming electric stimuli and the capability to construct microelectrode arrays or patterned electrodes on various substrates, thereby facilitating the pixelation and patterning of the resulting assemblies. Consequently, electrical fields have been employed in commercial products to induce colloidal self-assembly, such as in E-book readers, wristwatches, electronic tags, and similar devices. The electrical manipulation of colloids primarily arises from the electrostatic force acting on charged particles, known as electrophoresis (Figure 21a).^84^ In a polar dispersion medium, such as propylene carbonate (PC), charged colloidal nanoparticles create polarized electric double layers under an electric field, enabling colloids to migrate toward the hydrophilic electrode due to the electrical forces and achieving dynamic self-assembly.^84^ In commercial electrophoretic inks, nanoparticles with opposite charges that strongly scatter and absorb light dynamically assemble at the display’s front side under an electric field, creating white or black pixels. To produce colored pixels, nanoparticles need high optical contrast, fast electrophoretic mobility, and stability. Inorganic metal-oxide nanoparticles are a good choice and can spontaneously form quasi-amorphous structures when their concentration exceeds a critical threshold (Figure 21b). The lattice constant of these structures can vary depending on the applied electric fields.^334^

**Figure 21:**
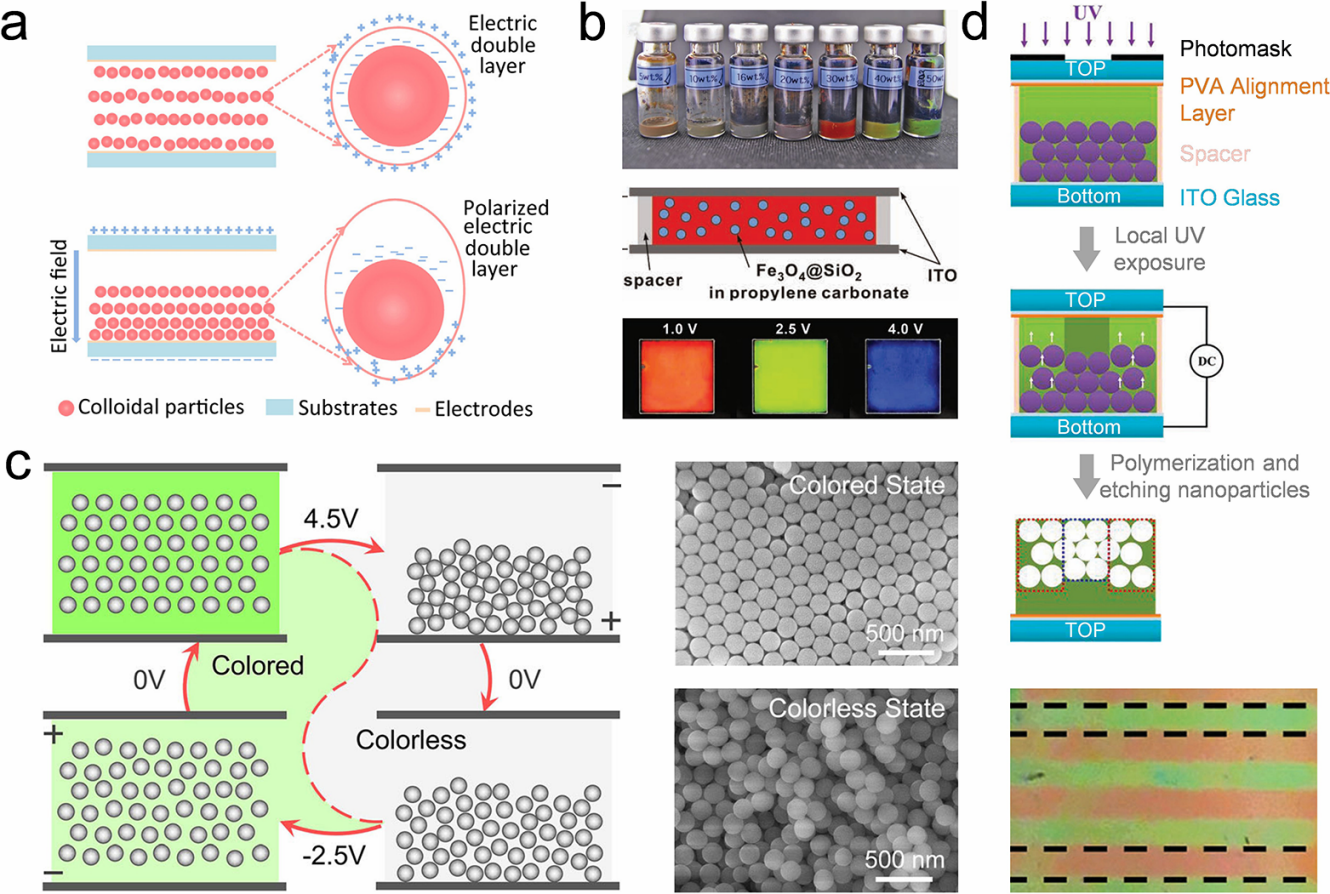
**Electric field-directed colloidal self-assembly.** a) Schematic of the electrophoretic organization of the colloidal systems driven by an electric field. The polarized electric double layer under an electric field generates a force to assemble nanoparticles at the surface of the electrode. Reproduced with permission from ref 84. Copyright 2018 Elsevier. b) Quasi-amorphous colloidal glass with electrically tunable lattice constants determined by the strength of the electric field. Reproduced with permission from ref 334. Copyright 2010 Wiley-VCH. c) Changes in the structure of the assembled colloidal particles between the ordered state and disordered state triggered by an electric field. The ordered structure is maintained due to the high viscosity of PC–PEG-EG-based ink and collapses under a reversed electric field. Reproduced with permission from ref 335. Copyright 2022 Springer Nature under CC BY 4.0 (https://creativecommons.org/licenses/by/4.0/). d) Patternable colloidal structures facilitated by the electric field-induced heterogeneous colloidal self-assembly. Colloidal nanoparticles are mixed with UV-curable resin, which is used to maintain the assembled structure. Reproduced with permission from ref 336. Copyright 2015 Royal Society of Chemistry.

Under an electric field, highly charged colloids facilitate the formation of an ordered organization of nanoparticles.^95^ However, maintaining the assembled structure requires a continuously applied electrical force. Once the field is removed, the structure disintegrates due to electrostatic repulsion among particles and the stochastic Brownian motion. To address this issue, an electric-responsive ink composed of negatively charged colloidal nanoparticles, PC, PEG, and ethylene glycol (EG) is designed.^335^ PEG and EG impart a high viscosity to the ink. Consequently, the ink can maintain the colloidal structure after the electric field is switched off. In the field-off state, the highly charged nanoparticles self-organize through electrostatic interactions, stabilized by the elevated viscosity, producing vibrant structural colors. Conversely, applying a reverse electric field drives the nanoparticle toward the positive electrodes, disrupting the ordered structure and resulting in a colorless state. The nanoparticles can be redispersed uniformly by applying an inverse electric field (Figure 21c). In addition to augmenting the viscosity of the ink matrix, the self-assembled structures induced by the electric field can be stabilized through photopolymerization techniques. Within a mixture comprising colloidal nanoparticles and UV-curable resin, the electrically induced ordered structure is preserved by subjecting the mixture to ultraviolet light exposure. Consequently, a heterogeneous colloidal assembly with varying lattice constants can be fabricated and secured by alternately applying an electric field and localized UV irradiation utilizing a mask (Figure 21d).^336^ Apart from the electrical responsiveness, the resulting colloidal assemblies exhibit unique properties when utilizing functional materials as the photopolymerizable filling matrix.

#### 5.3.2 Magnetic Field

The magnetic field serves as an alternative to the electric field for inducing the dynamic self-assembly of colloids.^337–339^ Superparamagnetic nanoparticles align into ordered structures under the applied field, and the assemblies are maintained only as long as the magnetic field is present. The attractive magnetic force from the superparamagnetic core of nanoparticles is balanced with repulsive electrostatic and solvation forces. The competitive result of the attractive and repulsive forces determines the interparticle distance of the resultant structure. A stronger magnetic field leads to a denser structure. Besides the continuous input of a magnetic field, the obtained structure can be fixed with a polymerized resin (Figure 22a).^323^ Compared to the electrical field, an advantage of the magnetic field-induced colloidal self-assembly is that magnetic building blocks possess a magnetic dipole. Therefore, the orientation of magnetic colloidal building blocks is responsive to the magnetic field direction, especially for anisotropic building blocks such as nanorods. Due to their anisotropic morphology, the resultant assemblies exhibit multiple colors through a single photonic pigment by adjusting the magnetic-responsive orientation of the crystal domains (Figure 22b).^340^ An additional benefit of magnetic building blocks is that the magnetic materials can be coupled with other functional materials via chemical synthesis.^341^ For instance, hybrid nanorods of Fe_3_O_4_/Au, integrating magnetic and plasmonic properties, are synthesized. These nanorods assemble into ordered structures with controllable orientations under a magnetic field at specific locations within polymer matrices. The resulting polymer films exhibit outstanding mechano-responsive structural colors in response to linear rotation, bending, and nonlinear twisting (Figure 22c).^342^ These self-assembled entities are endowed with various functions by adjusting the shape of magnetically coupled building blocks, such as chiroptical responses^343^ or angle-dependent plasmonic scattering,^344^ expanding the possible applications of magnetically induced self-assemblies.

**Figure 22:**
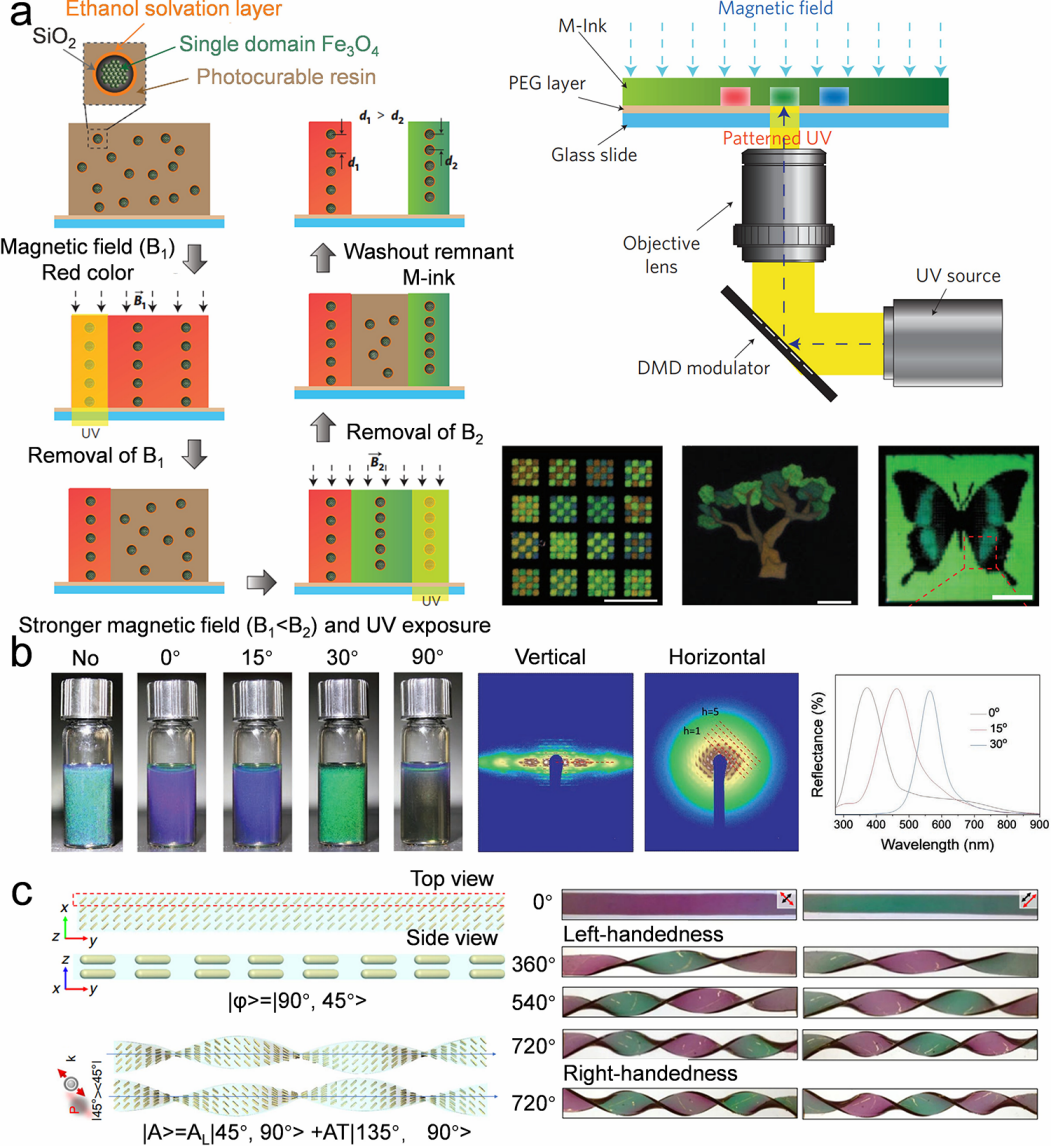
**Magnetic field-directed colloidal self-assembly.** a) Schematic of constructing a heterogeneous magnetic colloidal structure with high resolution by a magnetic field. Reproduced with permission from ref 323. Copyright 2009 Springer Nature. b) Colloidal self-assembly consisting of magnetic nanorods with various plane orientations aligned by directional magnetic fields. Reproduced with permission from ref 340. Copyright 2022 Wiley-VCH. c) Magnetic-induced specific arrangement of Au nanorods in a plasmonic film. Reproduced with permission from ref 342. Copyright 2020 Springer Nature under CC BY 4.0 (https://creativecommons.org/licenses/by/4.0/).

#### 5.3.3 Light Field

The light field is another promising tool to program the dynamic self-assembly of colloidal nanoparticles in a precise way by adjusting the optical intensity,^345^ polarization,^346^ phase,^347^ shapes,^348^ and other control parameters.^349,350^ A focused laser beam is capable of exerting a force of dozens of piconewtons on colloidal particles, enabling manipulation of their position and assembly. This phenomenon is referred to as optical trapping tweezers.^351^ This force depends on interparticle electromagnetic interactions facilitated through light scattering. The optical gradient force includes two components: (i) The intensity gradient force, which involves colloidal constituents migrating toward regions of higher light intensity, and (ii) the phase gradient force, which promotes migration along the phase gradient. The equilibrium between these two forces is contingent upon the dielectric properties of the particle.^347^ When the two forces act in concert with each other, light-responsive colloidal building blocks become kinetically trapped and aligned at a relative equilibrium point. If the forces oppose one another, this ordered alignment is lost (Figure 23a).^352^ Nevertheless, the operational range of the optical force remains restricted, rendering it difficult to assemble larger nanoparticles. Furthermore, the structure tends to disintegrate once the light field ceases, since the structure is in a nonequilibrium state. To make the structure in an equilibrium state, it is necessary to exert a sufficiently strong light-induced force on the colloidal particles, facilitating their close packing to enable the van der Waals force. To overcome the interaction barrier associated with the reducing interparticle distance, the external effect brought by light field, such as optothermal effects^353,354^ and dielectrophoretic migration of colloidal particles,^355^ have been employed. The triggers can be generated by femtosecond laser-directed bubble microprinting (FsLDBM) (Figure 23b).^356^ When irradiating a femtosecond laser beam onto a solvent–substrate interface, the solvent is locally heated due to the multiphoton absorption and evaporates, forming microbubbles. The heat also generates a thermo-induced Marangoni flow, gathering colloidal nanoparticles near the laser point. Microbubbles capture suspended particles due to the evaporation flow, and the capillary force causes the nanoparticles to adhere to the substrate. The obtained structures can be well-maintained due to the van der Waals force when the laser is turned off. FsLDBM eliminates the requirements of substrates and target colloidal building blocks in the conventional microbubble printing technique, achieving high-resolution printing of various materials. The light-controlled temperature field can also dynamically assemble colloidal nanoparticles into 2D or 3D geometries through opto-thermophoretic assembly (OTA) (Figure 23c). In a typical system, an Au film works as an opto-thermoresponsive substrate. A surfactant (e.g., cetyltrimethylammonium chloride, CTAC) is added to a colloidal suspension as a surface charge source, macro ion, and a micellar depletant.^357^ Upon laser irradiation, the induced temperature gradient leads to the splitting of CTAC micellar and Cl^–^, generating a light-induced thermoelectric field. The field drives the colloidal nanoparticles to migrate along the temperature gradient. Subsequently, the nanoparticles assemble into light-responsive 1D and 2D structures, facilitated by depletion forces. Except for the single bunch of light, a standing wave pattern can be created to enable multiple light-particle interaction sites, improving the efficiency of the self-assembly process.^358^

**Figure 23:**
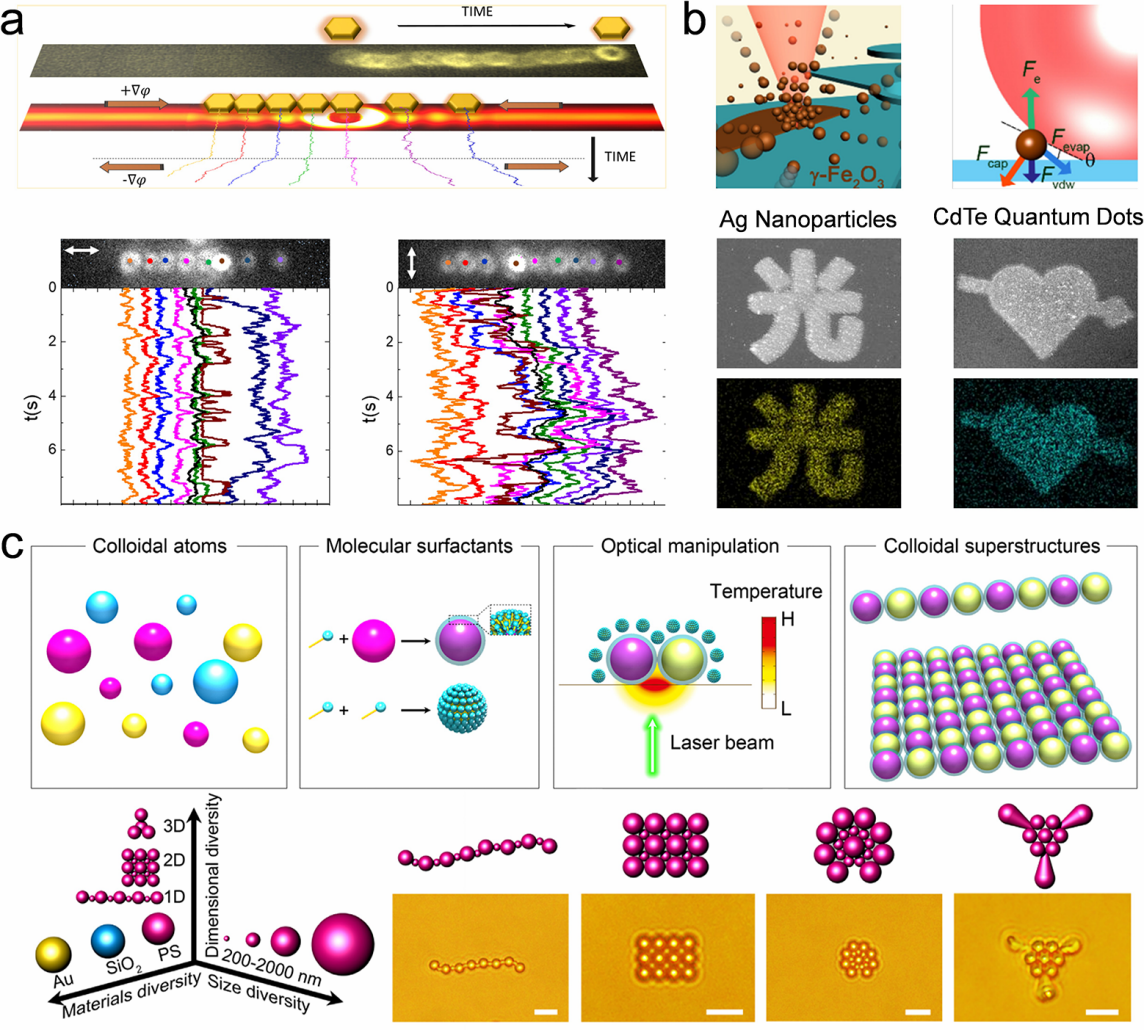
**Light field-directed colloidal self-assembly.** a) Nanoplatelets aligned by optical fields and their position change after being trapped. Optical intensity gradient force and phase gradient force synergistically program the assembly process. Reproduced with permission from ref 352. Copyright 2018 American Chemical Society. b) Assembled colloidal nanoparticles with the assistance of a femtosecond laser-directed bubble microprinting. Colloidal nanoparticles assemble along the bubble interface driven by harnessing a thermos-induced Marangoni flow, evaporation flow, and capillary flow. Reproduced with permission from ref 356. Copyright 2021 American Chemical Society. c) Programmable self-assembly of positive and hydrophilic colloidal nanoparticles by opto-thermophoretic assembly. The laser beam generates a dynamic temperature field, assembling colloidal nanoparticles into 1D or 2D superstructures. Reproduced with permission from ref 357. Copyright 2017 The American Association for the Advancement of Science.

Beyond optical tweezers, dynamic assembly of colloidal nanoparticles can also be achieved through light-particle interactions, which are facilitated by grafting photosensitive capping ligands onto the nanoparticles, allowing for light-induced phase segregation and the selective dynamic assembly of colloidal nanoparticles within a mixture. For instance, when a mixture of Au nanoparticles of varying sizes, each functionalized with two different azobenzene moieties, is exposed to UV light, the selective self-assembly of these functionalized nanoparticles can be triggered (Figure 24a).^359^ The processes of self-assembly and disassembly are driven by the light-induced isomerization of azobenzene. Irradiation with various wavelengths of light selectively assembles gold nanoparticles of differing sizes. This approach offers a strategy for dynamically segregating and assembling specific components within complex systems, which is typically challenging due to the nonspecific and short-range nature of colloidal-colloidal interactions that generate minimal segregation enthalpy. The light-induced chemical reaction also provides a potential solution. For instance, TiO_2_ nanoparticles coated with spectrally distinctive dyes (e.g., SQ2 and LEG4) can undergo phase separation under different illumination conditions (Figure 24b).^360^ The illumination directs the interparticle interaction through redox reactions in dyes. TiO_2_ nanoparticles combined with SQ2 are activated under red light irradiation, while those associated with LEG4 are activated under blue light irradiation. Light of varying wavelengths and intensities enables the controllable dynamic phase segregation of different nanoparticles. Based on this strategy, a dynamic, photochromic colloidal swarm is constructed by exposing a mixture of cyan, magenta, and yellow colloids to specific light sources. Besides the selective programming of specific nanoparticles within a mixture, the light field also offers a means to spatially regulate the self-assembly of colloidal nanoparticles, which can be achieved through the modification of the surface charge of nanoparticles via light (Figure 24c).^361^ Positively charged ZnO NPs are modified by sodium citrate to exhibit negative charges. The modified ZnO@Cit NPs are subsequently deposited onto a negatively charged substrate, followed by UV irradiation. The irradiation induces a change in the surface potential of ZnO NPs from negative to positive, thereby facilitating interparticle bonding. The resultant bonded patterns can be deposited onto the substrate owing to electrostatic attraction. Consequently, the light field offers a promising approach for the precise and selective programming of colloidal nanoparticle self-assembly.

**Figure 24:**
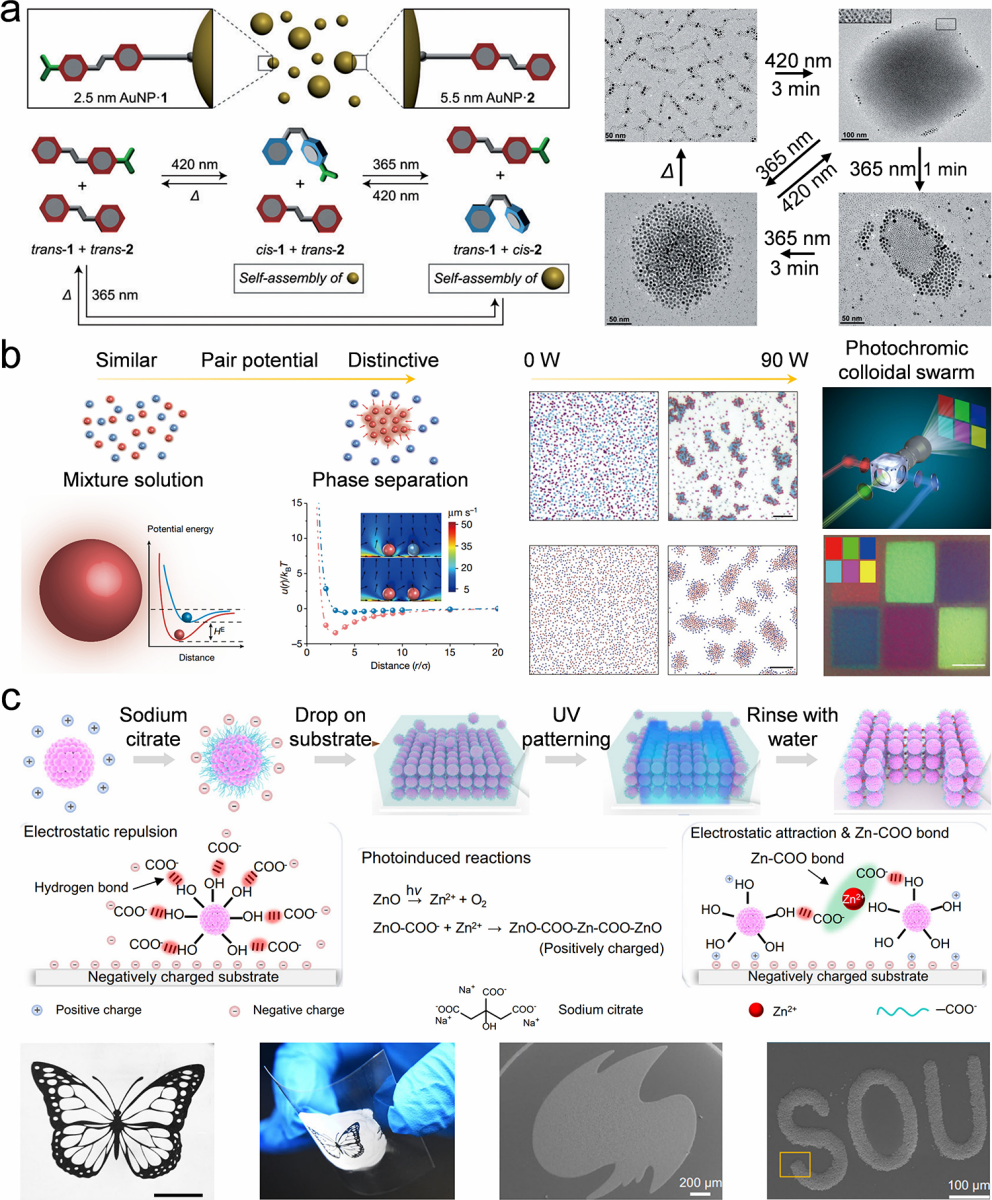
**Light field-directed colloidal self-assembly.** a) Light-programmed self-assembly in a mixture of 2.5 and 5.5 nm Au nanoparticles functionalized with photosensitive azobenzene molecules. Au NPs can assemble and disassemble when the mixture is exposed to light with different wavelengths due to the interaction between the light and azobenzene. Reproduced with permission from ref 359. Copyright 2015 Wiley-VCH. b) Light-induced phase segregation in a mixture of TiO_2_ colloidal species, which are modified by spectrally distinct dyes. The phase segregation depends on the interparticle potential that is adjustable by irradiating light with different wavelengths and intensities. Reproduced with permission from ref 360. Copyright 2023 Springer Nature under CC BY 4.0 (https://creativecommons.org/licenses/by/4.0/). c) Light-induced patternable ZnO@Cit colloidal self-assembly. Positively charged ZnO NPs change the surface potential by the irradiation of light, triggering the interparticle bonding. Reproduced with permission from ref 361. Copyright 2024 Springer Nature.

#### 5.3.4 Acoustic Field

An acoustic field can stimulate the dynamic self-assembly of colloidal nanoparticles, as the particles only need to differ in density or compressibility from their surroundings.^362^ Acoustic waves can manipulate particle dynamic assembly with a low power density (0.01 W/cm^2^).^363,364^ Acoustic fields are usually used to align,^365–367^ pattern,^368,369^ separate,^370,371^ and concentrate^372,373^ colloidal microparticles. Standing surface acoustic waves (SSAWs) can induce colloidal self-assembly. The waves oscillate in time, but their peak amplitude profile remains stationary. Thus, the fluid moves vertically at the antinode and horizontally around the antinode, accumulating colloids near the antinode (Figure 25a).^374^ Consequently, colloidal nanoparticles within a liquid film can be collected at the antinode of the standing wave through vibrating fluid motion, resulting in the formation of a crystal-like colloidal structure. This crystal-like colloidal arrangement can be maintained by continuously providing an acoustic field. To create a permanent microstructure, surface modification of the colloidal particles is required. For instance, Ag nanoparticles can assemble into a stable configuration by removing the polyacrylic capping on them (Figure 25b).^375^ Without protection from the polyacrylic capping, Ag nanoparticles sinter and form stable, conductive stripes after being directed toward the pressure nodes. The driving force stems from the positive acoustic contrast factor of Ag nanoparticles in water. Typically, SSAWs can generate periodic patterns due to the intrinsic nature of standing waves and the coupled vibrations between fluid and structure. To induce patternable colloidal self-assemblies, it is essential to consider a specialized device. For instance, a thin PDMS film containing air cavities can selectively suppress structural vibrations on the platform. Due to the disparate mechanical strengths of PDMS and air, nanoparticles tend to dynamically accumulate in nonperiodic potential wells, producing patterned colloidal self-assemblies with a line resolution of 50 μm (Figure 25c).^376^ In addition to the periodic and patternable colloidal assemblies, the acoustic field provides an effective method to manipulate colloidal nanoparticles precisely. By constructing a harmonic acoustics, such as the Fourier-synthesized harmonic waves, soft and reconfigurable acoustic lattices are created (Figure 25d).^377^ The morphologies, sizes, and positions of acoustic potential wells within the lattice are programmable via acoustic parameters such as frequency, amplitude, and phase difference. This enables the dynamic and selective control of an individual particle, including its localization, spin, and migration, without requiring surface modification of the particle.

**Figure 25:**
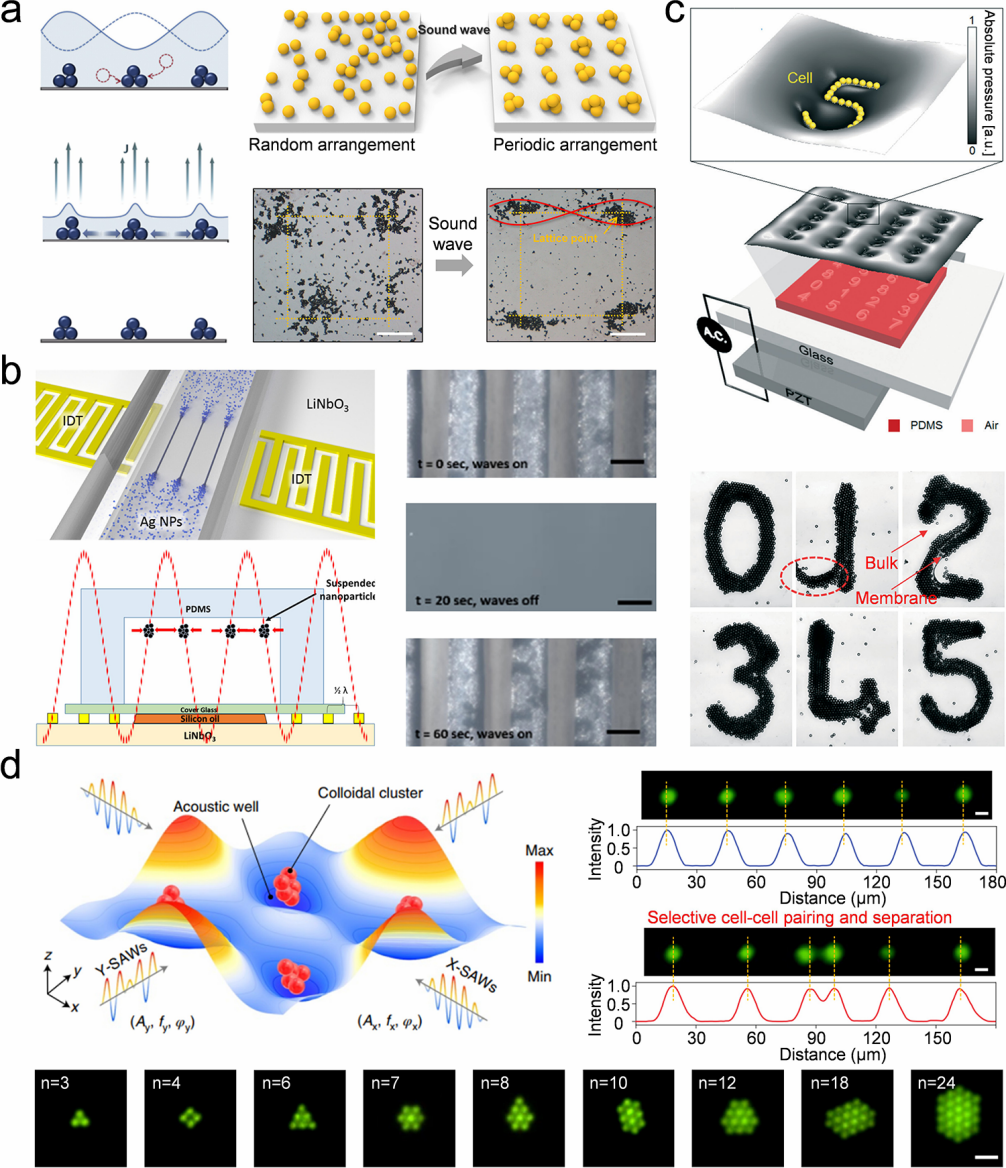
**Acoustic field-directed self-assembly of colloidal nanoparticles.** a) Schematic of the drying process of a liquid film that is resonant with a low frequency standing wave. The flow resulting from the standing wave could collect nanoparticles at the antinode, forming an array of colloids. Reproduced with permission from ref 374. Copyright 2016 American Chemical Society. b) Assembled Ag microstructures induced by acoustic waves generated by an interdigital transducer. Chloride ions are added to the Ag suspension to remove the polyacrylic capping on the particle surface, facilitating the sintering and formation of a stable Ag microstructure. Reproduced with permission from ref 375. Copyright 2018 Elsevier. c) Patternable self-assembly of colloidal nanoparticles with the assistance of acoustic waves. Due to the different mechanical strengths between the PDMS and air, nanoparticles accumulate in the nonperiodic potential well, yielding a patternable self-assembly with a line resolution of 50 μm. Reproduced with permission from ref 376. Copyright 2019 Royal Society of Chemistry. d) Reconfigurable acoustic lattices created by harmonic acoustics for dynamic and selective particle manipulation. The location, the morphology, and the size of the potential wells in the lattices depend on the frequency, amplitude, and phase difference of the harmonic acoustics. Reproduced with permission from ref 377. Copyright 2022 Springer Nature.

Due to the ability to precisely program particles, the particle manipulation based on the acoustic waves is also called acoustic tweezers.^378^ It has attracted tremendous interest since the acoustic tweezers are contact-free with samples and suitable for various targets, including particles and cells, regardless of their size and shape.^379–381^ Acoustic tweezers are usually combined with microfluidics, where the formation of periodic acoustic potential wells relies on the microfluidic channel to generate standing waves, which limits the spatial distribution and selectivity of the trapped particles.^382^ To break this limitation, a quasi-2D microfluidic open chamber is structured by PDMS and glass slides (Figure 26a).^383^ This configuration generates an acoustic field within the physically boundary-less shadow waveguide, enabling the selective trapping of particles, dynamic manipulation, and transportation. To enhance the interaction between the acoustic wave and colloidal particles, microfluidic channels may be engineered as ring resonators within the acoustic devices (Figure 26b).^384^ Ring resonator devices are closed-loop waveguides that enable waves to circulate if they have resonant frequencies. By adjusting the phase of the two input signals, ring resonance occurs in the resonator to magnify the wave intensity, enhancing the particle trapping. In this way, the dynamic assembly, migration, and mixing of particles are achieved through the acoustofluidic ring resonator with a relatively low energy input. The use of microfluidics facilitates efficient and precise manipulation of particles. However, the fabrication of the microfluidic channels is complicated. To simplify the fabrication process, a multifunctional acoustic tweezer has been designed by placing a Petri dish on top of the different tweezer devices (Figure 26c).^385^ For example, when a Petri dish is placed on an array of piezoelectric transducers, standing acoustic waves can be created in the fluid layer in the Petri dish. The device allows multiconfiguration bioparticle patterning. If a Petri dish is placed on a tilted piezoelectric transducer, the generated oblique incident traveling acoustic waves can enrich and concentrate bioparticles. A 3D acoustic field can be created when a Petri dish is placed on top of a holographic IDT, enabling the concentration and trapping of bioparticles.

**Figure 26:**
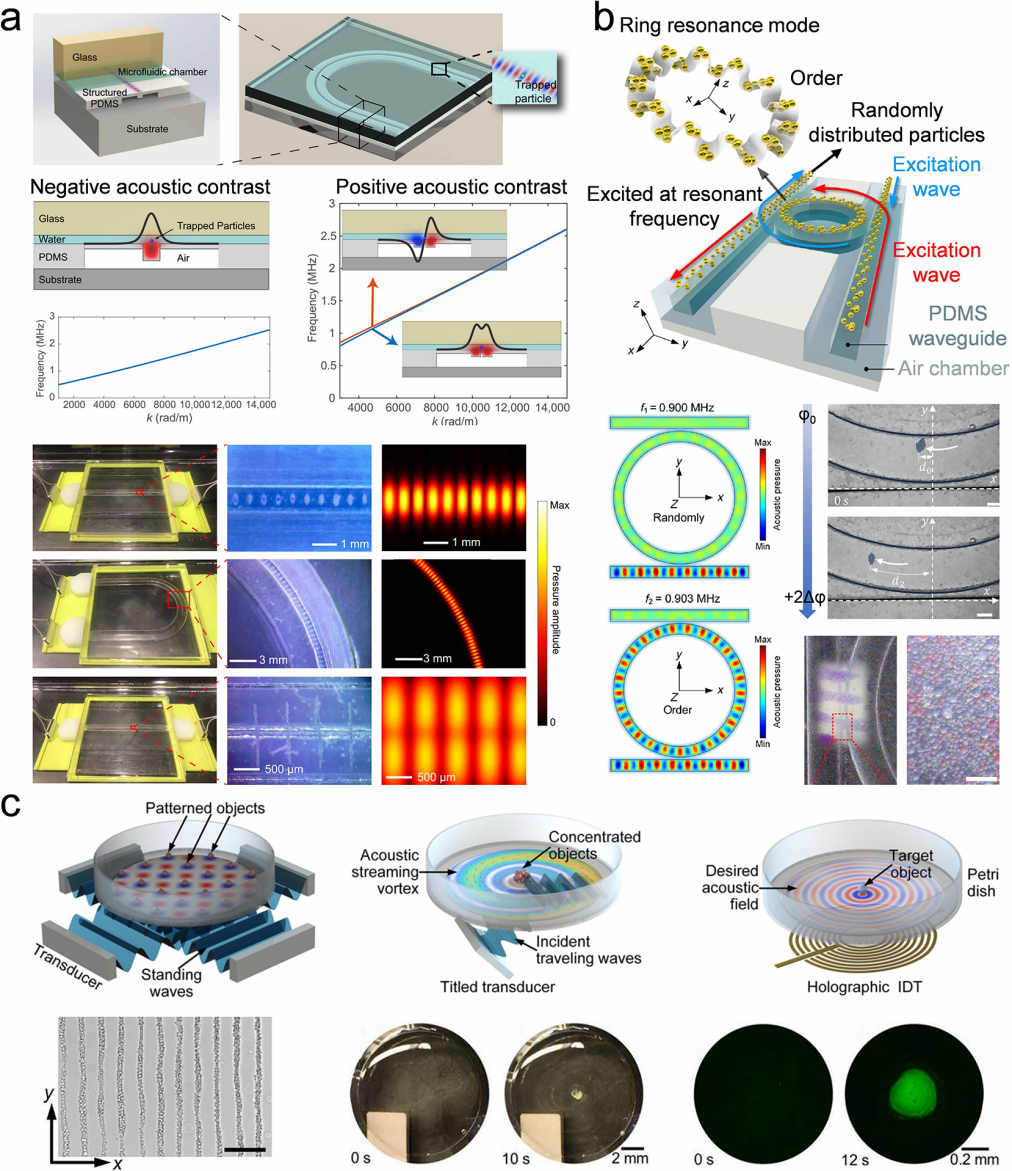
**Acoustic field-directed self-assembly of colloidal nanoparticles.** a) Acoustic tweezers with boundary-free trapping and transport channel controlled by shadow waveguides. The structure allows for the formation of an acoustic field in the physically boundaryless shadow waveguide, realizing selective trapping, manipulation, and transportation of particles. Reproduced with permission from ref 383. Copyright 2021 The American Association for the Advancement of Science. b) Acoustofluidic tweezer via ring resonance. With the assistance of the PDMS waveguide, the two input signals resonate, enabling particle patterning, migration, and mixing. Reproduced with permission from ref 384. Copyright 2024 The American Association for the Advancement of Science. c) Multifunctional acoustic tweezers by placing a Petri dish on top of the different tweezer devices, which achieve multiconfiguration bioparticle patterning, enriching micro-objects, concentration, and trapping of bioparticles. Reproduced with permission from ref 385. Copyright 2020 The American Association for the Advancement of Science.

#### 5.3.5 Summary

External field effectively programs stimuli-responsive and dynamic colloidal self-assemblies. By employing a single type of building block and leveraging an external field, it is feasible to fabricate heterogeneous structures with controllable lattice constants or predefined planar orientations with ease. Electrical fields assist in the self-assembly process of colloidal nanoparticles and can be patterned onto a chip due to the patternable nature of electrodes. This technique is most effective with highly charged particles and polar solvents. However, electrochemical reactions such as water electrolysis present significant challenges. In contrast, the magnetic field is unaffected by polar solvents^386^ or electrochemical reactions and can be applied to various functional materials. However, magnetic materials must be introduced to the colloidal nanoparticles, limiting their development as a universal strategy for assembling nonmagnetic colloidal nanoparticles. The light field can program colloidal nanoparticles selectively and precisely. However, the requirement for a high-power laser limits its application. The optical tweezers are also not suitable for particles on a large scale. Acoustic fields are suitable for contact-free, biocompatible, and precise manipulation of particles from the millimeter to the submicrometer scale. However, the fabrication of microfluidics makes it complicated to apply it as a high-throughput technique. In some cases, various fields can be coupled to induce colloidal self-assembly. For example, Fe_3_O_4_ nanoparticles modified with light-responsive capping ligand can assemble into 1D colloidal lines in a precisely controllable manner under light and magnetic fields. The assembly is reversible with a programmable aspect ratio.^251^ A detailed comparison of the four field-oriented colloidal self-assembly strategies is shown in Table 10.

**Table 10:** Summary of External Field-Directed Colloidal Self-Assembly

Self-assembly strategies	Key parameters	Outcomes	Remarks
Electrical field-directed colloidal self-assembly	Highly charged nanoparticles, colloidal concentration, polar solvent, intensity and direction of electrical field, viscosity of the solvent, hydrophilic electrodes, and the colloidal concentration.	Films, patterns, or pixels of colloidal assemblies.	High resolution (∼μm).
Magnetic field-directed colloidal self-assembly	Nanoparticles coupled with magnetic materials, colloidal concentration, intensity, and the direction of the magnetic field.	Films, patterns, or pixels of colloidal assemblies.	High resolution (∼μm).
Light field-directed colloidal self-assembly	Light intensity, polarization, phase, shapes, and the colloidal concentration.	2D and 3D aggregates of colloidal nanoparticles.	High resolution (∼μm).
Acoustic field-directed colloidal self-assembly	Frequency and amplitude of standing surface acoustic waves, different densities, and the compressibilities of nanoparticles from the surroundings.	Cluster arrays, patterned colloidal aggregates.	High resolution (∼μm), outcome size from tens to hundreds of cm^2^.

### 5.4 Nanofabrication-Assisted Colloidal Self-Assembly

Nanofabrication strategies, including transfer printing, nanoimprinting, and photolithography, offer an additional dimension for processing self-assembled colloidal assemblies. A representative approach involves intentionally creating defects at the specific locations, endowing the structure with functionalities such as laser cavities and photodiodes.^387–391^ Furthermore, nanofabrication allows for the precise programming of both 2D and 3D morphologies, which in turn tailors the functional properties of the colloidal assemblies.

#### 5.4.1 2D Colloidal Self-Assembly

The nanofabrication methods can shape colloidal self-assembled entities. For example, a colloidal crystal film prepared by dip coating can be etched into desired patterns by a bunch of lasers (Figure 27a).^392^ Besides the laser etching, physical confinement techniques are also promising strategies to program the morphology of colloidal self-assembly.^393^ For example, taking advantage of imprinting, an array of 3D nanoparticle/metal bilayer heterostructures with predesigned patterns is fabricated (Figure 27b).^130^ The bilayer heterostructures consist of phase-change VO_2_ and metallic Au colloidal nanoparticles, as well as thin films of Ni. The VO_2_ transitions to a metal from an insulator with changing temperature, changing the shape of the bilayer heterostructure. Au nanoparticles are used to lower the phase change temperature of VO_2_, and the Ni works as a conductive supporting layer. A 3D chiral optical meta-surface that is thermally reconfigurable is constructed. In addition to modifying the morphology, colloidal self-assembly can be endowed with various functions with the assistance of nanofabrication methods. For example, a period array of nanoimprinted microshallow pits onto a monolayer nanoparticle array enables the monolayer assembly to reflect the polarized light selectively (Figure 27c).^122^ This is because the concave pits cause a double reflection when light incident onto the inside surface of the concavity, leading to a polarization rotation.^394^ Thus, the monolayer nanoparticle array diffracts light and simultaneously reflects the polarized light, showing great potential in message security.

**Figure 27:**
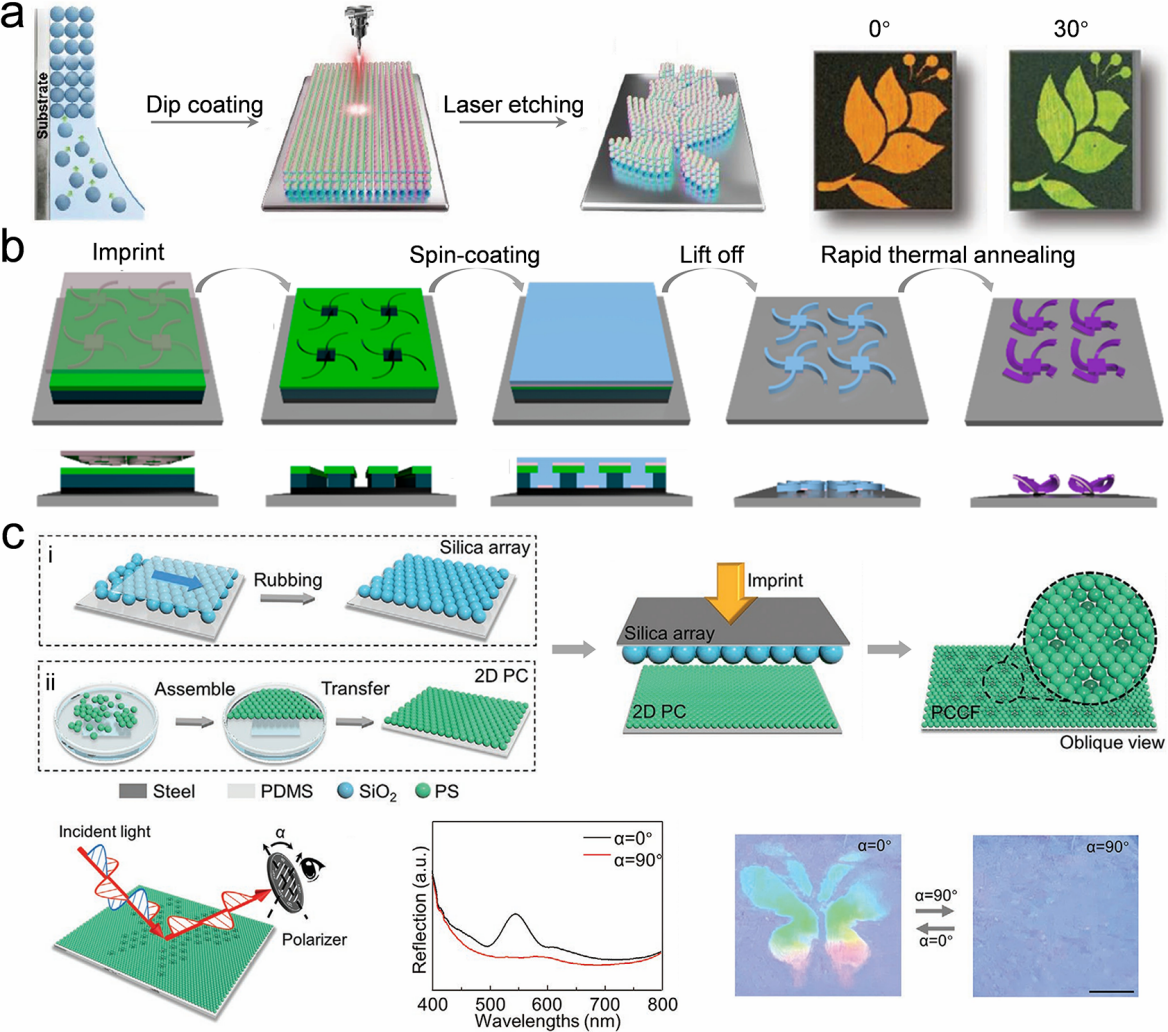
**Nanofabrication-assisted 2D colloidal self-assembly.** a) Patterned colloidal self-assembly made by the dip-coating method and laser etching sequentially. Reproduced with permission from ref 392. Copyright 2023 Wiley-VCH. b) Schematic illustration of fabricating thermally reconfigurable, 3D chiral optical meta-surface using the imprinting technique. Reproduced with permission from ref 130. Copyright 2023 American Chemical Society. c) Construction of a period array of microshallow pit structures. The period array of shallow pits is imprinted on a PS monolayer, endowing the PS monolayer with the ability to selectively reflect polarized light. Thus, the structure can diffract incident light and selectively reflect polarized light. Reproduced with permission from ref 122. Copyright 2021 Wiley-VCH.

#### 5.4.2 3D Colloidal Self-Assembly

In addition to the 2D colloidal self-assembled structures, micro/nano-fabrication techniques can also be used to modify the 3D architecture of colloidal self-assembly. An example is the polymerization of photoresist infused in the voids of PS nanospheres (Figure 28a).^19^ The uncured part of the colloidal self-assembly is then removed by organic solvent, leaving an inverse opal with 3D predesigned morphology.^19^ Besides, the functions of colloidal self-assembly can be extended by programming its 3D architecture via nanofabrication approaches to form periodically arranged subwavelength photonic nanostructures. For example, quantum dot (QD) self-assemblies exhibit distinct properties if they are packed into different architectures (Figure 28b).^395^ The QD self-assemblies with a 2D mesh architecture enhance light absorption at a specific wavelength. 1D grating QD assemblies can selectively diffract polarized light. Through high-resolution transfer printing, the 1D grating can be stacked into a multilayer woodpile architecture with a predesigned tilting angle, making it responsive to chiral light. The nanofabrication technique provides a strategy to program the shape, morphology, architecture, and hierarchy of the obtained colloidal self-assemblies in a well-controlled manner. With the help of the nanofabrication approaches, the colloidal self-assemblies with integrated functions can be well-designed and constructed.

**Figure 28:**
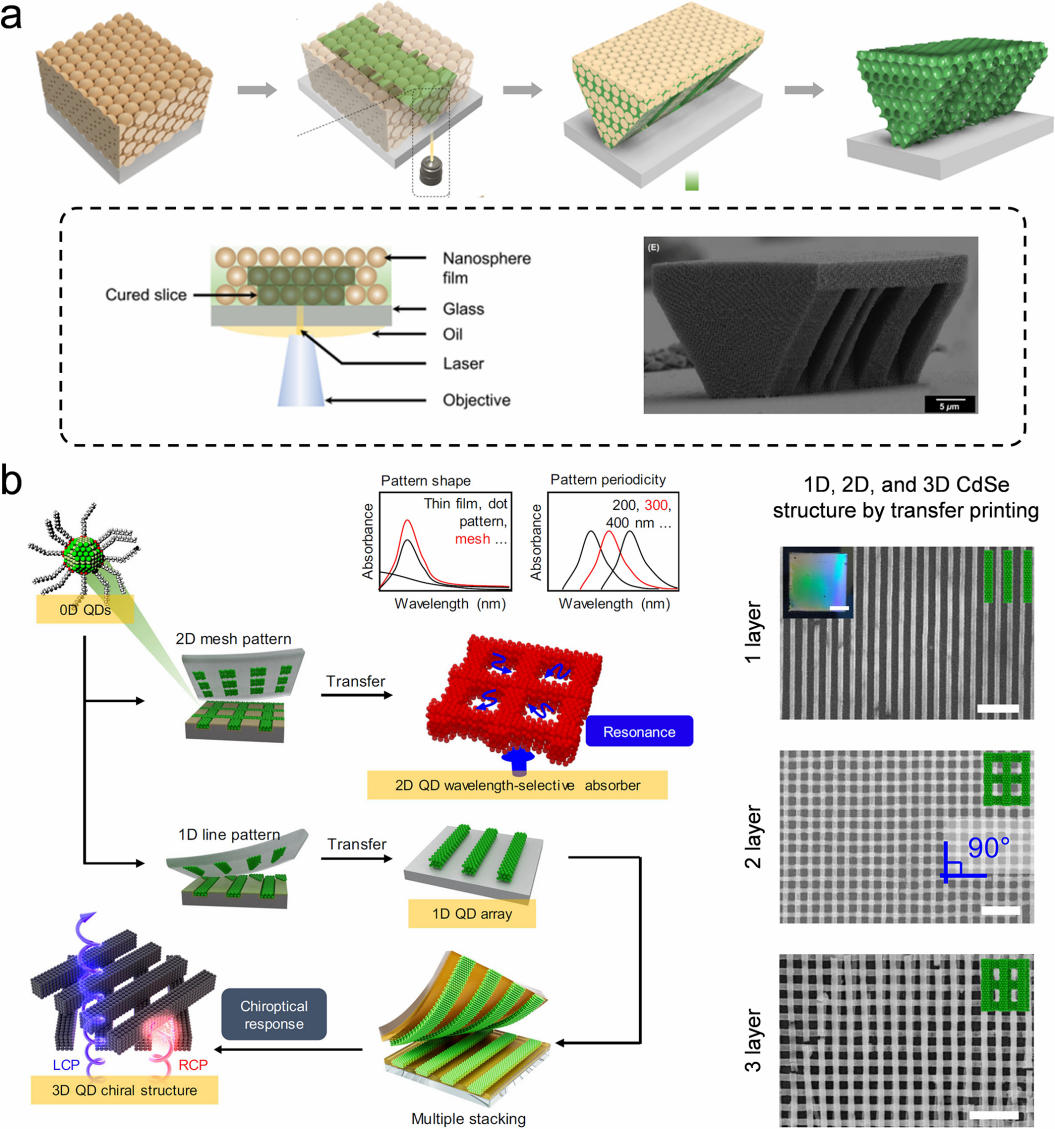
**Nanofabrication-assisted 3D colloidal self-assembly.** a) Construction of 3D structures taking advantage of laser etching. The construction is realized by selective polymerization of photoresist infused in the voids of PS nanospheres. Reproduced with permission from ref 19. Copyright 2021 American Chemical Society. b) Schematic illustration of the design for 1D, 2D, and 3D optical quantum dot (QD) nanoarchitectures, which exhibit different properties. Reproduced with permission from ref 395. Copyright 2021 American Chemical Society.

#### 5.4.3 Summary

Nanofabrication techniques effectively modify colloidal self-assembled entities, such as altering the 2D or 3D morphology of the colloidal self-assembly and enriching its functionalities. Consequently, these techniques have been extensively employed in the processing of metamaterial self-assembly to create meta-surfaces. However, nanofabrication methods are constrained by limitations related to resolution, equipment, and high costs. Table 11 provides a summary of general nanofabrication techniques.

**Table 11:** Summary of Nanofabrication-Assisted Colloidal Self-Assembly

Self-assembly strategies	Key parameters	Outcomes
Laser-etching	Intensity and wavelength of the laser, the size and shape of the laser spot, and the optical, mechanical, and thermal properties of the colloidal self-assembly.	Patterns and 3D architected colloidal self-assembly.
Nanoimprinting	Imprinting temperature, pressure, time, and imprinting template.	Patterns or arrays of colloidal self-assembly, metasurfaces.
Photolithography	The intensity and wavelength of the laser, the choice of photocuring materials, the resolution of the laser, and the development temperature and time.	Patterns and 3D architected colloidal self-assembly.
Transfer printing	Surface modification of the stamp, transfer printing pressure, temperature, and time.	3D architected colloidal self-assembly.

### 5.5 Self-Assembly of Anisotropic Colloidal Particles

During the self-assembly, the resulting structure is maintained by van der Waals forces or interactions between the capping ligands. Due to the isotropic properties, colloidal nanoparticles tend to stack unidirectionally, resulting in the formation of closely packed lattices. Introducing directional interactions through surface patches, anisotropic particle shapes, or biomolecular patterning enables programmable assembly pathways and access to complex architectures, including colloidal clusters, 1D colloidal chains, and superlattices.^252,396^ In this section, we will introduce strategies for assembling anisotropic colloidal particles, including patchy colloidal particles, inorganic colloidal particles with anisotropic shapes, biocolloids, and DNA origami.

#### 5.5.1 Patchy Colloidal Particles

The nanoparticle patches include soft and hard patches. An example of synthesizing soft patchy nanoparticles is to disperse a triblock terpolymer into selective solvents.^397^ Dispersion of an ABC triblock terpolymer in a nonsolvent for B generates particles with B-core and A/C corona patches. After being transferred into a nonsolvent for A and B, the B-core particles develop into monovalent AB^C^ or AB^C^A, depending on the volume ratio of the core-forming segments (Figure 29a). Because of the different wettability of A and C, various assemblies can be fabricated due to the anisotropic interaction between the corona patches, including spherical clusters, colloidal polymers, and mixed superstructures. Besides, variation in the ratio of block lengths N_C_/N_A_ can direct ABC triblock terpolymers to assemble into multicompartment nanostructures, including spheres, cylinders, bilayer sheets, and vesicles.^398^

**Figure 29:**
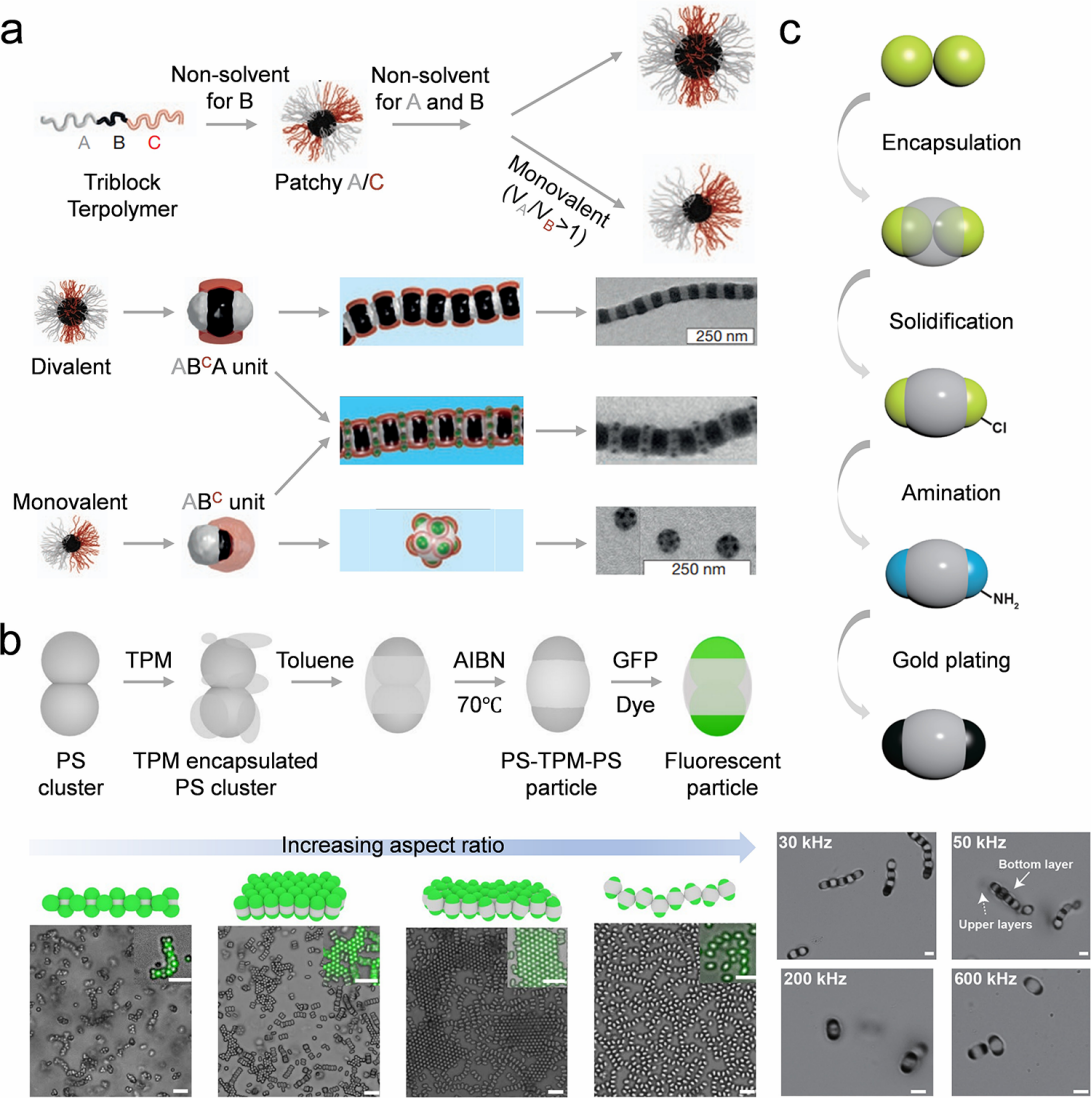
**Self-assembly of patchy particles.** a) Preparation and configuration of soft colloidal building blocks with different patches on them and resultant coassembly of hierarchical structures. Reproduced with permission from ref 397. Copyright 2013 Springer Nature. b) Self-assembly of superlattices by PS-TPM-PS particles. The aspect ratio of the PS-TPM-PS particles determines the final colloidal superstructure. Reproduced with permission from ref 92. Copyright 2020 Springer Nature. c) Self-assembly of metal-patchy particles. The resultant colloid superstructure is induced by dipole polarization under AC electric fields and can be regulated by the frequency of the AC electric field. Reproduced with permission from ref 399. Copyright 2021 American Chemical Society.

The preparation of hard patchy nanoparticles is like synthesizing colloids with multicavities, but without the etching process. The colloid cluster is encapsulated by TPM first and forms a PS-TPM-PS triblock particle after being treated with toluene and polymerization (Figure 29b).^92^ The aspect ratio of the obtained triblock particles is related to the amount of TPM. With a controllable aspect ratio, these triblock building blocks assemble into various superstructures and superlattices featuring optimized pole-to-pole or center-to-center interactions without further surface treatment. In addition to the chemical patches, reconfigurable superlattices can be fabricated by electrically inducing dipole polarization of patches (Figure 29c).^399^ By coating triblock nanoparticles with Au on the patches, particles could orient under an alternating current (AC) electric field to align their long axis with the substrate. With different AC frequencies, various superlattices can be obtained.

The patchy particles cannot selectively connect building blocks since the anisotropic properties result from the patchy modification of chemical and physical properties. DNA can modify the building blocks to selectively bond the patchy building blocks with the target building blocks.^111,400,401^ The selectivity derives from the DNA base-pair interaction between the four nucleobases (A-T and C-G). DNA patchy particles can be obtained in two steps: First, a colloidal cluster with different numbers of nanoparticles is encapsulated by TPM; second, the colloidal cluster is functionalized with DNA to generate the DNA patches (Figure 30a).^402^ In this way, colloidal assemblies that mimic the geometry and the chemistry of molecules can be built.^127^ The patchy clusters can also be synthesized by mixing the nanoparticles and TPM microdroplets.^90^ With a proper size ratio, nanoparticles could aggregate onto the TPM liquid droplet to form tetrahedral clusters. Moreover, the DNA can be coated on the center of the four triangular faces. The DNA on the patches is designed with self-complementary sticky ends so that patches on different particles are attractive, thus forming a colloidal diamond (Figure 30b).

**Figure 30:**
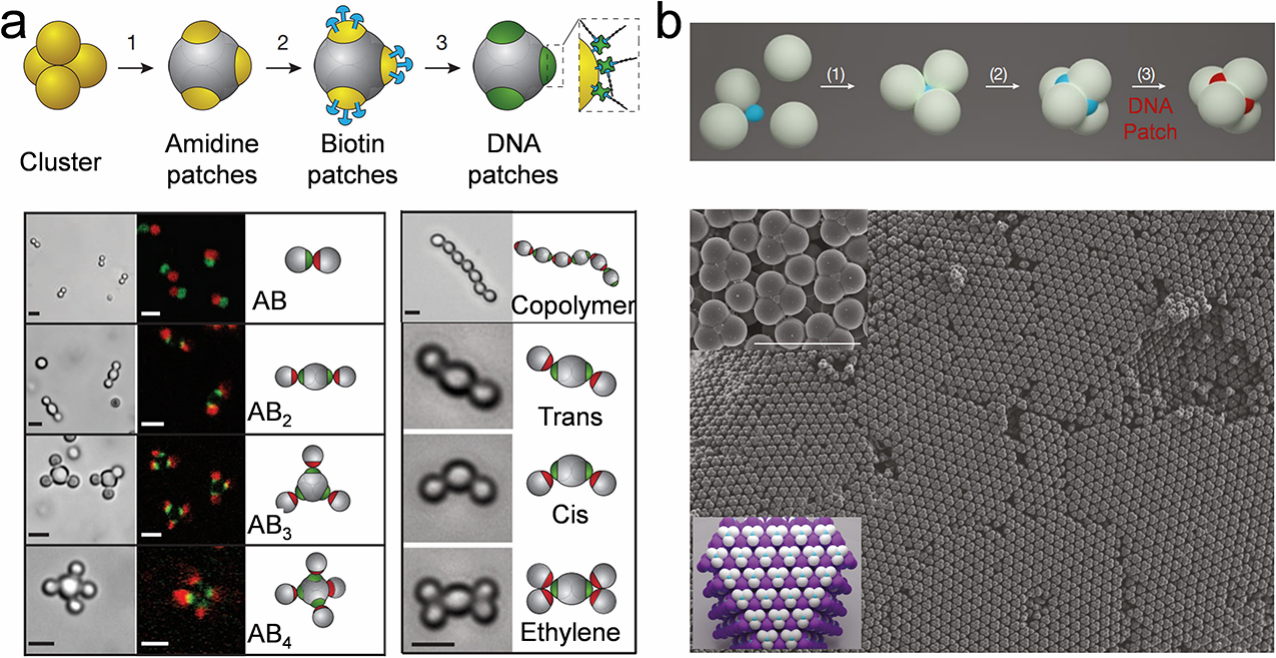
**Self-assembly of DNA-patchy particles.** a) Preparation of DNA patchy particles and obtained various nanostructures that mimic not only the geometry but also the chemistry of molecules. Reproduced with permission from ref 402. Copyright 2012 Springer Nature. b) Assembly of a photonic diamond by a tetra cluster with a DNA-patchy center. The interaction between DNA-patchy centers facilitates the formation of diamond crystals. Reproduced with permission from ref 90. Copyright 2020 Springer Nature.

#### 5.5.2 Polyhedral Inorganic Colloidal Particles

Colloidal building blocks with anisotropic morphology, such as polyhedral inorganic nanoparticles, promise the design and fabrication of superstructures.^107,403–406^ On the one hand, the anisotropic shape of colloidal particles favors directional bonding. On the other hand, the molecular crystallinity and anisotropic properties of some colloidal particles, which are initially restricted within individual crystallites, may be mutually synchronized through assembly and particle alignment. Due to the enormous potential, the self-assembly of particles with anisotropic morphologies has been explored.^407^ The self-assembly of shape-anisotropic nanoparticles can be realized by applying external stimuli to the responsive polyhedral.^85,408,409^ For instance, Fe_3_O_4_ nanocrystals with different shapes can be aligned orderly in the presence of a magnetizing field when a hexane droplet containing Fe_3_O_4_ nanocrystals is dispensed at the diethylene glycol-air interface.^410^

Except for external force, the self-assembly of polyhedral colloids can also be driven by other forces. For instance, polyhedral colloids with patches have been assembled into 1D chains mediated by capillary force through a liquid bridge (Figure 31a).^411^ Facilitated by unidentical crystallography, patches can be prebuilt on specific facets of the polyhedral particles by chemical modification. Then, a TPM liquid film is deposited onto the patchy facets, which can induce the bonding of polyhedral particles under slight agitation, triggering the merging of the TPM film from the patches of two particles (Figure 31a-i). During this process, the liquid bridge between two particles provides a solid and reconfigurable capillary force, maximizing the contacts between the patches. Then, polyhedral particles with various morphologies can be assembled into 1D chains (Figure 31a-ii). Introducing a responsive material to the TPM film on the patchy facets allows the obtained 1D chains to be functionalized with stimulus responsiveness.^407^ Besides the capillary forces,^412^ self-assembly of polyhedral nanoparticles can also be achieved using DNA hybridization^112^ and polymer matrix.^403^ However, extensive modification is required due to the low stability of such nanoparticles. Small-molecule ionic amphiphiles are added to the colloidal suspension to address this issue (Figure 31b).^413^ The amphiphiles can be adsorbed on the polyhedral particles to stabilize the suspension. Furthermore, the small molecules form micellar nanoparticles and act as depletants, generating a depletion force on particles and driving the polyhedral nanoparticles to bond face-to-face. The depletion force is regulatable by the roughness of the substrate, thus programming the orientation of the polyhedral particles and producing superframeworks with hierarchically coordinated crystallinity and micropores.

**Figure 31:**
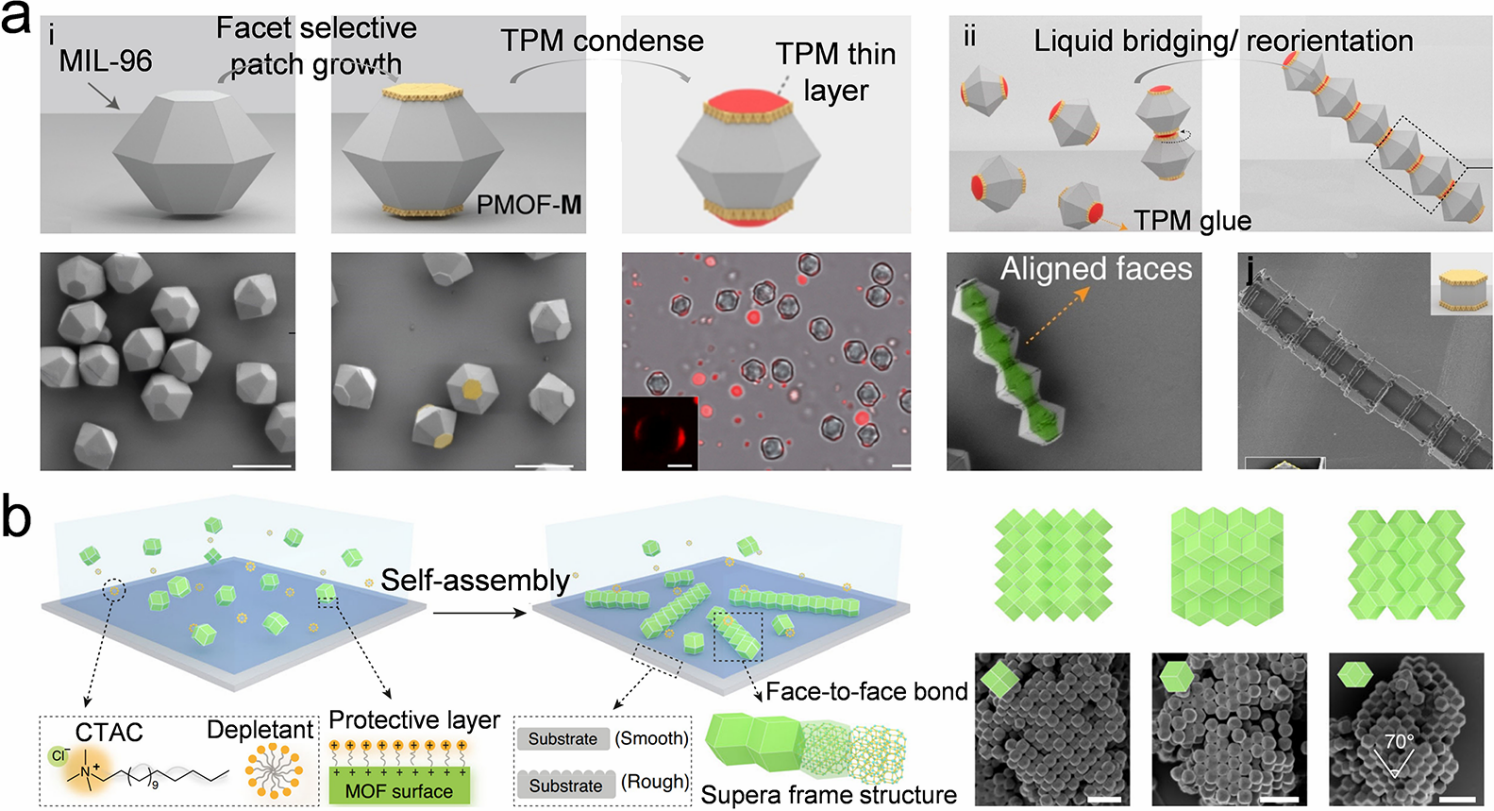
**Self-assembly of shape-anisotropic particles.** a) Self-assembly of polyhedral particles actuated by patches on specific facets. TPM liquid film on the patchy facet induces the bonding of polyhedral particles under slight agitation. Reproduced with permission from ref 411. Copyright 2022 Wiley-VCH. b) Low-dimensional assemblies of polyhedral particles. Small-molecule ionic amphiphiles are added to the colloidal suspension to stabilize the polyhedral particle suspension and to exert a depletion force for assembly. Reproduced with permission from ref 413. Copyright 2022 Springer Nature under CC BY 4.0 (https://creativecommons.org/licenses/by/4.0/).

Apart from the structures mentioned above, natural structures also involve chiral structures, which can selectively reflect polarized light.^414^ Similar structures show promising potential in light manipulation,^415^ message security,^416^ and immunological.^417^ Generally, artificial chiral structures are constructed with chiral building blocks,^418^ template orientation,^233,419,420^ or surface modification of colloidal building blocks.^421,422^ However, preparing chiral structures with achiral building blocks remains challenging, as spontaneous thermodynamics-driven colloidal self-assembly is associated with prohibitively high activation barriers. A strategy to solve this problem is to compress nonclosely packed achiral corner-sharing structures into new states with higher packing fractions by reconfiguring building blocks with tetrahedral geometry. This idea is experimentally demonstrated using tetrahedral gold nanoparticles (Figure 32a). Tetrahedral gold nanoparticles pack into a bilayer interlacing structure (honeycomb structure) with a large interparticle distance. In-plane compression of this nonclosely packed structure through reducing the repulsive force or increasing the van der Waals force between tetrahedral nanoparticles leads to a transfer into a chiral pinwheel structure.^423^ The chiral degree depends on the particle size, ionic strength, and molecular additive in the colloidal suspension. Another strategy for fabricating chiral structures from achiral building blocks is to increase the asymmetry of the building blocks. By modifying the TPM to the polyhedral nanoparticles, the symmetry of the polyhedral building blocks can be designed.^424^ Due to the modified symmetries, the patchy polyhedral particles can assemble into chiral clusters (Figure 32b). Generally, chirality is treated as a binary left or right characteristic in chemical disciplines. However, it is a geometrical property described by continuous mathematical functions. Current strategies can hardly fabricate chiral colloidal self-assemblies with continuous chiral geometries. Taking advantage of the electrostatically restricted assembly process, nanostructured microparticles with bowtie shapes are synthesized by mixing an aqueous solution of Cd^2+^ and L- or d-cystine, which show continuously variable chiral geometries (Figure 32c).^418^ The bowties are assembled from nanoribbons containing helical chains of cystine interconnected by Cd^2+^ ions. The chirality depends on the ratio of Cd^2+^ and L- or d-cystine used in the assembling process. The pitch, width, thickness, and length of the bowties are programmable by regulating the pH, solvent, and ionic strength in the synthesis system.

**Figure 32:**
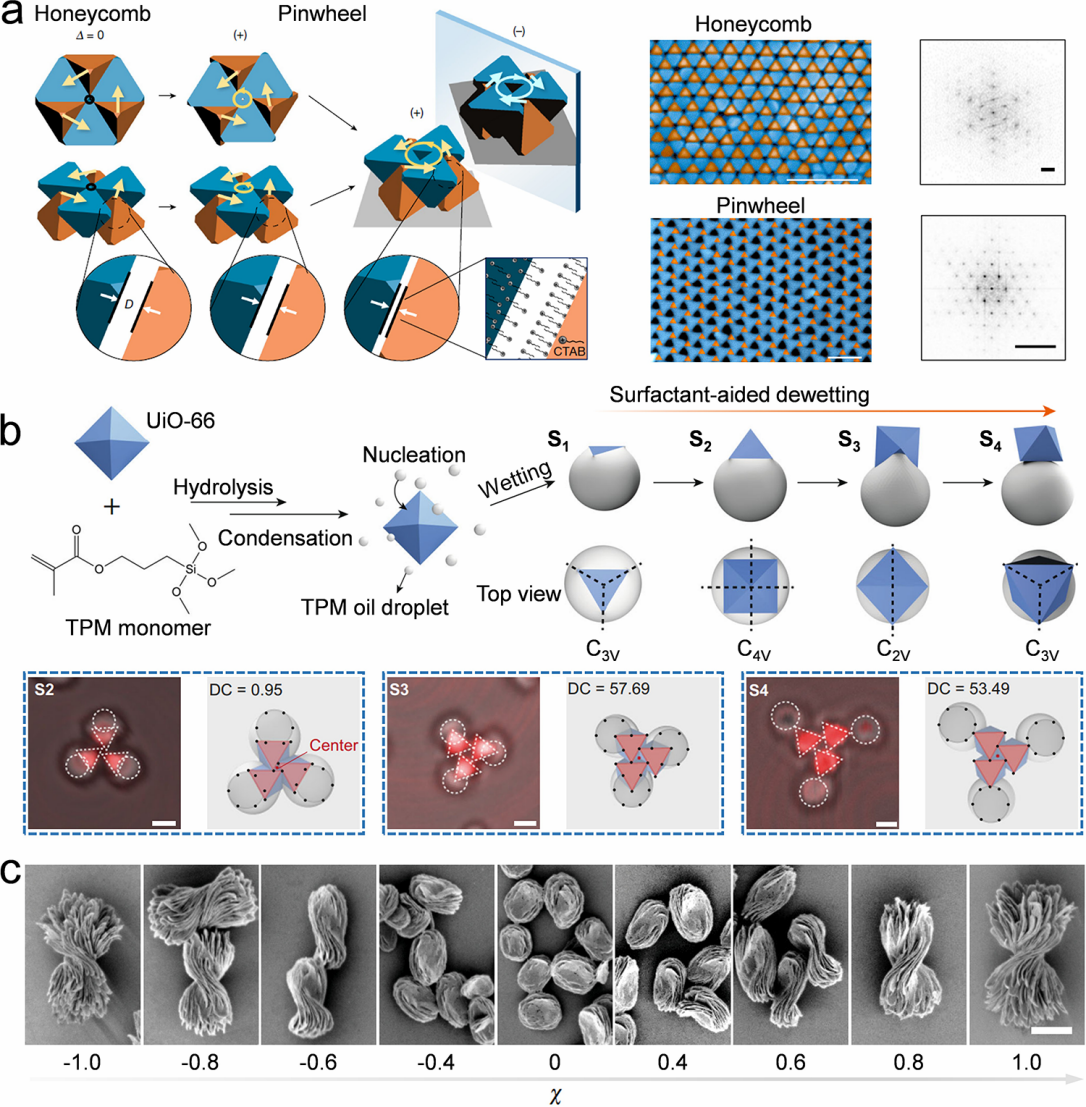
**Colloidal self-assembly of chiral structure by shape-anisotropic particles.** a) Corner-sharing and pinwheel bilayer superlattices with corner-to-edge connections from gold tetrahedral nanoparticles. In-plane compression of the nonclosely honeycomb structure leads to the transfer of the structure into a chiral pinwheel structure. Reproduced with permission from ref 423. Copyright 2022 Springer Nature. b) Synthesis of Janus particles with various patch shapes/symmetries. The Janus particles with different patch symmetries can assemble into chiral clusters. Reproduced with permission from ref 424. Copyright 2023 Springer Nature under CC BY 4.0 (https://creativecommons.org/licenses/by/4.0/). c) Nanostructured microparticles with bowtie shapes showing continuously variable chiral geometries. The chirality depends on the ratio of Cd^2+^ and L- or d-cystine used in the assembling process. Reproduced with permission from ref 418. Copyright 2023 Springer Nature.

#### 5.5.3 DNA Origami

DNA has been widely used to direct the self-assembly of superstructures as building blocks,^425^ frameworks,^110,426^ or patches^70,400,427^ due to its specific selectivity between base pairs.^71^ Colloids made by DNA origami are one important type of shape-anisotropic colloids. DNA origami involves folding a long scaffold strand into a designed 2D or 3D structure using many staple strands. The selective bonding between predesigned A, T, C, and G enables a long scaffold strand to fold into the designed structure by forming double-stranded helices. The obtained voxels can bond with each other to create 3D frames, in which the lattice symmetry is determined by the DNA voxels (Figure 33a).^110^ The obtained 3D frames can integrate nano-objects within the scaffold, such as gold nanoparticles, quantum dots, and proteins, thus enabling the object’s valence and coordination to be determined by the frame’s vertices. One significant advantage of DNA origami colloids is that they are a potential alternative to fabricating special superstructures due to the programmable structure of DNA origami voxels. For example, DNA origami tetrapods are designed, whose four arms serve as a connecting patch to their neighbors (Figure 33b).^428^ The pattern of binding extensions on the end surface of each arm provides the torsional binding potential, favoring a 60° rotation between tetrapods. The obtained rod-connected diamond cubic lattice is predicted to show a wide and robust PBG.

**Figure 33:**
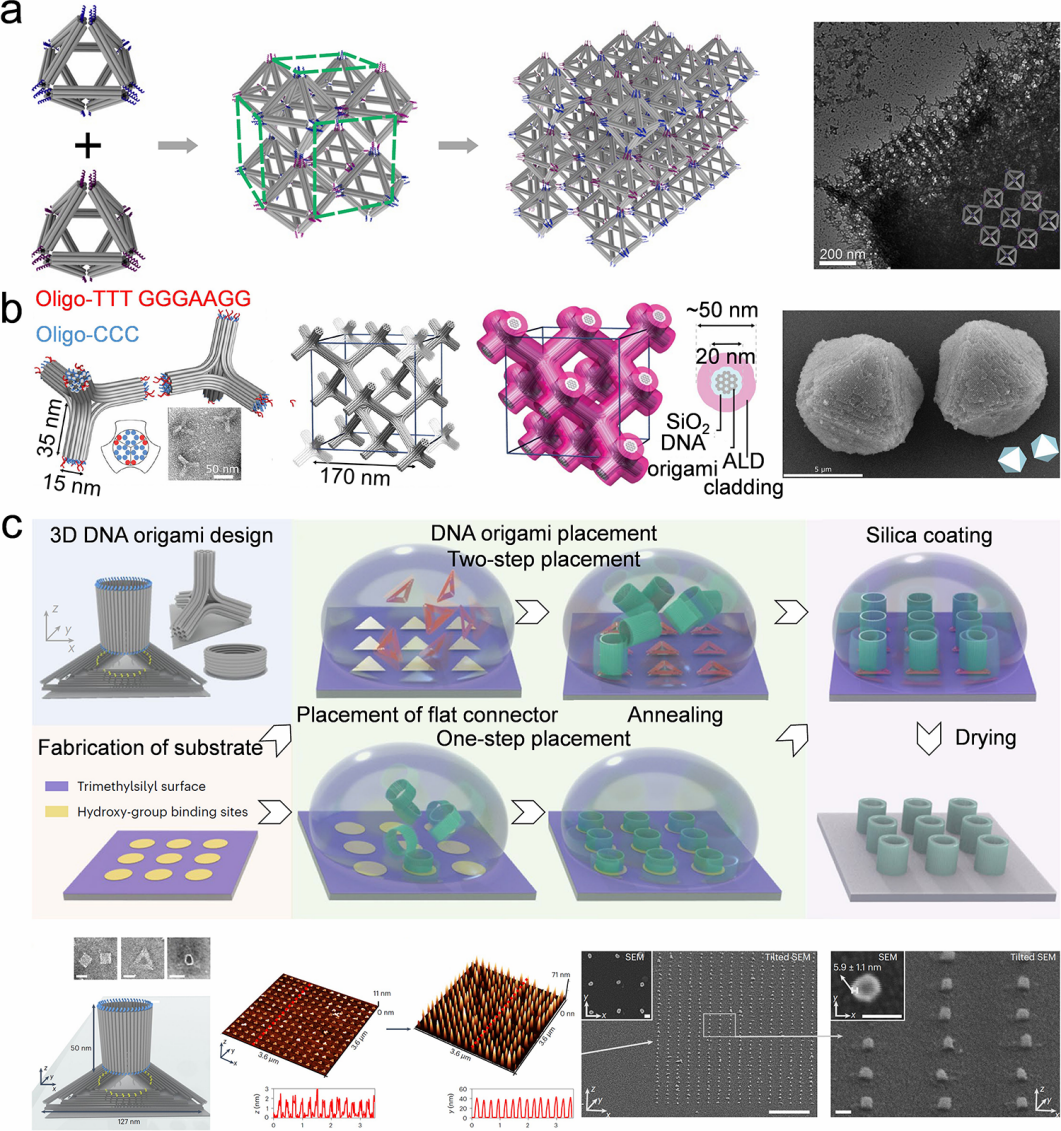
**Self-assembly of DNA origami.** a) Assembly of octahedra frames into DNA lattice, followed by a TEM image. Reproduced with permission from ref.^110^ Copyright 2020 Springer Nature. b) Rod-connected diamond cubic lattice constructed by DNA origami tetrapods. Reproduced with permission from ref 428. Copyright 2024 The American Association for the Advancement of Science. c) Assembly of 3D hybrid DNA-silica structures based on DNA origami replacement method. Reproduced with permission from ref 431. Copyright 2023 Springer Nature.

In addition to special structure, DNA origami is often combined with lithographic nanopatterning (DNA origami replacement) to create large-scale 2D or 3D arrays of precisely placed DNA structures.^429^ For the 2D arrays, planar DNA origami building blocks are directly deposited on a substrate with modified wettability patterns.^430^ The difference in surface potential facilitates the regioselective assembly of DNA origami at the desired location. To obtain 3D DNA assemblies, 3D origami voxels are used. However, the 3D DNA voxels sometimes cannot orient uniformly on the substrate. For example, DNA hollow nanotubes with a high aspect ratio exhibit a large contact area with the hydrophilic spot, leading to an undesired flat-lying orientation. In this condition, a connector with predesigned strands can be deposited in the hydrophilic spots first. Then, the nanotube with complementary strands can be combined with these connectors in a subsequent step. In this way, the orientation of the 3D DNA origami voxels can be controlled (Figure 33c).^431^ The obtained 3D structure can be hybridized with gold nanoparticles and silica with controllable dimensions and feature sizes.

#### 5.5.4 Summary

Using anisotropic particles as building blocks enables the fabrication of directionally packed colloidal structures due to the anisotropic properties of particles. Various particles with patches have been designed.^432,433^ Based on the simulation results, these particles can be used to construct chiral photonic crystals, crystals with diamond symmetry, and hierarchical structures.^434,435^ Despite many works based on the simulation, the demonstration of such assembly in experiments remains challenging. Particles with anisotropic shapes also show potential in constructing directional structures. However, how to stabilize the anisotropic-shaped particles in a solution needs to be addressed. DNA origami colloids would be a promising alternative for anisotropic self-assembly due to their high selectivity and programmable architectures. However, they are limited by the scalable self-assembly and size of the DNA voxels. Table 12 summarizes the colloidal self-assembly strategies for anisotropic colloidal building blocks.

**Table 12:** Summary of Self-Assembly of Anisotropic Colloidal Building Blocks

Self-assembly strategies	Key parameters	Outcomes
Soft patchy particles	Solvent, the ratio of block lengths of triblock polymers.	Spherical clusters, cylinders, bilayer sheets, and vesicles.
Hard patchy particles	Aspect ratio and morphology of the particles, particle concentration, and external trigger, such as the frequency of the electric field.	2D superlattices, chains, and colloidal clusters.
DNA-patchy particles	DNA strands, temperature, particle concentration, surface properties, morphology, and size of the particles.	Colloidal clusters, photonic diamond.
Shape-anisotropic inorganic particles	Temperature, colloidal stability, and concentration, external trigger, particle morphologies, and surface properties.	Colloidal cluster, 1D, 2D, and 3D colloidal lattices, chiral superstructures.
DNA origami self-assembly	Morphology and size of the origami voxels, design of the scaffold, and staples.	3D frames, assemblies with diamond cubic lattice.
DNA origami replacement	Design of the patterned substrates and connectors, morphology, and size of the DNA origami voxels.	2D assembly array, 3D assemblies.

### 5.6 Nature-Mimetic Self-Assembly

In nature, many organisms evolve self-assembled complex hierarchical structures that combine biological, mechanical and other functions. For example, parallel alignment of cellulosic materials endows bamboo with high strength and toughness.^436^ Self-assembled nanostructures in chameleons allow camouflage ability; the building blocks (guanine nanocrystals) are self-grown through phase separation rather than artificial synthesis.^5^ Assembly of biomolecules, such as the lipids and proteins, allows for various specified life activities in sophisticated intracorporeal environments without being disturbed. Mimicry of these self-assembly provides an opportunity for human to address challenges in fields involving materials, energy, and life science. In this section, we will use self-assembly of cellulose-based materials, self-growing self-assembly, and self-assembly of biomolecules as examples to introduce life-mimetic self-assembly.

#### 5.6.1 Self-Assembly of Cellulose-Based Materials

Cellulose-based materials attract tremendous interest as they are biocompatible, biodegradable, and can be modified to introduce new properties or to adjust their interaction with their surroundings. In addition, they can be produced from renewable resources in nature. General cellulose materials involve cellulose nanocrystals (CNC),^437,438^ bacterial nanocellulose,^439,440^ cellulose nanofibers,^441–443^ lignin nanofibers,^444,445^ and chitin nanofibers.^446,447^ Self-assembled cellulose-based materials show excellent mechanical properties and structure-dependent functions. Here, we choose CNCs as an example to show some self-assembly strategies for cellulose-based materials.

CNCs are renewable, plant-based, rod-like nanoscale colloidal particles that are extracted from nanofibrils in the plant cell wall.^448^ Their formation process depends on the deconstruction of biomass to obtain biopolymers and the reassembly of biopolymers through molecular interaction, such as the van der Waals force, the hydrogen bond, the electrostatic force, and hydrophobicity.^449–453^ CNCs show anisotropic mechanical properties and have been used to reinforce polymer composites.^438,454,455^ Besides the anisotropic morphology and the mechanical properties, CNCs also have birefringent optical properties that can be transmitted to the materials in which they are arranged, making them an outstanding material for photonic applications.^456–458^ Through evaporation of a CNC suspension, CNCs can assemble into a periodic helical arrangement that can be retained after removal of solvent (Figure 34a).^459^ The periodic helical arrangement endows the structure with unique optical properties, such as the ability to selectively reflect polarized light.^89,460^ In addition, the assembled CNCs also show stimulus-responsive ability, allowing them to be used as sensors,^459^ photonic pigments,^461^ and message security.^462^ However, the evaporation-induced CNC self-assembly is easily affected by the humidity and temperature, and it is hard to control the orientation of the CNCs. To address this issue, confined evaporation-induced self-assembly has been developed to guide the arrangement of CNCs, which assemble CNCs in a confined space, such as evaporating a CNC suspension between two hydrophilic glasses (Figure 34b).^463^ Due to the evaporation of the confined water, the CNCs are concentrated near the TCL. When the CNC concentration reaches a critical value, the internal stress in the principal plane overcomes the cohesion of the gelled suspension, generating fractures. In this process, CNCs are oriented parallel to the suspension TCL. Because of the high alignment, the obtained multiple lamellae show an anisotropic adhesion between solid surfaces.^464^ The mechanical properties of the confined evaporation-induced CNC assembly are closely related to the drying stresses, thickness, and drying temperature during the suspension drying process.^463,465^ The substrate properties are also very important to the evaporation-induced CNC assembly. To break the limitation of the substrate, a substrate-free process is developed by confining a CNC suspension within emulsified microdroplets (Figure 34c).^466^ Upon drying, the microdroplets become kinetically arrested and buckle. CNC beads are prepared with a complex surface morphology and visible colors when the water dries. The color blue-shifts with evaporating water. Besides the strategies mentioned above, many strategies have been developed to self-assemble CNCs, including microtemplating,^467^ permeable interfacial assembly,^87^ droplet evaporation,^468^ and sonication.^460^ However, these strategies can realize the CNC self-assembly at a small scale. To prepare scalable CNC photonic films, a roll-to-roll process is developed that involves surface activation, coating, controlled drying, peeling, heat treatment, grinding, and size sorting (Figure 34d).^469^ The challenges of this strategy include the scalable and uniform coating of CNC suspension. To achieve this, the polyethylene terephthalate (PET) substrate is activated by the corona discharge to make it hydrophilic, allowing the CNC suspension to spread well on the substrate. Besides, the HPC is added to the suspension to suppress the edge inhomogeneities. Then, the thickness, blading speed, and drying temperature are also optimized in this process, enabling the fabrication of scalable CNC photonic films. Various strategies for CNC and other biobased colloid self-assembly have been proposed.

**Figure 34:**
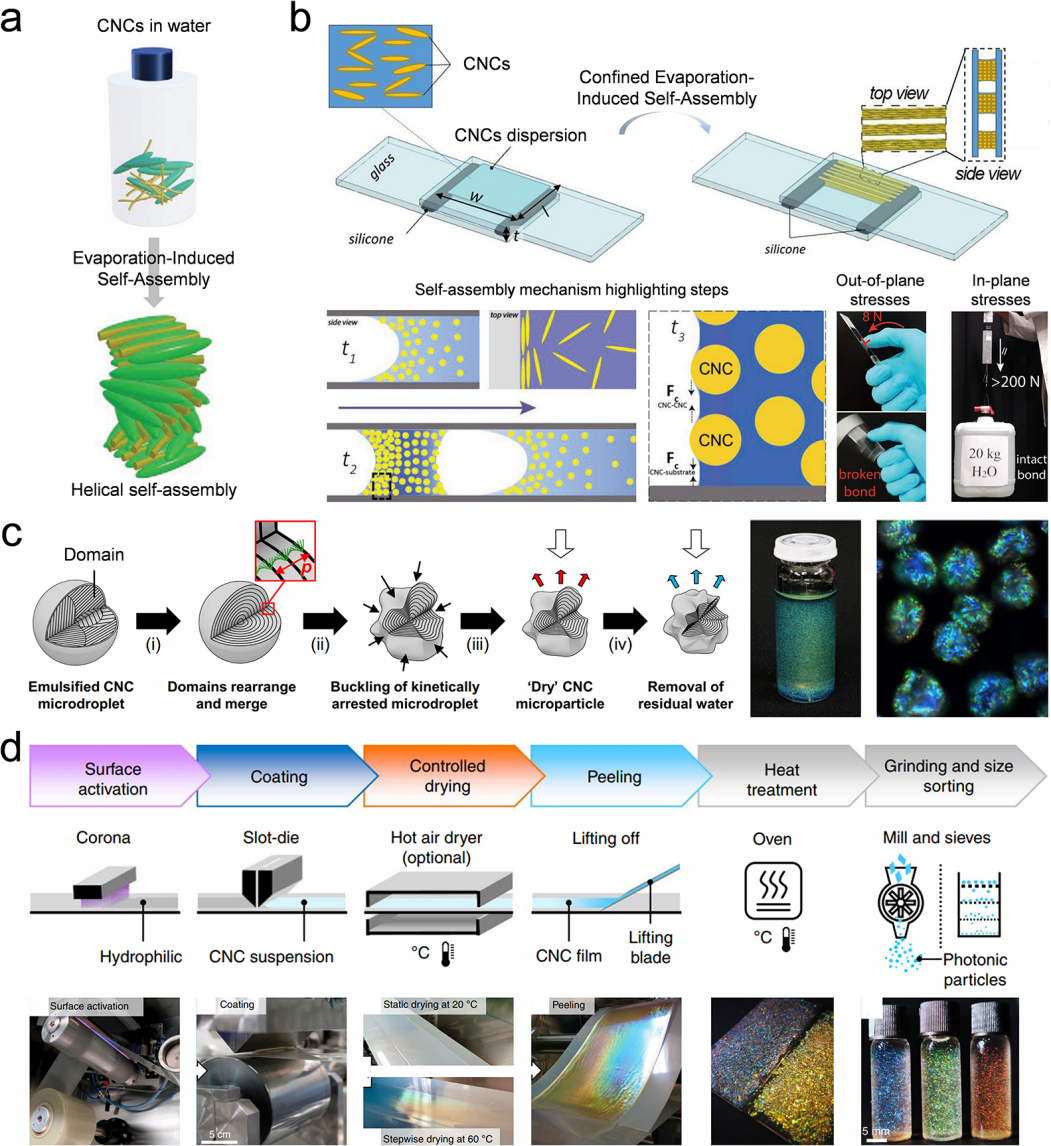
**Self-assembly of cellulose nanocrystals.** a) Schematic illustration of the self-assembly of cellulose nanocrystals (CNCs). CNCs arrange into a helical structure by an evaporation-induced self-assembly process. Reproduced with permission from ref 459. Copyright 2023 Wiley-VCH. b) Self-assembly of CNC lamellae by the confined evaporation-induced self-assembly. During the drying process, the TCL of the suspension moves in a stick–slip mode to generate multiple homogeneous lamellae. Reproduced with permission from ref 463. Copyright 2023 Springer Publishing Center. Reproduced with permission from ref 464. Copyright 2020 The Authors, Published by Wiley-VCH. c) Preparation of photonic pigments via the confined self-assembly of cellulose nanocrystals. An aqueous CNC suspension is emulsified in hexadecane by a microfluidic device. With the evaporation of the water, the microdroplets become kinetically arrested and buckle, forming CNC beads with a complex surface morphology and visible colors. Reproduced with permission from ref 466. Copyright 2022 Nature Publishing Center. d) Schematic of the roll-to-roll process to assemble a CNC suspension into scalable photonic films and microparticles. Images indicate the implementation and the products of the key steps shown in the flowchart. Reproduced with permission from ref 469. Copyright 2022 Nature Publishing Center.

#### 5.6.2 Self-Grown Colloidal Self-Assembly

In nature, many organisms evolve nanostructures through mechanisms distinct from artificial self-assembly. Rather than adding prefabricated building blocks, these structures form through phase separation: Macromolecules directly precipitate from a surrounding matrix,^470,471^ and the resulting nanostructures are stabilized and arrested by specific biomolecular interactions. Inspired by the principle, microphase separation has been developed to mimic self-grown assembly.^472–476^ Examples include a linear-bottlebrush-linear triblock copolymer that has been used to fabricate nanostructures (Figure 35a).^477^ During the drying process of the triblock copolymer solution, microphase separation occurs, and linear chains aggregate into rigid domains. At the same time, the bottlebrush strands form a supersoft matrix, leaving a self-grown nanostructure. The degree of polymerization of linear chains dominates the size of the rigid domain, and the bottlebrush strands can control the distance between rigid domains. The resultant film is soft on touch, stiff upon deformation, and colored for appeal or camouflage. Microphase separation can also be produced by polymerizing two mixed monomers, such as un-cross-linked PS and methyl methacrylate monomer (MMA).^478^ After triggering the polymerization, the MMA monomer is depleted from the system when the PS matrix resolidifies, arresting the coarsening process (Figure 35b). The content of MMA determines the size of the obtained spherical poly­(methyl methacrylate). However, the microphase separation only occurs near the surface of the products due to the higher mole fraction of MMA than that in the bulk.

**Figure 35:**
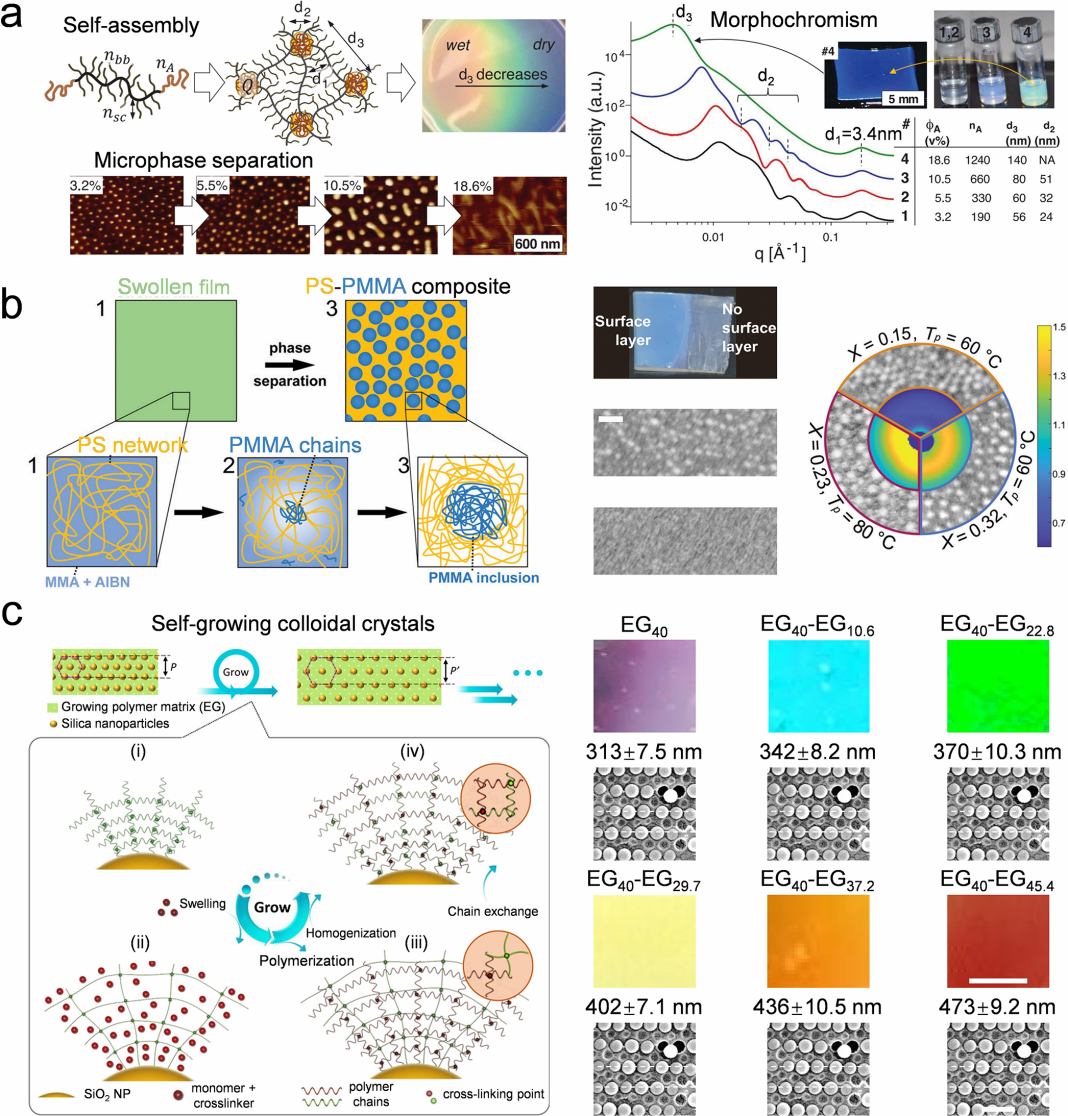
**Self-grown colloidal self-assembly.** a) Self-assembled nanocomposites by self-grown building blocks through microphase separation triggered by solvent evaporation. Linear chains aggregate into rigid domains, and the bottlebrush strands form a supersoft matrix. Reproduced with permission from ref 477. Copyright 2018 The American Association for the Advancement of Science. b) Self-assembly of amorphous colloidal crystals through microphase separation of two immiscible polymers triggered by polymerization. The content of MMA determines the size of the spherical poly­(methyl methacrylate). Reproduced with permission from ref 478. Copyright 2021 Wiley-VCH. c) Self-growth of a silica/polymer photonic composite. A preprepared colloidal crystal composite consisting of silica nanoparticles and an acrylate-based polymer matrix (EG) is immersed in an acrylate monomer solution (0 wt % - 45.4 wt %) for growing. Reproduced with permission from ref 481. Copyright 2022 Springer Nature under CC BY 4.0 (https://creativecommons.org/licenses/by/4.0/).

Nanostructures in organisms usually can further evolve as the organism grows, for example, the color development in peacock feathers. To mimic the growable biostructure, self-grown hydrogels have been developed recently.^479,480^ A colloidal composite composed of silica nanoparticles and a growable polymer matrix is developed (Figure 35c).^481^ In the growth process, a preprepared colloidal crystal composite consisting of silica nanoparticles and an acrylate-based polymer matrix (EG) is immersed in a nutrient solution for swelling. The “nutrient solution” comprises an acrylate monomer with different concentrations and required polymerization additives. During the swelling, the sample increases in size and shifts in color depending on the monomer concentration (0 wt % - 45.4 wt %) in the nutrient solution. The shifted color and the swollen sizes are recoverable unless the products are irradiated by UV light to trigger the polymerization of the trapped nutrient solution.

#### 5.6.3 Self-Assembly of Biomolecules

Biomolecules include lipids, proteins, DNA, and RNA, etc., whose assemblies play a vital role in nearly all life processes, providing structure, information, storage, and communication within cells, and other metabolic activities.^70,482^ Their assembly process strongly depends on the molecular properties. For example, lipids, a type of amphiphilic molecules that typically consists of a hydrophilic (polar) headgroup and one or more hydrophobic alkyl tails, can form a wide variety of self-assembled structures in aqueous environments.^483^ Their assembly is driven by the hydrophobic effect: In water, the hydrophobic tails cluster together to minimize contact with the aqueous medium, leading to the formation of closed spherical bilayers known as lipid vesicles (liposomes). Various strategies have been developed to fabricate lipid vesicles. For example, thin-film hydration involves dissolving lipids in an organic solvent, evaporating the solvent to form a thin lipid film, and then hydrating the film with an aqueous buffer.^484^ This yields large multilamellar vesicles, which can be downsized via extrusion or sonication to produce small unilamellar vesicles. While applicable to a broad range of lipid compositions, this method tends to yield heterogeneous populations with low encapsulation efficiency. Another method is ethanol injection, where a lipid-ethanol solution is rapidly injected into an aqueous phase, causing instantaneous lipid self-assembly into vesicles as the ethanol dilutes.^485^ This technique can hardly offer control the size distribution. To improve the monodispersity, microfluidics is used, in which an organic solvent stream (containing lipids) and an aqueous stream are mixed in a precise, controlled manner.^484,486^ With the assistance of microfluidics, liposomes with a narrow size distribution can be produced efficiently and scalable. In addition, other strategies, such as dehydration and rehydration,^487^ reverse emulsion transfer,^488^ and electroforming,^489^ have also been widely used to prepare lipid vesicles.

The resultant lipid vesicles can further assemble into higher-order superstructures. For example, Ca^2+^-stabilized lipid vesicles can adhere to aluminum-coated substrates via Ca^2+^-mediated adhesion. Subsequent removal of Ca^2+^ induces dewetting and spontaneous formation of subcompartments, yielding colony-like protocell architectures.^490^ The assembly of vesicles can also be driven by the hydrophobic force due to the hydrophobic tail of lipids. The assembly is achieved by generating aqueous droplets encapsulated by Span-80, which is then replaced by a lipid monolayer. The obtained lipid-coated droplets are enriched in an aqueous phase via centrifugation. The hydrophobic force derived from the lipid tail drives the vesicles to dynamically assemble into millimeter-scale foams.^491^

Lipid self-assembly holds considerable scientific and practical relevance for several reasons. Beyond simple vesicles, lipid nanostructures can adopt diverse dimensionalities (0D to 3D), depending on lipid shape, tail unsaturation, and environmental conditions.^492,493^ Various structures of these lipid systems extend in multiple length scales from nano- to millimeters. These assemblies are highly biocompatible, allowing for the functionalization of the film and mimicry of the cell membrane.^494,495^ In addition, the lipid vesicles can further assemble into hierarchical structures, such as the multilamellar vesicles,^496^ bicontinuous cubics,^497,498^ and oligo-vesicular vesicles.^490^ The ability to form these complex, functional nanostructures through a spontaneous and scalable process makes the lipid assembly promising in artificial cell/tissue,^486,499^ drug delivery,^500–502^ cell biology,^496,503^ and life origin.^504^

#### 5.6.4 Summary

Naturally occurring assembly processes have inspired new ideas for addressing challenges in many fields since the assembled structures are usually optimized for specific scenarios. Mimicry of these natural assembly broaden the concept of colloidal self-assembly: Self-assembly of biomaterials facilitates the construction of structural materials with multiple-scale, high hierarchy, and multifunctionality; self-growing materials overcome the requirements of building blocks for traditional artificial self-assembly strategies; biomolecular assembly provides a platform to replicate and study life processes in a controllable manner. However, challenges for nature-mimetic self-assembly remain. For example, how different motifs found in natural materials can be assembled into desired structures, and how these strategies can be applied in practical applications and in bulk. Table 13 summarizes nature-mimetic self-assembly strategies.

**Table 13:** Summary of Nature-Mimetic Self-Assembly

Self-assembly strategies	Key parameters	Outcomes	Remarks
(Confined) Evaporation (CNC)	CNC concentration, evaporation rate, wettability of substrates.	CNC films	Several minutes to hours, film size from several to tens of cm2.
Microfluidics (CNC)	Flow rate, lipid concentration.	CNC microbeads	Several hours to days, outcome volume from several μm3 to thousands of μm3.
Roll-to-roll (CNC)	CNC concentration, pulling speed, superhydrophilic substrate, and evaporation of the solvent.	CNC films	Tens of minutes to hours, film size from tens of dm2 to hundreds of m2.
Microphase separation	Polymer concentrations, molecular weight of polymers, and the length ratio of polymer blocks.	Films of colloidal glasses with self-generated particles.	Several hours.
Coassembly of colloids and self-growing hydrogel	The concentration of the nutrient solution and the immersion time.	Films of colloidal glasses with self-growing packing lattice.	Several hours.
Thin-film hydration (lipids)	Evaporation rate, lipid concentration.	Large multilamellar vesicles	Low efficiency, tens of minutes to hours
Ethanol injection (lipids)	Injection rate, lipid concentration.	Lipid vesicles	tens of minutes, hard to control monodispersity.
Microfluidics (lipids)	Flow rate, lipid concentration.	Lipidsomes with narrow size distribution	High efficiency, narrow size distribution.

## 6 Conclusion and Challenges

The past decades have witnessed remarkable advances in colloidal self-assembly. Benefiting from advances in various strategies, colloidal assemblies with predesigned plane orientations, packing lattices, morphologies, and architectures have been constructed, broadening the structural and functional diversity of the artificial nanostructures and their application scenarios. Consequently, the field of colloidal self-assembly is transitioning from a fascinating phenomenon observed in nature to a programmable engineering discipline for building functional materials from the bottom up. Although remarkable progress has been made, colloidal self-assembly still faces considerable challenges:

### 6.1 Challenges in Fundamental Science and Process Control

i, Precision and programmability of the colloidal interaction. As mentioned in Section 2, current interactions in a colloidal system include van der Waals forces, capillary forces, electrostatic forces, depletion forces, and bonding formed by capping ligands. These interactions lack the specificity and directionality required for complex assembly. The design of interactions that are directional, specific, reversible, and dynamically responsive remains challenging.
  Patchy particles offer a promising solution for this challenge, as their anisotropic surfaces can direct selective, oriented stacking. Furthermore, bioinspired strategies provide powerful alternatives. DNA strands, for instance, enable programmable, specific, and reversible bonds for dynamically controlled assembly. Similarly, high-affinity biological pairs (e.g., streptavidin–biotin) offer an alternative route, leveraging their exceptional specificity to guide interactions.
ii, Defect control. The defects include vacancies, line defects, and plane defects. On the one hand, defects in colloidal self-assembly reduce the quality of the self-assembled entity or even destroy its PBG.^51^ On the other hand, the defects enhance light localization and light–matter interaction in the colloidal self-assembly near the defect.^388^ Consequently, constructing programmable defects in a scalable colloidal assembly could inspire new ways to manipulate light propagation.^505^
  As mentioned in Section 5.4, nanofabrication techniques have been used to introduce defects in colloidal self-assemblies. However, in a complex and costly way. Alternatively, an external field can be applied to guide particles into the desired position to form or anneal defects. Besides, introducing stimuli-responsive materials, such as self-propelled colloids, may overcome the energy barriers to introduce or avoid defects.
iii, Kinetics control. The assembly kinetics are closely related to the crystal quality of the resulting structure. A quick assembly leads to a disorder or quasi-order structure, while a slow assembly is inefficient. Precise regulation of assembling kinetics is crucial for preparing a single crystal. To solve this problem, a precisely designed interparticle potential is necessary. Moreover, the assembly procedure can be designed step-by-step so that the assembly can process in a controllable manner, avoiding the formation of quasi-ordered structures. A real-time monitoring system of the assembly process is also necessary.

### 6.2 Challenges in Structural Complexity and Functionality

i, Construction of heterogeneous structures. Currently, most colloidal strategies focus on fabricating monocomponent structures. Although strategies have been developed for fabricating heterogeneous or hierarchical colloidal structures, predesigned templates, multiple building blocks, or complicated fabrication processes are still required.^506–508^

  Recently, all-aqueous liquid–liquid phase separation has been demonstrated as a promising strategy for heterogeneous colloidal self-assembly. They overcome the requirements for predesigned substrates, multiple building blocks, and sophisticated processes. However, the programmability of the obtained structure needs to be improved. Besides, high-resolution 3D printing would enable the fabrication of various assemblies in one entity, realizing the construction of multicomponent and multifunctional structures. DNA origami would also be a promising candidate to solve this problem due to the high programmability of the DNA building blocks.
ii, Functional integrity and technology transfer. While various techniques exist for assembling colloidal nanoparticles into diverse structures, most remain confined to laboratory settings.^509^

For successful translation, these assemblies should be transferred into real-world devices that are both multifunctional and environmentally stable. Furthermore, to meet industrial demands, colloidal particles should ideally be biocompatible, biodegradable, and derived from renewable sources. In this context, biobased colloids emerge as promising candidates for next-generation structured materials, owing to their renewability, biodegradability, eco-friendliness, and capacity for functional modification. Corresponding self-assembly strategies must also be scalable, efficient, cost-effective, and environmentally sustainable to align with bioeconomy principles and support a circular framework. To facilitate the technology transfer, close collaboration between academic researchers and industrial partners is essential for systematic engineering of colloidal assemblies and the exploration of their applications. Crucially, strategies must be designed with scalability (e.g., to kilogram- or ton-scale) and cost-benefit analysis as primary considerations from the outset.

### 6.3 Challenges in Prediction of Self-Assembly

Theoretical and computational limits. Predicting the assembly pathway and final structure for complex, multicomponent systems is computationally prohibitive. “Inverse design” (defining a function and computing the required particle design) is still in its infancy. Deep learning and artificial intelligence are fascinating choices to break the limit.

All in all, the future of colloidal self-assembly lies in moving from observation to design and manufacture. The research should move beyond simple structures to complex, multicomponent, and functionally integrated industrial devices. The vision is to design colloidal “atoms” or “molecules” with specific shapes, surface patches, and interaction potentials that, when mixed under controlled conditions, spontaneously form a predetermined target structure. The colloidal self-assembly should be an area of cross-disciplinary fusion, integrating chemistry, materials, biology, and computer science.
